# QSAR-derived affinity fingerprints (part 2): modeling performance for potency prediction

**DOI:** 10.1186/s13321-020-00444-5

**Published:** 2020-06-05

**Authors:** Isidro Cortés-Ciriano, Ctibor Škuta, Andreas Bender, Daniel Svozil

**Affiliations:** 1 Centre for Molecular Informatics, Department of Chemistry, University of Cambridge, Lensfield Road, Cambridge, CB2 1EW UK; 2 Present Address: European Molecular Biology Laboratory, European Bioinformatics Institute (EMBL-EBI), Hinxton, CB10 1SD UK; 3 CZ-OPENSCREEN: National Infrastructure for Chemical Biology, Institute of Molecular Genetics of the ASCR, v. v. i., Vídeňská 1083, 142 20 Prague, Czech Republic; 4 CZ-OPENSCREEN: National Infrastructure for Chemical Biology, Department of Informatics and Chemistry, Faculty of Chemical Technology, University of Chemistry and Technology Prague, Technická 5, 166 28 Prague, Czech Republic

**Keywords:** QSAR, Affinity fingerprints, ChEMBL, Bioactivity modeling, Cytotoxicity, Drug sensitivity prediction, Drug sensitivity

## Abstract

Affinity fingerprints report the activity of small molecules across a set of assays, and thus permit to gather information about the bioactivities of structurally dissimilar compounds, where models based on chemical structure alone are often limited, and model complex biological endpoints, such as human toxicity and in vitro cancer cell line sensitivity. Here, we propose to model in vitro compound activity using computationally predicted bioactivity profiles as compound descriptors. To this aim, we apply and validate a framework for the calculation of QSAR-derived affinity fingerprints (QAFFP) using a set of 1360 QSAR models generated using K_i_, K_d_, IC_50_ and EC_50_ data from ChEMBL database. QAFFP thus represent a method to encode and relate compounds on the basis of their similarity in bioactivity space. To benchmark the predictive power of QAFFP we assembled IC_50_ data from ChEMBL database for 18 diverse cancer cell lines widely used in preclinical drug discovery, and 25 diverse protein target data sets. This study complements part 1 where the performance of QAFFP in similarity searching, scaffold hopping, and bioactivity classification is evaluated. Despite being inherently noisy, we show that using QAFFP as descriptors leads to errors in prediction on the test set in the ~ 0.65–0.95 pIC_50_ units range, which are comparable to the estimated uncertainty of bioactivity data in ChEMBL (0.76–1.00 pIC_50_ units). We find that the predictive power of QAFFP is slightly worse than that of Morgan2 fingerprints and 1D and 2D physicochemical descriptors, with an effect size in the 0.02–0.08 pIC_50_ units range. Including QSAR models with low predictive power in the generation of QAFFP does not lead to improved predictive power. Given that the QSAR models we used to compute the QAFFP were selected on the basis of data availability alone, we anticipate better modeling results for QAFFP generated using more diverse and biologically meaningful targets. Data sets and Python code are publicly available at https://github.com/isidroc/QAFFP_regression.

## Introduction

A major research question in Quantitative Structure–Activity Relationship (QSAR) has been (and still is) how to numerically encode small molecules [1–4]. Compound descriptors are generally calculated using 2-dimensional (2D) or 3-D representations of chemical structures as a starting point (although sometimes even simpler 1-D descriptors are also used, e.g., atom counts or molecular weight [5, 6]). The underlying idea when these descriptors are used to generate QSAR models is the ‘Molecular Similarity Principle’, which states that the bioactivities of structurally similar compounds tend to be correlated more often than those of dissimilar ones [7, 8]. Although compound descriptors based on the chemical structure are customarily used today in similarity searching and QSAR, they suffer from the limitation that they can only provide accurate predictions for structurally similar compounds, where the above principle holds. However, in case of e.g., ‘activity cliffs’ [9], different scaffolds binding to the same protein, or different binding sites, there are exceptions to this principle, and molecular descriptors based purely on molecular structure will not be sufficiently information-rich in order to handle such multi-modal models very well. Hence, the information that can be gathered about the bioactivities of structurally dissimilar compounds on the basis of their chemical structure alone is often limited.

A conceptually different approach is the quantification of compound similarity on the basis of the similarity of their bioactivity profiles instead of the similarity of their chemical structures [10–12]. The underlying principle, *similarity in bioactivity space*, is that compounds displaying correlated bioactivity endpoints across a set of assays (e.g., assays based on the activity of a purified protein, or cell-based assays) are likely to display similar activities also on other assays (which conceptually can then be modelled as a linear combination, or more complex function, of the input assay panel activities [13]). The set of bioactivities across a panel of assays are usually known as affinity, bioactivity, protein or high-throughput screening fingerprints [12–15]. Note that the term ‘affinity fingerprint’ is often used even when the bioactivity endpoints are not K_i_ nor K_d_ values, but rather assay-specific metrics of potency, such as IC_50_ or EC_50_ values, so it comprises a broad set of activity spectra-based descriptors. In the following and in the accompanying manuscript, we use the term affinity fingerprint to refer to the set of biological endpoints, experimentally determined or predicted, irrespective of whether the endpoint measured corresponds to a potency or an affinity metric. For a comprehensive review of existing methods to predict affinity fingerprints using existing high-throughput data [16–28], the reader is referred to the introduction of the accompanying manuscript [29].

Affinity fingerprints encode information about the many interactions (both strong and weak) between a given compound and its targets, and thus permit to model complex biological endpoints, such as human toxicity and in vitro cancer cell line sensitivity, and provide complementary signal to chemical structure information [30, 31]. Current predictive methods use either structural information of compounds as descriptors to model their activity on a single target (i.e., QSAR [32]), or assay activity as covariates to model the activity of a single compound across a target panel [33–35]. The latter strategy suffers from the limitation that in order to make predictions on novel targets these need to be experimentally profiled in the same way as those in the training set. In contrast, QSAR methods permit, to the extent the training data allows extrapolation in chemical space [36], to make predictions on new molecules more scalable, as the computation of compound descriptors only requires the chemical structure as input. Therefore, designing computational tools to model assay readouts using the chemical structure of compounds as input would permit to predict in silico the affinity fingerprint for a molecule of interest without the need for experimental testing, which in turn could provide a better description of the relevant variance connecting chemical and biological space.

**Fig. 1:**
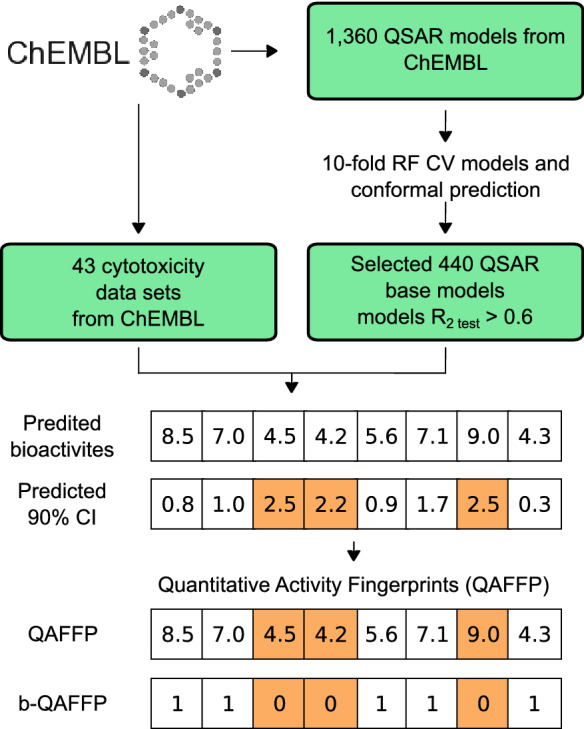
Overview of the workflow used in this study. We initially assembled and modelled 1360 data sets from ChEMBL database using RF (Random Forest) and conformal prediction. Of these, 440 displayed high predictive power in cross validation (q^2^ > 0.5) and on external molecules (R^2^_test set_ > 0.6), and hence, were selected to build QAFFP fingerprints for 18 cytotoxicity and 25 protein target data sets (Tables 1 and 2) assembled from ChEMBL database as well. To benchmark the predictive signal of QAFFP, we compared the performance of RF models trained on QAFFP against models generated using Morgan2 fingerprints or 1-D and 2-D physicochemical descriptors across a diverse set of 43 bioactivity data sets (Tables 1 and 2)

The construction of such in silico affinity fingerprints, termed QSAR-derived Quantitative Affinity Fingerprints (QAFFPs), is described in the accompanying manuscript [29] where their performance for similarity searching, compound activity classification and scaffold hopping is reported. The range of application of QAFFP is further enhanced in the present manuscript, in which the use of QAFFPs in regression settings is studied. Specifically, QAFFPs were assessed to model in vitro potency on a continuous scale across 43 diverse data sets (Fig. 1 and Tables 1, 2). For each compound in each data set, the predictions generated by a set of 1360 QSAR models (termed base models) trained on IC_50_, EC_50_, K_i_ and K_d_ data were recorded. These vectors of predicted activities, i.e., QAFFP, were then used as compound descriptors to build QSAR models. To benchmark the predictive power of QAFFP, we assembled 18 diverse cytotoxicity data sets from ChEMBL, and constructed QSAR models using cross-validation. In addition, we also used 25 additional protein target QSAR data sets from ChEMBL for validation. The compounds were encoded using either circular Morgan fingerprints [37], 1-D and 2-D physicochemical descriptors, or QAFFP fingerprints. Hence, this framework allowed us to evaluate the predictive power of QAFFP fingerprints across a wide range of bioactivity prediction models.

**Table 1:** Cytotoxicity data sets used in this study

Cell line	Cell line description	ChEMBL assay ID	Cellosaurus ID	Organism of origin	Number of bioactivity data points
A2780	Ovarian carcinoma cells	CHEMBL3308421	CVCL_0134	Homo sapiens	2255
CCRF-CEM	T-cell leukemia	CHEMBL3307641	CVCL_0207	Homo sapiens	3047
DU-145	Prostate carcinoma	CHEMBL3308034	CVCL_0105	Homo sapiens	2512
HCT-116	Colon carcinoma cells	CHEMBL3308372	CVCL_0291	Homo sapiens	6231
HCT-15	Colon adenocarcinoma cells	CHEMBL3307945	CVCL_0292	Homo sapiens	994
HeLa	Cervical adenocarcinoma cells	CHEMBL3308376	CVCL_0030	Homo sapiens	7532
HepG2	Hepatoblastoma cells	CHEMBL3307718	CVCL_0027	Homo sapiens	3897
HL-60	Promyeloblast leukemia cells	CHEMBL3307654	CVCL_0002	Homo sapiens	4637
HT-29	Colon adenocarcinoma cells	CHEMBL3307768	CVCL_0320	Homo sapiens	5630
K562	Erythroleukemia cells	CHEMBL3308378	CVCL_0004	Homo sapiens	4160
KB	Squamous cell carcinoma	CHEMBL3307959	CVCL_0372	Homo sapiens	2731
L1210	Lymphocytic leukemia cells	CHEMBL3308391	CVCL_0382	Mus musculus	4873
LoVo	Colon adenocarcinoma cells	CHEMBL3307691	CVCL_0399	Homo sapiens	1120
MCF7	Breast carcinoma cells	CHEMBL3308403	CVCL_0031	Homo sapiens	12,001
MDA-MB-231	Breast epithelial adenocarcinoma cells	CHEMBL3307960	CVCL_0062	Homo sapiens	3482
NCI-H460	Non-small cell lung carcinoma	CHEMBL3307677	CVCL_0459	Homo sapiens	2277
PC-3	Prostate carcinoma cells	CHEMBL3307570	CVCL_ NIRG—MRC0035	Homo sapiens	4294
SK-OV-3	Ovarian carcinoma cells	CHEMBL3307746	CVCL_0532	Homo sapiens	1589

**Table 2:** Protein target data sets used in this study

Target preferred name	Target abbreviation	Uniprot ID	ChEMBL ID	Number of bioactivity data points
Alpha-2a adrenergic receptor	A2a	P08913	CHEMBL1867	203
Tyrosine-protein kinase ABL	ABL1	P00519	CHEMBL1862	773
Acetylcholinesterase	Acetylcholinesterase	P22303	CHEMBL220	3159
Androgen Receptor	Androgen	P10275	CHEMBL1871	1290
Serine/threonine-protein kinase Aurora-A	Aurora-A	O14965	CHEMBL4722	2125
Serine/threonine-protein kinase B-raf	B-raf	P15056	CHEMBL5145	1730
Cannabinoid CB1 receptor	Cannabinoid	P21554	CHEMBL218	1116
Carbonic anhydrase II	Carbonic	P00918	CHEMBL205	603
Caspase-3	Caspase	P42574	CHEMBL2334	1606
Thrombin	Coagulation	P00734	CHEMBL204	1700
Cyclooxygenase-1	COX-1	P23219	CHEMBL221	1343
Cyclooxygenase-2	COX-2	P35354	CHEMBL230	2855
Dihydrofolate reductase	Dihydrofolate	P00374	CHEMBL202	584
Dopamine D2 receptor	Dopamine	P14416	CHEMBL217	479
Norepinephrine transporter	Ephrin	P23975	CHEMBL222	1740
Epidermal growth factor receptor erbB1	erbB1	P00533	CHEMBL203	4868
Estrogen receptor alpha	Estrogen	P03372	CHEMBL206	1705
Glucocorticoid receptor	Glucocorticoid	P04150	CHEMBL2034	1447
Glycogen synthase kinase-3 beta	Glycogen	P49841	CHEMBL262	1757
HERG	HERG	Q12809	CHEMBL240	5207
Tyrosine-protein kinase JAK2	JAK2	O60674	CHEMBL2971	2655
Tyrosine-protein kinase LCK	LCK	P06239	CHEMBL258	1352
Monoamine oxidase A	Monoamine	P21397	CHEMBL1951	1379
Mu opioid receptor	Opioid	P35372	CHEMBL233	840
Vanilloid receptor	Vanilloid	Q8NER1	CHEMBL4794	1923

## Methods

### Data collection and curation

We gathered IC_50_ data for 18 cancer cell lines from ChEMBL database version 23 using the *chembl_webresource_client* python module [38–40]. To gather high-quality data, we only kept IC_50_ values corresponding to molecules that satisfied the following filtering criteria [41]: (i) molecule type equal to “Small molecule”, (ii) activity unit equal to “nM”, and (iii) activity relationship equal to “=”. The average pIC_50_ value was calculated when multiple IC_50_ values were annotated for the same compound-cell line pair. IC_50_ values were modeled in a logarithmic scale (pIC_50_ = − log_10_ IC_50_ [M]). Further information about the data sets is given in Table 1 and in a previous study by the authors [42]. We also collected 25 QSAR data sets for validation from previous work by the authors (Table 2) [42–44]. All data sets used in this study, as well as the code required to generate the results presented herein, are publicly available at https://github.com/isidroc/QAFFP_regression. The distribution of bioactivity values for all data sets is reported in Additional file 1: Figure S1.

### Molecular representation

The Innovative Medicines Initiative eTOX project standardizer (https://github.com/flatkinson/standardiser) was used to normalize all chemical structures reported here to a common representation scheme using the default options. This normalization step is crucial for the generation of compound descriptors, as these (except for e.g., heavy atom counts) generally depend on a consistent representation of molecular properties, such as aromaticity of ring systems, tautomer representation or protonation states. Entries corresponding to entirely inorganic structures were removed. In the case of organic molecules, the largest fragment was kept in order to filter out counterions following standard procedures in the field [45, 46], and salts were neutralized.

### QSAR-based activity fingerprints (QAFFP)

The protocol to calculate QSAR-based affinity fingerprints using ChEMBL data is explained in detail in the accompanying manuscript [29]. In brief, the workflow can be summarized in the following five steps (Fig. 1):

1. We initially gathered a total of 1360 QSAR data sets from ChEMBL version 19. We considered both human and non-human protein targets, and EC_50_ (173 targets), IC_50_ (786), K_i_ (365), and K_d_ (36) values as bioactivity endpoints. We only considered measurements with an activity relationship equal to ‘=’ and activity values reported in ‘nM’ units. The mean value was used as the activity value when multiple measurements were annotated for the same compound-protein pair only if the standard deviation of all annotated measurements was lower than 0.5; otherwise the data point was not further considered.
2. To model these data sets, we trained tenfold CV RF models using 30% of the data as the test set, and Morgan2 fingerprints as compound descriptors. We term these models *base models*. A total of 440 models (376 unique targets) displayed an average R^2^_test_ value > 0.6, and a cross-validation q^2^ value > 0.5. These cut-off values are a reasonable choice to identify models with high predictive power on unseen data (although we note that the minimum predictive power required for a model to be useful in practice depends on the context in which it is applied, e.g., poor predictive performance might be useful in hit identification but not in lead optimization) [47].
3. The cross-validation predictions served to build a cross-conformal predictor for each of the 1360 base models as previously described [48].
4. To calculate QAFFP for the compounds in the 18 cytotoxicity and 25 protein target data sets, we used the base models to calculate point predictions (i.e., IC_50_, EC_50_, K_d_, and K_i_ values), and calculated confidence intervals (90% confidence) for each individual prediction using the corresponding conformal predictor. Hence, for a given compound we computed (i) a 1360-dimensional fingerprint, where each bit corresponds to the predicted activity for that compound using one of the 1360 base models considered, and (ii) a 1360-dimensional vector recording the prediction errors calculated using conformal prediction.
5. Next, we combined the point predictions and the predicted confidence intervals to define three types of QAAFP (Fig. 1):
  Real-valued QAFFP (rv-QAFFP): This type of fingerprint is defined by the point predictions computed using the base models. We defined two types of rv-QAFFP fingerprints: “rv-QAFFP 440” fingerprints were computed using the 440 base models showing high predictive power on unseen data as explained above, whereas “rv-QAFFP 1360” fingerprints were calculated using the 1360 base models irrespective of their predictive power on the test set.
  Binary QAFFP (b-QAFFP 440): To construct “b-QAFFP 440” fingerprints we set to one 1 all positions in the rv-QAFFP 440 fingerprint corresponding to predictions lying above a given activity cutoff (in this case 5 pIC_50_ units), and which are within the applicability domain (AD) of the underlying base model. We consider that a prediction is within the AD of a base model if the predicted confidence interval is lower than 2 pIC_50_ units, (i.e., the predicted confidence interval is no wider than +/− 2). Thus, all values that lie below the affinity cutoff but are still within model AD were encoded using zeros. The value was set to zero as well for predictions lying outside the model AD, following the assumption that a compound is more likely to be inactive than active. Thus, this corresponds to setting to one those bits corresponding to the targets with which a given compound is predicted to interact even at low compound concentrations, while also taking into account the confidence of the prediction.

In the case of the 25 protein target data sets (Table 2), base models trained on bioactivity data from these targets or their orthologues were excluded, and thus not considered to compute QAFFP for these data sets.

As a baseline method for comparisons, we considered RF models trained on Morgan fingerprints [37], and physicochemical descriptors. We computed circular Morgan fingerprints using RDkit (release version 2013.03.02) [37, 49]. The radius was set to 2 and the fingerprint length to 1024. Thus, we refer to Morgan fingerprints as Morgan2 hereafter. Morgan fingerprints encode compound structures by considering radial atom neighborhoods. The choice of Morgan fingerprints as a base line method to compare the performance of QSAR-derived affinity fingerprints was motivated by the high retrieval rates obtained with Morgan fingerprints in benchmarking studies of compound descriptors [50, 51]. A total of 200 1-D and 2-D physicochemical descriptors (abbreviated as Physchem hereafter) were also computed using RDkit and used to generate QSAR models. We also combined Morgan2 fingerprints, physicochemical descriptors, and QAFFP to define combined descriptors, namely: rv-QAFFP 440 and Morgan2, rv-QAFFP 440 and Physchem, b-QAFFP 440 and Morgan2, b-QAFFP 440 and Physchem, rv-QAFFP 1360 and Morgan2, rv-QAFFP 1360 and Physchem. Thus, we considered a total of 11 types of descriptors to encode the compounds.

The Jaccard-Needham dissimilarity between pairs of compounds was computed using the function *scipy.spatial.distance.jaccard* from the python library SciPy [52].

### Model training and performance evaluation

We trained Random Forest (RF) models using tenfold CV on 70% of the data selected at random. The performance was evaluated on the remaining 30% of the data (i.e., test set) by calculating the root mean squared error (RMSE) and the Pearson correlation coefficient (R^2^) for the observed against the predicted pIC_50_ values. We trained 50 models for all combinations of factor levels, giving rise to 23,650 models (43 data sets × 11 descriptors sets × 50 replicates). In each replicate, a different subset of the data was selected as the test set. The composition of the training and test sets across the 50 replicates for a given data set was the same for all fingerprint types. RF models and feature importance values were computed using the *RandomForestRegressor* class from the python library Scikit-learn [53].

RF are generally robust across a wide range of parameter values. In practice, a suitable choice for the number of trees in the Forest (*n*_trees_) was shown to be 100 in previous work [54–56], as higher values do not generally lead to significantly higher predictive power, which we found to be also the case for these data sets (Additional file 1: Figure S2). Hence, we trained the RF models using 100 trees and the default values for all other parameters.

### Experimental design

To benchmark the predictive power of QAFFP, Morgan2 fingerprints and physicochemical descriptors in a statistically robust manner we designed a balanced fixed-effect full-factorial experiment with replications [57]. We considered two factors, namely: (i) *data set*: 43 data sets considered, and (ii) *descriptor*: rv-QAFFP 440, b-QAFFP 440, rv-QAFFP 1360, Morgan2, Physchem, rv-QAFFP 440 and Morgan2, rv-QAFFP 440 and Physchem, b-QAFFP 440 and Morgan2, b-QAFFP 440 and Physchem, rv-QAFFP 1360 and Morgan2, rv-QAFFP 1360 and Physchem. In addition, we included an interaction term between the factors descriptor and data set to examine whether the performance of the descriptor types used vary across data sets.

This factorial design was studied with the following linear model:

$$\begin{aligned} & RMSE \, test \, set = data set_{i} + descriptor_{j} + \left( {data set*descriptor} \right)_{i,j} + \mu_{0} + \varepsilon_{i,j,k} \\ & \left( {i \in \left\{ {1, \ldots , N_{data sets} = 43} \right\}; j \in \left\{ {1, \ldots , N_{descriptors} = 11} \right\};} \right. \\ & \left. { k \in \left\{ {1, \ldots , N_{replicates} = 50} \right\};} \right) \\ \end{aligned}$$

where the response variable, *RMSE*_*i*,*j*,*k* test_, corresponds to the RMSE value for the predicted against the observed activities on the test set for a given data set, descriptor type and replicate. The factor levels “ovarian carcinoma cells A2780” (*data set*), and “Morgan2” (*descriptor*), were used as reference factor levels to calculate the intercept term of the linear model, μ_0_, which corresponds to the mean RMSE_test_ value for this combination of factor levels. The coefficients (slopes) for the other combinations of factor levels correspond to the difference between their mean RMSE_test_ value and the intercept. The error term, $\upepsilon$_*i*,*j*,*k*_, corresponds to the random error of each RMSE_test_ value, which are defined as ${\upepsilon _{i,j,k}} = {\text{RMSE}}_{i,j,k}-{\text{mean}} ({\text{RMSE}}_{i,j})$. These errors are assumed to (i) be mutually independent, (ii) have an expectation value of zero, and (iii) have constant variance. The use of a linear model to assess the predictive power of QAFFPP, Morgan2 fingerprints and Physchem descriptors allowed to control for the variability across data sets, and to avoid that results were biased by elements such as the number of datapoints, data set modellability, etc.

The normality and homoscedasticity assumptions of the linear model were assessed with (i) quantile–quantile (Q–Q) plots and (ii) by visual inspection of the RMSE_test_ distributions, and (iii) by plotting the fitted RMSE_test_ values against the residuals [57]. Homoscedasticity means that the residuals are evenly distributed (i.e., equally dispersed) across the range of the RMSE_test_ values considered in the linear model. It is essential to examine this assumption to guarantee that the modeling errors (i.e., residuals) and the dependent variable are not correlated. A systematic bias of the residuals would indicate that they are not consistent with random error, and hence, they contain predictive information that should be included in the model.

## Results and discussion

**Fig. 2:**
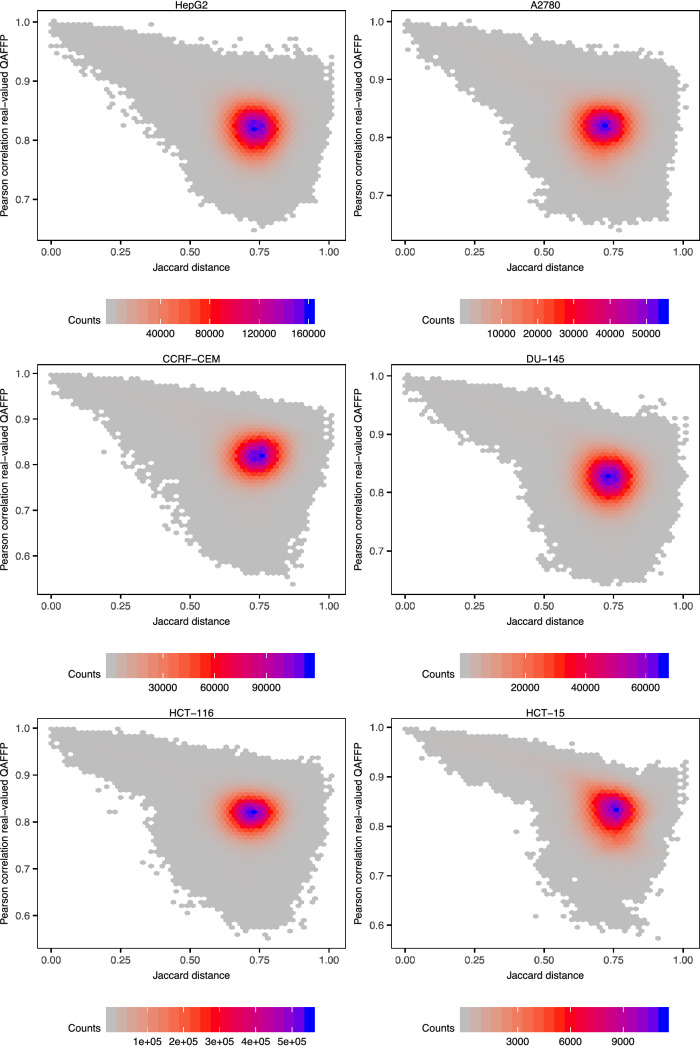
Jaccard-Needham dissimilarity calculated using Morgan2 fingerprints, against Pearson correlation values calculated using rv-QAFFP 440 for all pairs of compounds in each data set. Only a randomly picked subset of the 18 cytotoxicity data sets is shown for illustration. Similar results were obtained for the other data sets

**Fig. 3:**
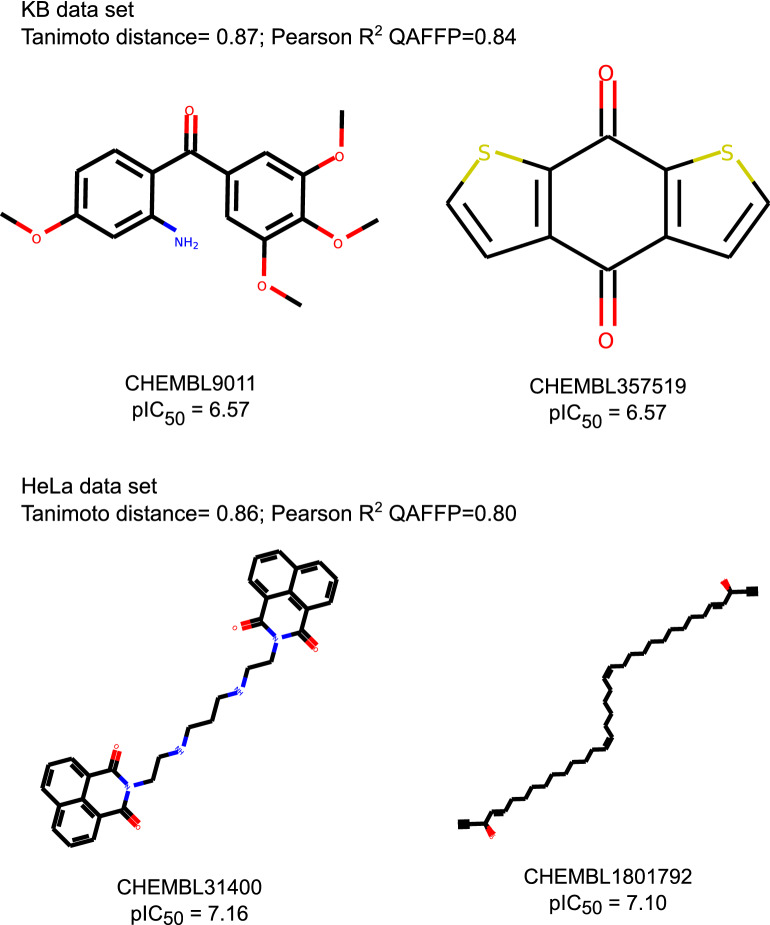
Examples of structurally dissimilar compounds, showing correlated rv-QAFFP 440 and similar pIC_50_ values. These examples illustrate that the similarity in bioactivity space is captured by the rv-QAFFP 440 even for structurally dissimilar compounds, underlining the importance of using multi-modal representations of chemical structures, beyond similarity in chemical descriptor space alone

We initially sought to examine the differences between Morgan2 fingerprints and QAFFP in terms of how they encode the chemical space. To this aim, for each pair of compounds in the 18 cytotoxicity data sets considered (Table 1) we computed pairwise Jaccard-Needham dissimilarity values [52, 58] using Morgan2 fingerprints (similarity in chemical space; x-axis in Fig. 2), and pairwise Pearson correlation coefficients using rv-QAFFP 440 fingerprints (similarity in bioactivity space; y-axis in Fig. 2). Overall, we observe a negative and significant correlation (Pearson correlation, *P* < 0.001) between Jaccard-Needham dissimilarity and correlation in bioactivity space for all data sets. The pairwise correlation values calculated using rv-QAFFP are, as expected, highly correlated for pairs of structurally similar compounds (i.e., showing a low Jaccard-Needham dissimilarity; upper left-hand quadrant in the panels in Fig. 2). These results are consistent with the fact that QAFFP are computed using base models trained on Morgan2 fingerprints and with the similarity principle, as structurally similar compounds are expected to show correlated bioactivity profiles. A substantial fraction of compound pairs showing a relatively large degree of structural dissimilarity (Jaccard-Needham dissimilarity ~ 1) show high similarity in bioactivity space (upper right-hand quadrant in Fig. 2). For instance, compounds CHEMBL357519 and CHEMBL9011 (first row in Fig. 3) display comparable activities on the cell line KB (pIC_50_ = 6.57 and 7.56, respectively), and a Jaccard-Needham dissimilarity of 0.87. However, their rv-QAFFPs are highly correlated, with a Pearson correlation coefficient of 0.84 (*P* < 0.05). Another example is the pair of compounds CHEMBL31400 and CHEMBL1801792 in the HeLa data set (second row in Fig. 3), with pIC_50_ values of 7.16 and 7.10, respectively, a Jaccard-Needham dissimilarity of 0.86, and highly correlated rv-QAFFP 440 values (Pearson R^2^ = 0.80, *P *< 0.05). Overall, these results show that structurally dissimilar compounds displaying comparable pCI_50_ values (given the uncertainty of pIC_50_ data [41]) are often clustered closely in bioactivity space, as quantified by the correlation between their rv-QAFFP 440 values. This is also allowed according to the ‘Neighbourhood Behavior’ principle [59], which states that while similar molecules are expected to behave similarly or average, dissimilar molecules may display either dissimilar, but in some cases also similar properties.

**Fig. 4:**
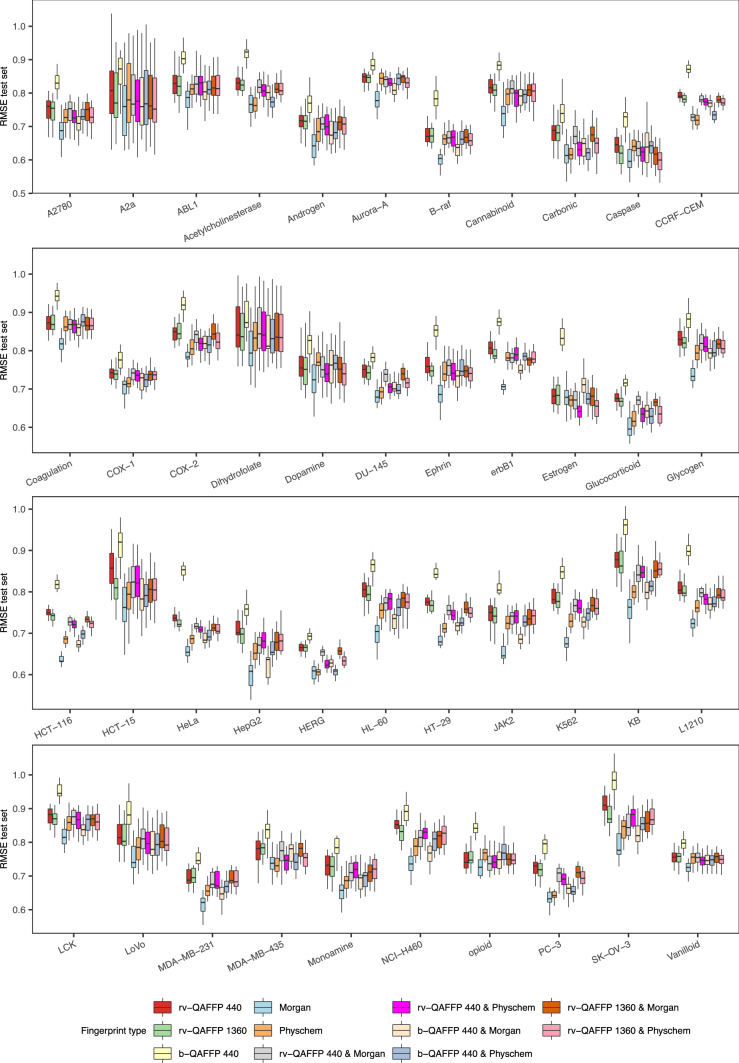
RMSE_test_ values calculated with models trained on each of the 11 descriptor types considered across the 43 data sets modelled in this study (18 cytotoxicity and 25 protein data sets; Tables 1 and 2). We trained 50 models for each combination of descriptor type and data set, each time holding a different subset of the data as test set. Overall, predictive models were obtained for all descriptor types, and the performance of different descriptor types varied across data sets modelled

To test whether encoding compounds using rv-QAFFP improves the modeling of compound activity, we generated RF models for the 18 cytotoxicity data sets (Table 1), as well as for 25 protein target data sets (Table 2) [42]. As a baseline for comparisons, we trained RF models using Morgan2 fingerprints or physicochemical descriptors, and quantified performance by calculating the RMSE and R^2^ values for the observed against the predicted pIC_50_ values for the compounds in the test set (Fig. 4 and Additional file 1: Figure S3). The average R^2^_test_ values (*n *= 50) were above 0.6 for all data sets, indicating that Morgan2 fingerprints and physicochemical descriptors capture the aspects of molecular structure related to bioactivity, and hence permit to model compound activity for these data sets satisfactorily.

We used the same modeling strategy to generate RF models using three types of QAFFP (b-QAFFP 440, rv-QAFFP 440, and rv-QAFFP 1360), and QAFFPs combined with Morgan2 fingerprints and physicochemical descriptors (see “Methods”). Overall, the models trained on QAFFP showed high predictive power, with R^2^ values in the 0.5–0.9 range, and RMSE values in the ~ 0.6–0.95 pIC_50_ units range (Fig. 4 and Additional file 1: Figure S3). These values are in agreement with the expected model performance given the uncertainty of pIC_50_ data from ChEMBL; i.e., the maximum Pearson correlation coefficient when modeling IC_50_ data from ChEMBL, which was estimated to be in the 0.51–0.85 range [41, 60]. Finally, we performed Y-scrambling experiments for all data sets [61]. To this aim, we shuffled the bioactivity values for the training set instances before model training. We obtained R^2^ values around 0 (P < 0.001) in all Y-scrambling experiments we performed (Additional file 1: Figure S4). Therefore, these results indicate that the predictive power of the models trained on QAFFP is not a consequence of spurious correlations.

**Fig. 5:**
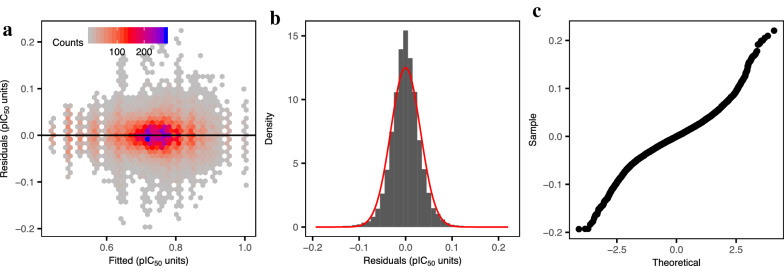
Verification of the linear model assumptions. **a** Assumption of homoscedasticity of the residuals. Fitted values against the residuals. The residuals are centered around zero and, roughly, present a comparable dispersion across the range of values considered, indicating that the assumption of the homoscedasticity of the residuals is fulfilled. Assumption of the normality of the residuals, assessed with the distribution of the residuals (**b**) and a quantile–quantile (Q–Q) plot (**c**). The residuals follow a Gaussian distribution with zero mean, indicating that the assumption of the normality of the residuals is fulfilled. Overall, these results indicate that the assumptions of the linear model are fulfilled

To assess the relative performance of the 11 descriptor types defined in a statistically robust manner, we designed a factorial experiment (see “Methods”). The fitted linear model displayed an R^2^ value adjusted for the number of parameters equal to 0.90, and a standard error for the residuals equal to 0.03 (*P* < 10^−15^), indicating that the variability of model performance on the test set can be explained to a large extent by the data set and descriptor type used. The values for the coefficients, namely slopes and intercept, and their *P* values are reported in Additional file 2: Table S1. The verification of the model assumptions is reported in Fig. 5. We did not include the percentage of the data included in the test set as a covariate in the linear model because we observed that the relative performance of the descriptor types considered was overall constant across models trained on increasingly larger fractions of the data (Additional file 1: Figure S5).

The factorial analysis revealed a significant interaction between the factors *data set* and *descriptor type* (*P* < 10^−15^) indicating that the predictive power of the descriptor types considered varies across data sets. This can be observed in Fig. 4 as well, as the distances between the boxes vary across data sets; i.e., a given descriptor type leads to the highest predictive power for some data sets but not in others. For instance, the average RMSE value for rv-QAFFP 440 (red box) is higher than that corresponding to Morgan2 fingerprints (light blue box) when modeling the cell line data set A2780; however, the opposite is observed for the data set Cannabinoid (first panel in Fig. 4). Interestingly, combining Morgan2 fingerprints and QAFFP did not increase model performance, and models trained on the binary form of QAFFP (b-QAFFP) constantly led to lower predictive power as compared to rv-QAFFP (Fig. 4 and Additional file 1: Figure S3). Overall, these results suggest that the predictive signal provided by both fingerprints, at least when using RF, does not seem to be complementary.

**Fig. 6:**
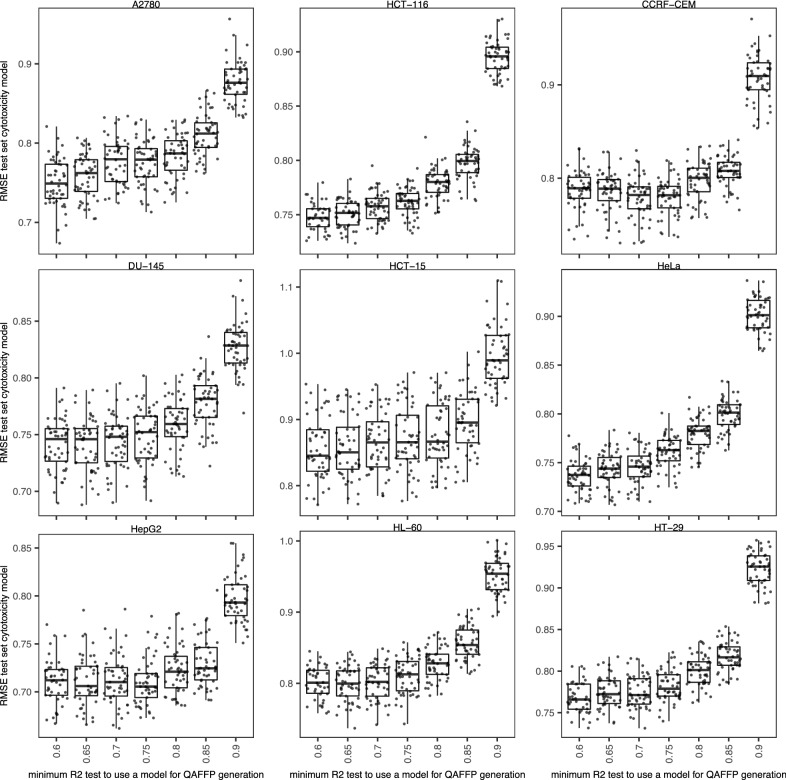
RMSE_test_ values obtained with models trained on rv-QAFFP fingerprints calculated using only those base models with R^2^ values on the test set greater or equal than the cut-off value indicated in the x-axis. Each point corresponds to a replicate. Overall, the predictive power on the test set declines as the set of models included to generate the rv-QAFFP is reduced

**Fig. 7:**
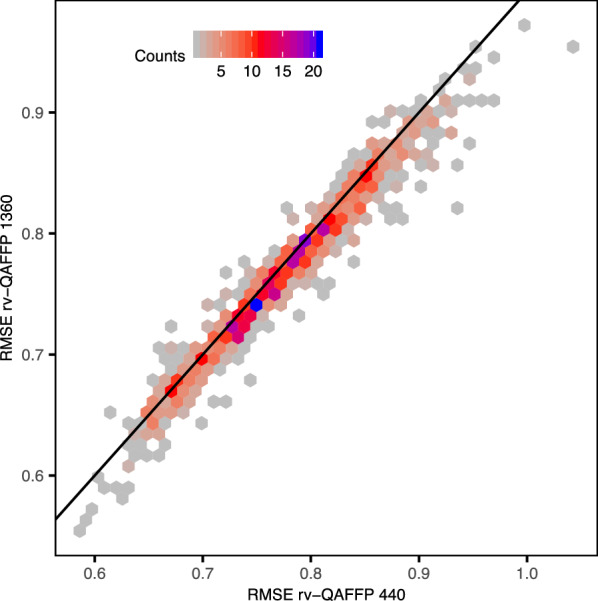
RMSE on the test set for predictive models trained on either rv-QAFFP 440 or rv-QAFFP 1360. The results for the 43 data sets and 50 replicates are shown. Overall, it can be seen that the performance of models trained on rv-QAFFP computed using base models with low and high predictive power (i.e., rv-QAFFP 1360) is comparable to the performance of models trained on rv-QAFFP (i.e., rv-QAFFP 440) computed using only base models showing high predictive power

Given the substantial diversity in performance of the 1360 base models used to generate QAFFP [29], we next sought to investigate whether we could better model the 18 cytotoxicity data sets by computing rv-QAFFP using only those base models showing high predictive power. To this end, we used increasingly higher cut-off values for the minimum R^2^_test_ value a base model needs to show to be considered for the calculation of rv-QAFFP. That is, we hypothesized that removing from the rv-QAFFP those bits corresponding to moderately predictive base models might lead to a less noisy rv-QAFFP and a better description of the relevant variance connecting chemical and biological space, thus increasing predictive power. We found that rv-QAFFP built using base models with R^2^_test_ > 0.60–0.65 lead to the lowest average RMSE_test_ values for the 18 cytotoxicity data sets (Fig. 6). Models trained on rv-QAFFP values generated with highly predictive base models (R^2^_test_ > 0.8) only, leaving 76 based models to compute QAFFP, increased the average RMSE_test_ values by ~ 12–20%. One explanation for this might be that increasing the dimensionality of the QAFFP by including low predictive base models adds predictive signal, even if these generate inaccurate predictions [13]. However, we observed that including all 1360 base models to compute QAFFP (i.e., rv-QAFFP 1360) did not increase predictive power on the test set, indicating that base models with low predictive power do not add additional predictive signal (Fig. 7). It is also important to consider that RF models are generally robust to moderate noise levels when modeling QSAR data sets, and hence, low levels of noise are well tolerated, and, in fact, might even help to generate models robust to noisy input data [62, 63]. Together, these results indicate that although the predictions generated by moderately predictive base models might be noisy, they better explain the relevant variance connecting chemical and biological space [13], and that including base models with low predictive power does not add additional predictive signal to improve the modelling of these data sets.

**Fig. 8:**
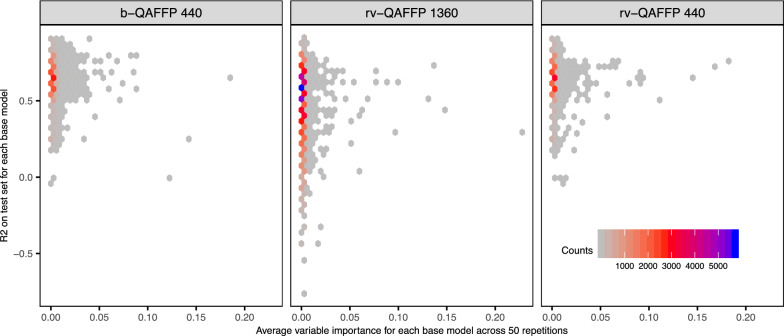
Analysis of feature importance. The variable importance averaged across 50 replicates for each feature in the QAFFP (i.e., base model) is shown against the predictive power of each base model in cross validation calculated during the training of base models. Overall, a correlation between predictive power and feature importance was not observed

We next analysed the predictive signal provided by each bit in the QAFFP, each one corresponding to a different base model, using the feature importance functionality of Random Forest models [64]. We did not observe a correlation between the predictive power of base models and the estimated variable importance across the 50 models generated for each descriptor and data set combination (Fig. 8). The contribution of each descriptor was variable across data sets, and in none of the cases the predictive power was driven by the contribution of few base models, but rather by the combination of weak contribution from many models (Additional file 1: Figure S6).

Finally, we sought to investigate whether the activity of some compounds is better modelled by QAFFP in comparison to Morgan2 fingerprints. Such an evidence would support the use of QAFFP instead of, or in addition to, other compound descriptors in predictive bioactivity modeling. To this, we examined the correlation between the errors in prediction on the test set calculated with models trained on either Morgan2 fingerprints, one of the three types of QAFFP considered, or combinations thereof. It was found that 40.1–56.6%, and 73.7–86.2% of the test set instances were predicted with an absolute error in prediction below 0.5 and 1.0 pIC_50_ units, respectively by both Morgan2 and QAFFP-based models (Table 3). Although less than 0.5% of the test set instances were predicted with variable errors in prediction across models trained on different fingerprint types, the error in prediction for some compounds varies > 2 pIC_50_ units depending on the fingerprint type used for modeling (Additional file 1: Figures S7, S8). For instance, the error in prediction for compound CHEMBL2420625 is 1.93 pIC_50_ units (σ = 0.29; *n *= 50) for the Morgan2-trained model, whereas the error drops to 0.69 pIC_50_ units (σ = 0.16; *n *= 50) pIC_50_ units for models trained on QAFFP (Additional file 1: Figure S8).

**Table 3:** Percentage of instances in the test set predicted using Morgan2-based and rv-QAFFP-based models showing an Absolute Error in Prediction (AEP) meeting the cut-off values indicated in the header

Dataset	AEP Morgan2 and AEP rv-QAFFP 440 ≤ 0.5	AEP Morgan2 and AEP rv-QAFFP 440 ≤ 1.0	AEP Morgan2 ≥ 1.0 and AEP rv-QAFFP 440 ≤ ≤0.5	AEP Morgan2 ≤ 0.5 and AEP rv-QAFFP 440 ≤ ≥ 1.0	AEP Morgan2 ≥2.0 and AEP rv-QAFFP 440 ≤ ≤ 1.0	AEP Morgan2 ≤ 1.0 and AEP rv-QAFFP 440 ≤ ≥ 2.0	AEP Morgan2 ≥ 3.0 and AEP rv-QAFFP 440 ≤≤ 1.0	AEP Morgan2 ≤ 1.0 and AEP rv-QAFFP 440 ≤ ≥ 3.0
A2780	49.2	81.1	0.7	1.4	0.1	0.3	0.0	0.0
CCRF-CEM	46.3	78.1	0.5	1.7	0.1	0.2	0.0	0.0
DU-145	52.4	83	0.4	1.4	0.1	0	0	0
HCT-116	47.2	81.3	0.3	2.3	0	0.2	0	0
HCT-15	40.2	74.7	1	3.8	0.2	0.3	0	0
HeLa	49.9	83.1	0.5	1.8	0.1	0.2	0	0
HepG2	56.6	86.2	0.4	2.3	0.1	0.5	0	0.1
HL-60	47.9	80.9	0.4	2.4	0	0.4	0	0
HT-29	49.2	80.9	0.5	1.6	0	0.1	0	0
K562	44.7	79.5	0.5	2.6	0	0.2	0	0
KB	40.1	74.6	0.6	3.1	0.1	0.5	0	0.1
L1210	42.6	76.4	0.5	2.4	0.1	0.2	0	0
LoVo	49.3	79.8	0.6	1.7	0	0.1	0	0
MCF7	50.1	82.8	0.4	2.8	0.1	0.4	0	0.1
MDA-MB-231	53.7	84.9	0.3	1.3	0	0.1	0	0
NCI-H460	43.6	75.6	0.9	3.1	0.1	0.5	0	0.2
PC-3	53.6	85.1	0.4	1.6	0	0.1	0	0
SK-OV-3	40.6	73.7	0.7	4.1	0.1	0.4	0	0.1

To assemble the 18 cytotoxicity data sets used here, we included all IC_50_ values for a given cell line satisfying the stringent criteria described in “Methods” irrespective of the cytotoxicity assay used. We have previously shown that cytotoxicity measurements might vary considerably cross cytotoxicity assays [41]; others have shown that different biological conclusions might be obtained depending on the parameterization of the dose–response curves [65–69]. Hence, we anticipate that the performance of QAFFP might be higher when modeling proprietary bioactivity data generated under uniform experimental conditions and data analysis pipelines [15, 27]. Our results show that compound activity can be modelled on a continuous scale using the predicted activities on an unbiased selection of protein targets as compound descriptors. However, we note in particular that the base models used to generate QAFFP were selected on the basis of the amount of bioactivity data available in ChEMBL only, and on whether they could be satisfactorily modelled using Morgan2 fingerprints. Hence, no biological criteria were considered. As more public data become available, it will be possible to test whether including targets with high network connectivity in pathways involved in cytotoxicity, drug resistance, cell cycle, and other biological processes altered in specific cancer types and diseases, might lead to better modeling of compound activity [70]. Similarly, using biologically meaningful targets to construct QAFFP might provide a better stratification between active and inactive compounds in bioactivity space, and hence enable the generation of models with higher predictive power [33, 70, 71]. Another important aspect to consider is that the sensitivity of some cancer cell lines to certain chemicals with well-defined mechanisms of action depends on the modulation of one or few proteins or pathways [71–76]. In such cases, using compound activity on a small set of assays as descriptors, and univariate or low-dimensional models might be sufficient to accurately model drug response [33, 77]. Here, instead of focusing on specific targets associated to the activity of few compounds, we have considered a data-driven approach that can be applicable to (potentially) any compound irrespective of its mechanism of action.

## Conclusions

This study complements the accompanying paper [Skuta et al.], where the performance of QAFFP for similarity searching, compound classification and scaffold hopping is reported. Here, we have performed a comprehensive assessment of the performance of regression models trained on QSAR-derived affinity fingerprints (QAFFP). QAFFP enabled the generation of highly predictive models, with RMSE values in the ~ 0.6–0.9 pIC_50_ units range, which is comparable to the predictive power obtained using Morgan2 fingerprints and physicochemical descriptors, as well as to the uncertainty of heterogeneous IC_50_ data in ChEMBL. This level of performance is in line with the high predictive power obtained with QAFFP in similarity searching, compound classification, and scaffold hopping tasks [Skuta et al.]. Notably, QAFFP calculated using base models showing high and moderate performance were more predictive than those trained on QAFFP generated using highly predictive models alone, and likely the increased ability to describe variance in the mapping from chemical to biological space seems to be the cause of this behaviour. To further evaluate the practical utility of QAFFP, future studies will be needed to challenge them in more complex scenarios, including the modeling of the synergistic or antagonistic effect of compound combinations [79–82], and to test whether the integration of QAFFP and cell line profiling data sets (e.g., basal gene expression profiles, or changes in gene expression induced upon compound administration) improves drug sensitivity modeling.

## Supplementary information

**Additional file 1: Figure S1.** Distribution of pIC_50_ values for all data sets modelled in this study. **Figure S2.** Model performance on the test set as a function of the number of trees in the Random Forest models for a random selection of 18 data sets. **Figure S3.** R^2^_test_ values calculated with models trained on each of the 11 descriptor types considered across the 43 data sets modelled in this study (related to Fig. 4). We trained 50 models for each combination of descriptor type and data set, each time holding a different subset of the data as test set. **Figure S4.** Y-scrambling experiments. R^2^_test_ values calculated for models trained after shuffling the response variable are shown. **Figure S5.** RMSE_test_ values as a function of the fraction of the training data used as test set for all data sets. **Figure S6.** Mean variable importance +/− standard deviation averaged across 50 replicates. Only the top 20 descriptors are shown for each data set. **Figure S7.** Examples of compounds that were predicted with higher error by models trained on Morgan2 fingerprints than by models trained on rv-QAFFP 440. The predictions were calculated on the test set across 50 replicates. The mean and the standard deviation across these 50 replicates are shown. The data set is indicated below the compounds ChEMBL IDs. **Figure S8.** Predicted pIC_50_ values using models trained on the fingerprint type indicated in x-axis, against the predicted pIC_50_ values calculated using models trained using the fingerprint type indicated in the y-axis. The plot shows the predictions for 50 replicates for data set A2780. Similar results were obtained for the other data sets. Overall, it can be seen that the predictions generated by models trained using Morgan2 fingerprints and the rv-QAFFP 440 versions considered are highly correlated across the entire bioactivity range modelled.

**Additional file 2: Table S1** Value and significance for the coefficients of the linear model used to assess the performance of the 11 descriptor types considered. The code and the 43 data sets used in this study are provided at https://github.com/isidroc/QAFFP_regression.
