# Supplementary material for: QSAR-derived affinity fingerprints (part 2): modeling performance for potency prediction
Source: J Cheminform. 2020 Jun 5;12:41. doi: 10.1186/s13321-020-00444-5 (PMC7339533; doi:10.1186/s13321-020-00444-5)

Supplementary Figure 1

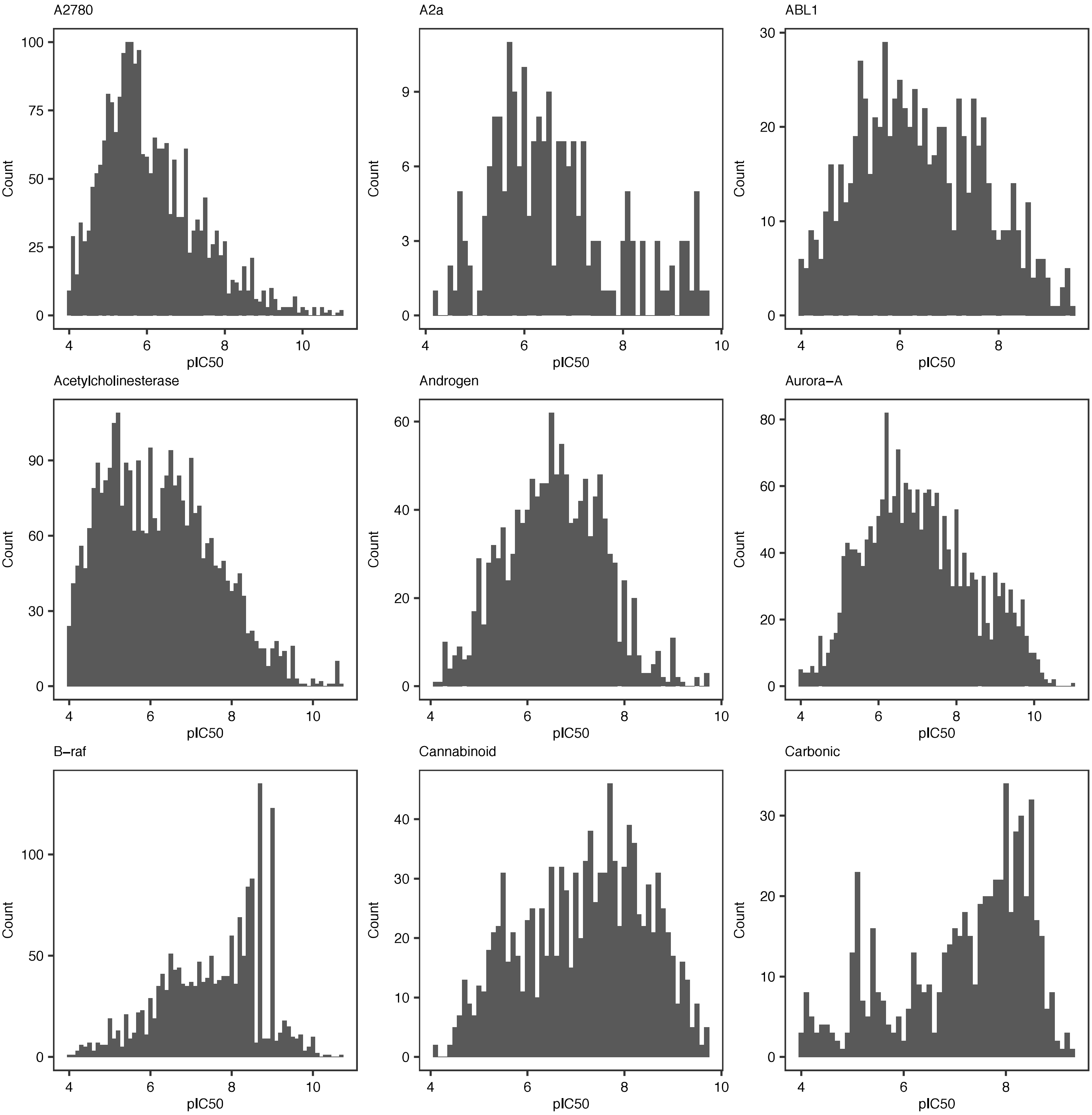

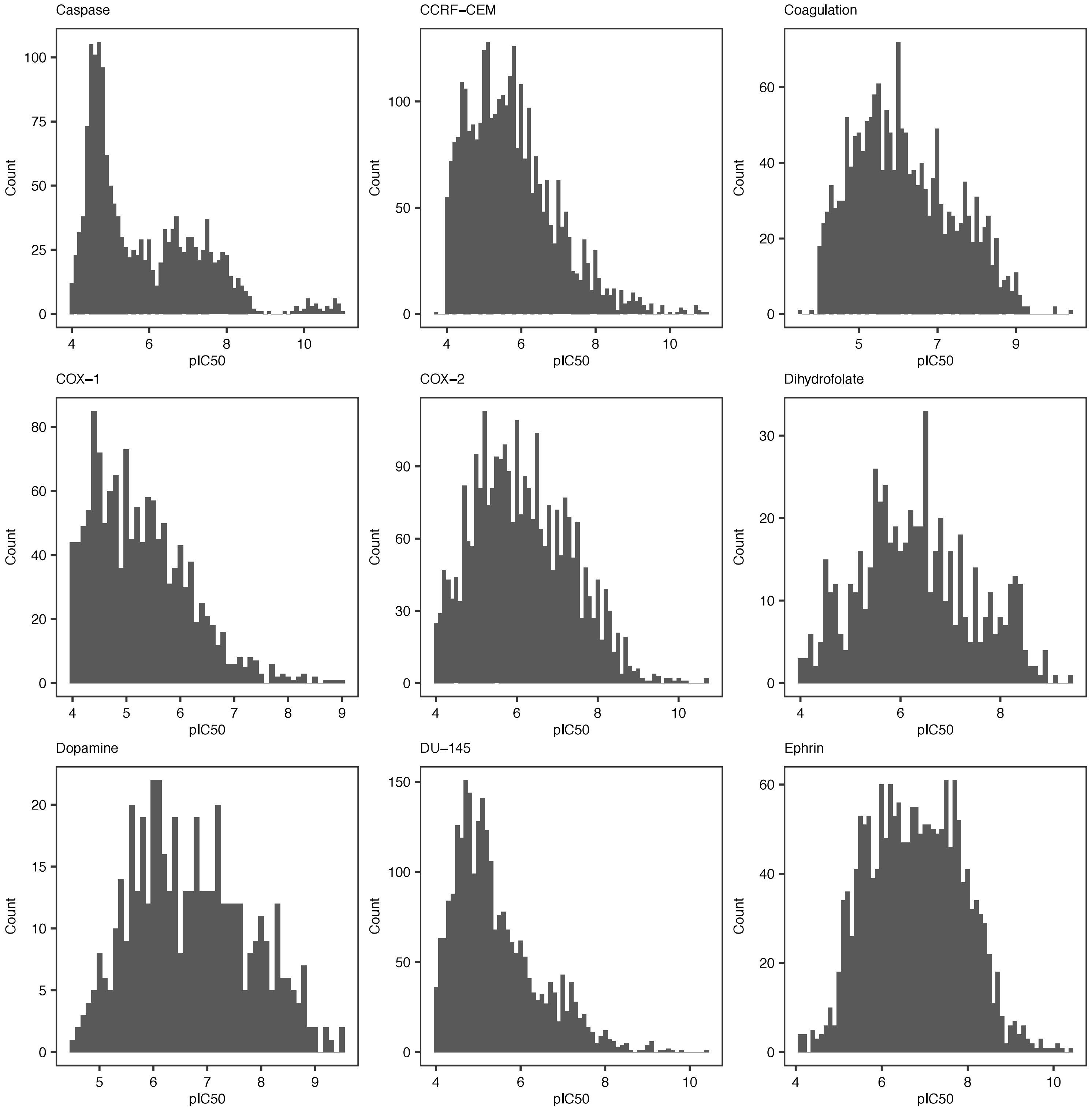
Caspase
CCRF−CEM
Coagulation
COX−1
COX−2
Dihydrofolate
Dopamine
DU−145
Ephrin
Count
pIC50

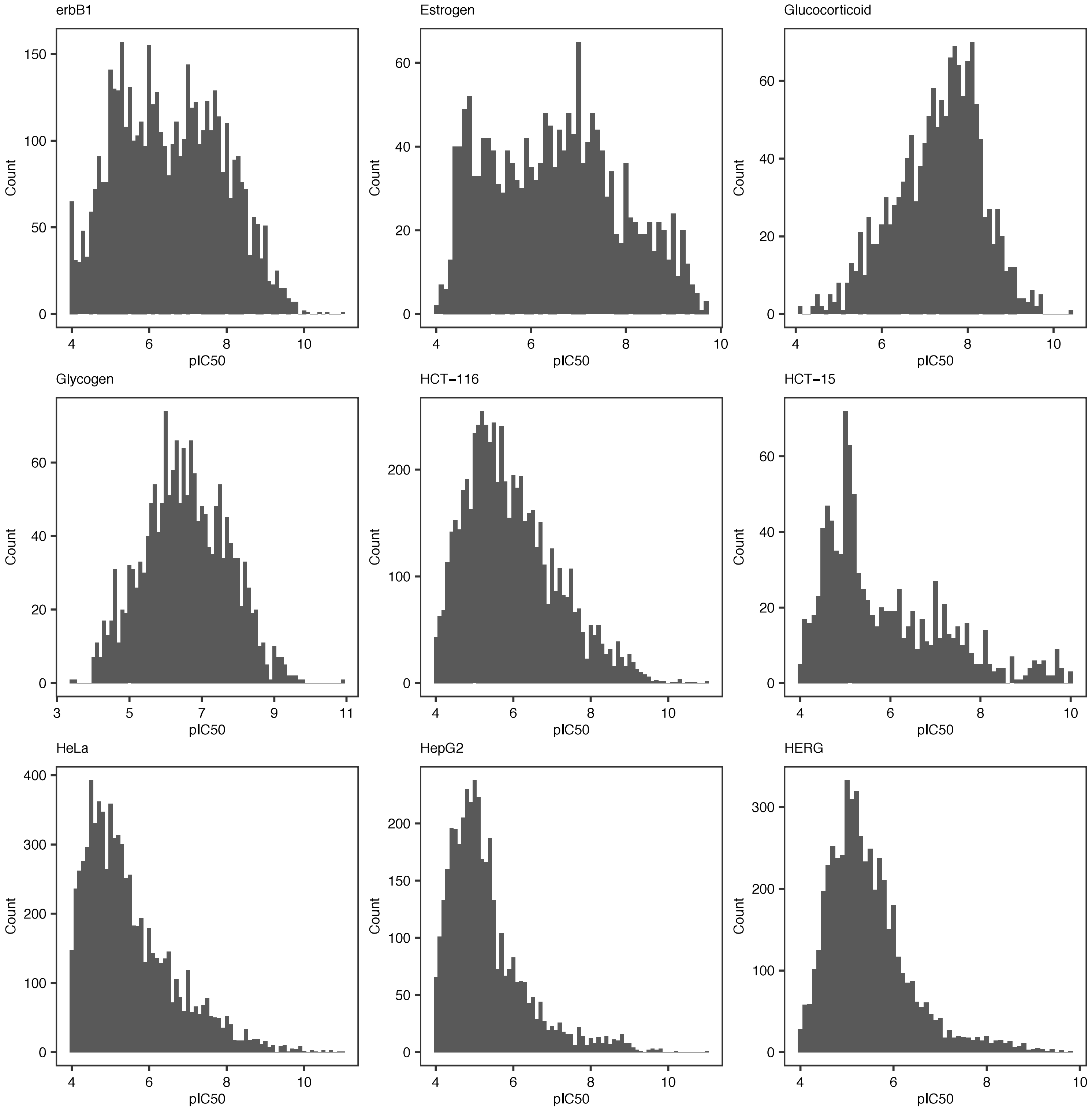
erbB1
Estrogen
Glucocorticoid
Glycogen
HCT−116
HCT−15
HeLa
HepG2
HERG
Count
pIC50

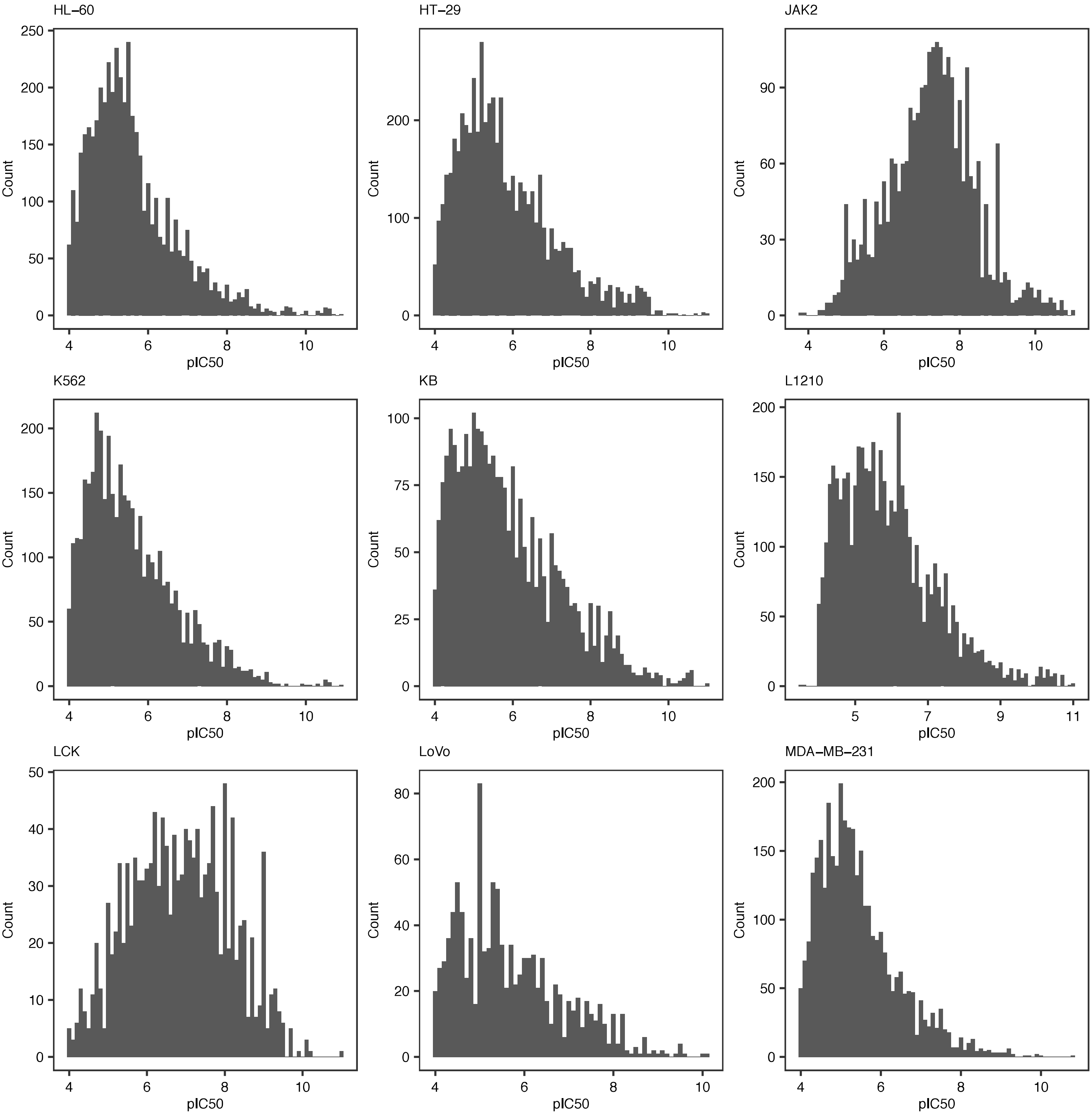
HL−60
HT−29
JAK2
K562
KB
L1210
LCK
LoVo
MDA−MB−231
Count
pIC50

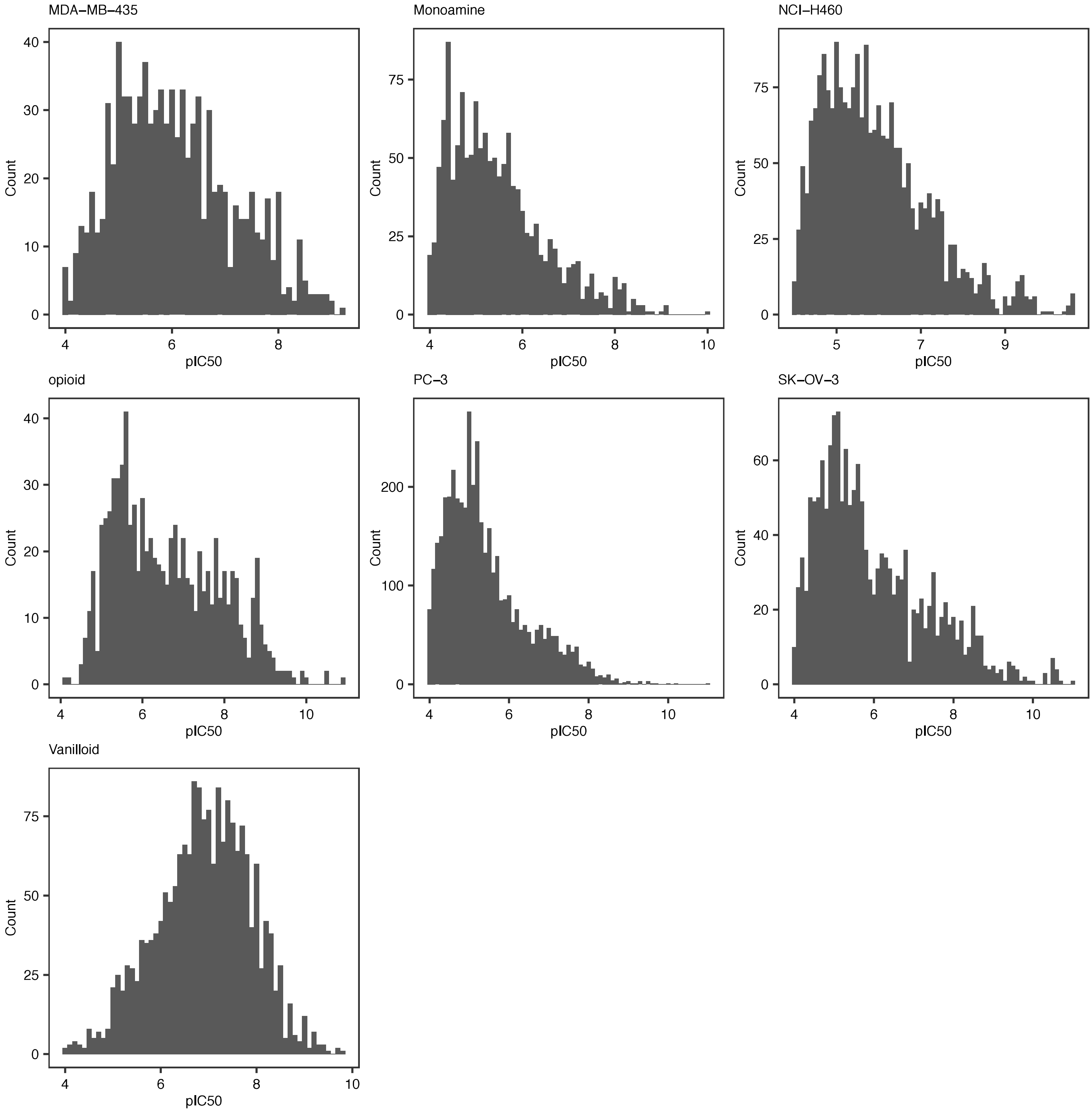
MDA−MB−435
Monoamine
NCI−H460
opioid
PC−3
SK−OV−3
Vanilloid
Count
pIC50

Supplementary Figure 2

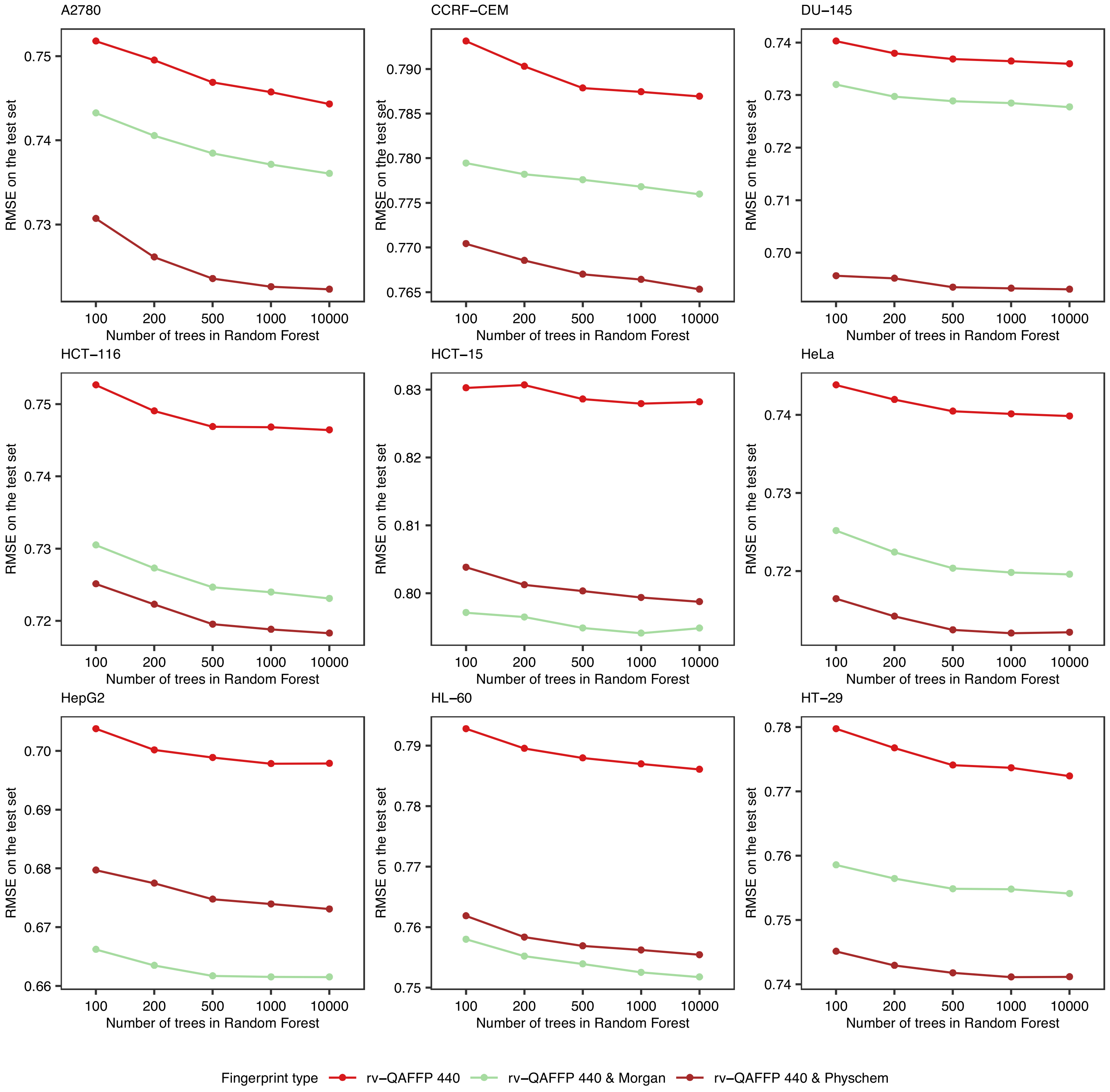

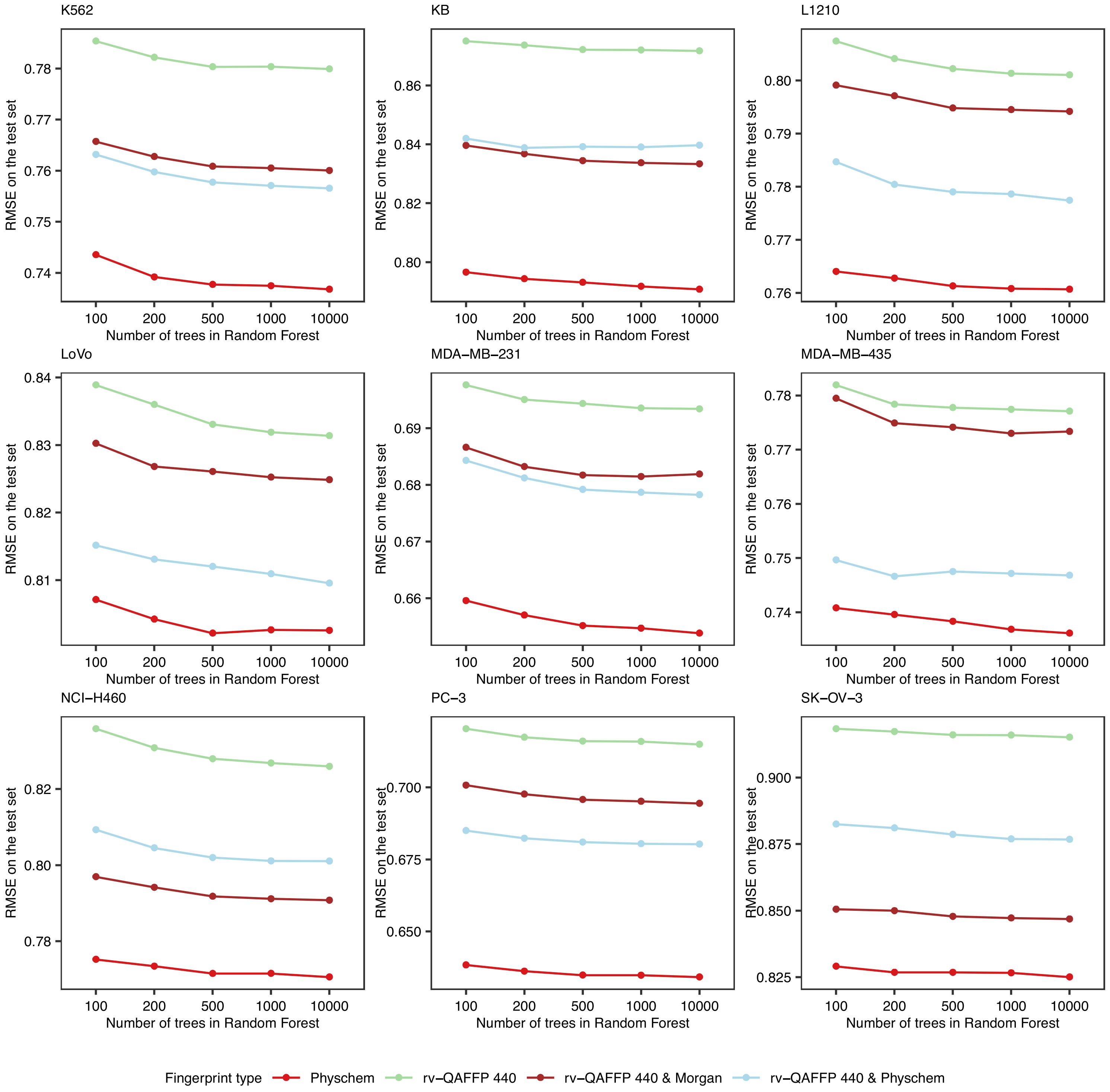
K562
RMSE on the test set
0.78
0.77
0.76
0.75
0.74
KB
0.86
0.84
0.82
0.80
L1210
0.80
0.79
0.78
0.77
0.76
LoVo
0.84
0.83
0.82
0.81
MDA–MB–231
0.69
0.68
0.67
0.66
MDA–MB–435
0.78
0.77
0.76
0.75
0.74
NCI–H460
0.82
0.80
0.78
PC–3
0.700
0.675
0.650
SK–OV–3
0.900
0.875
0.850
0.825
100
200
500
1000
10000
Number of trees in Random Forest
Fingerprint type
Physchem
rv–QAFFP 440
rv–QAFFP 440 & Morgan
rv–QAFFP 440 & Physchem

Supplementary Figure 3

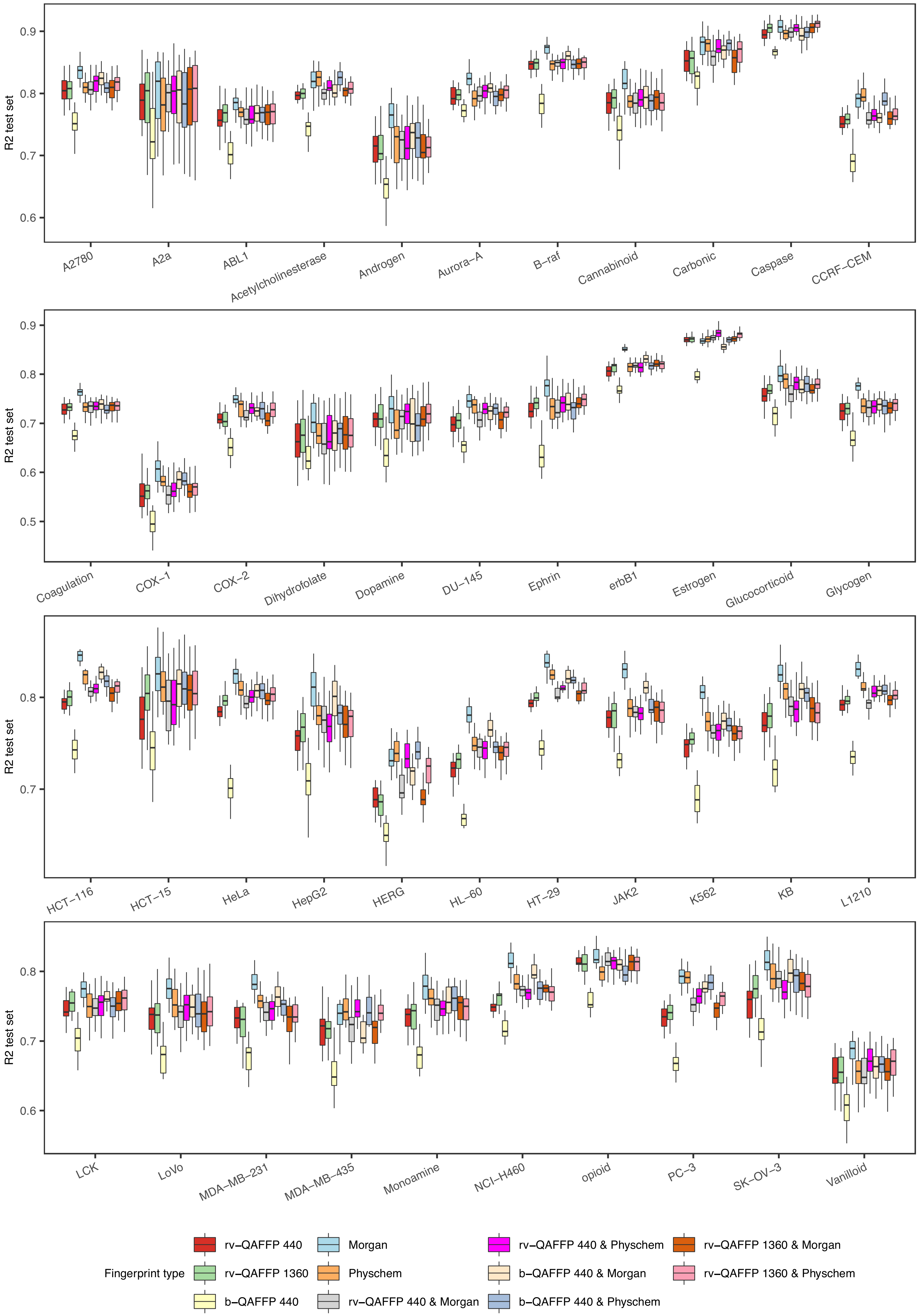

Supplementary Figure 4

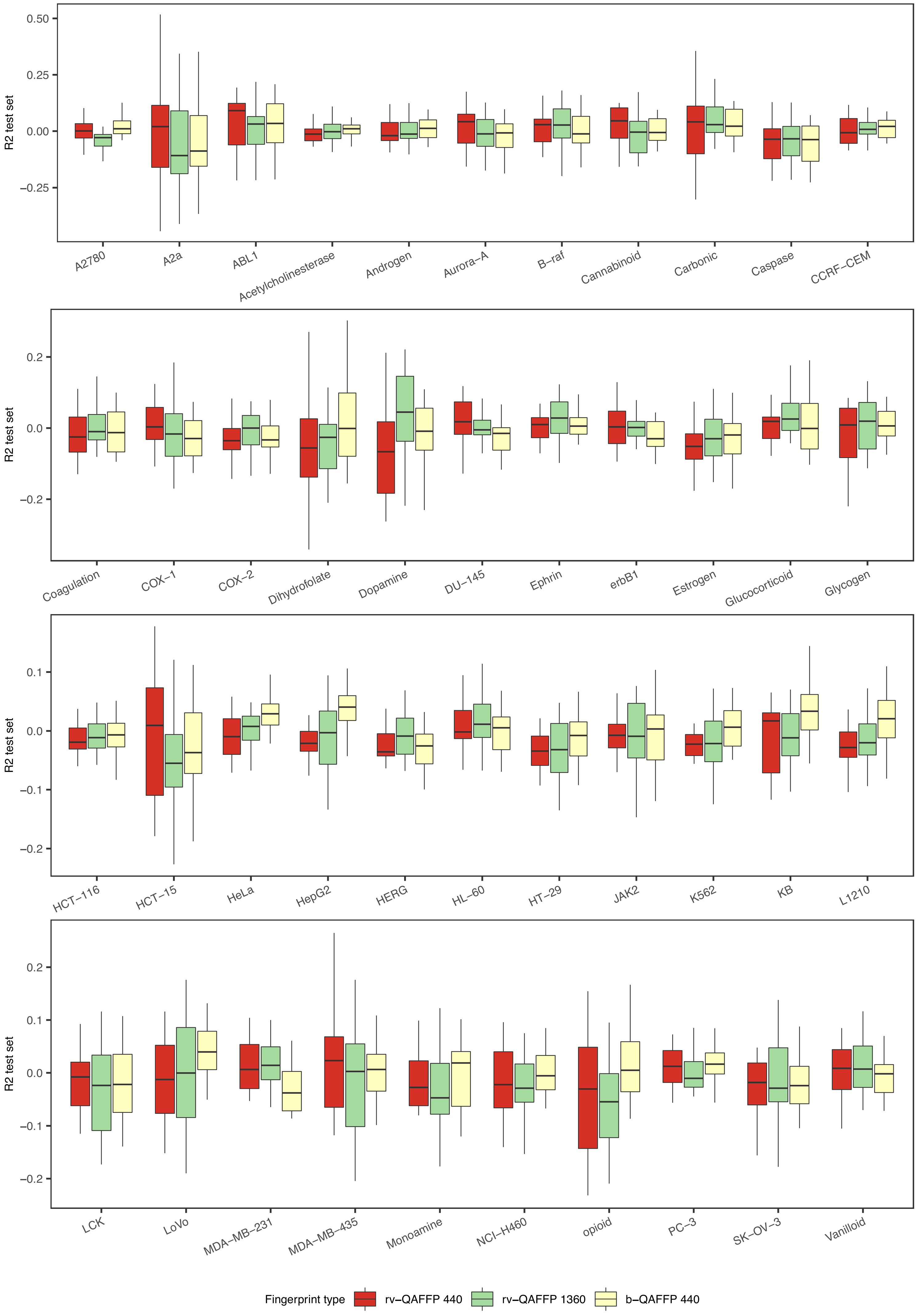

Supplementary Figure 5

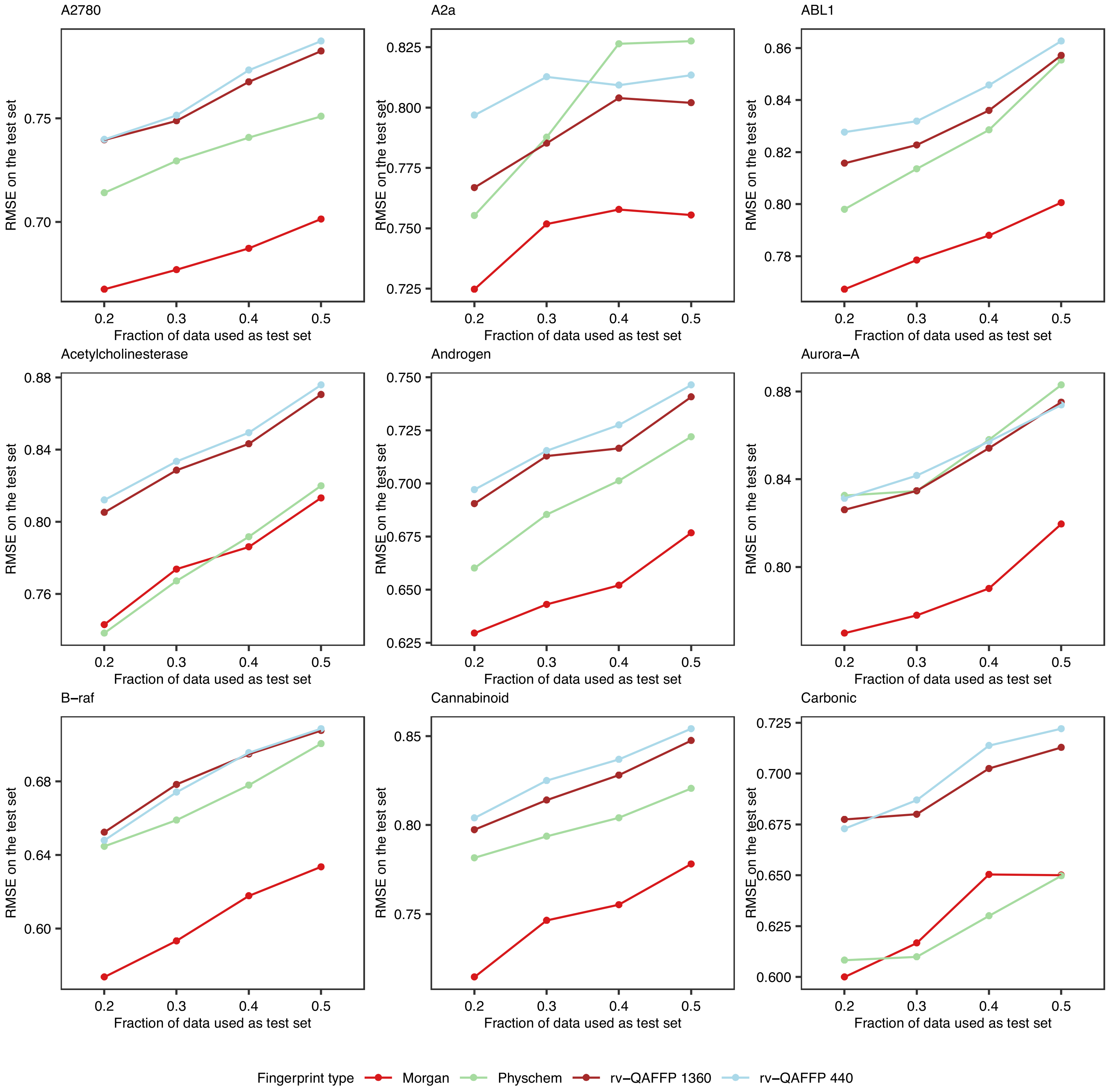

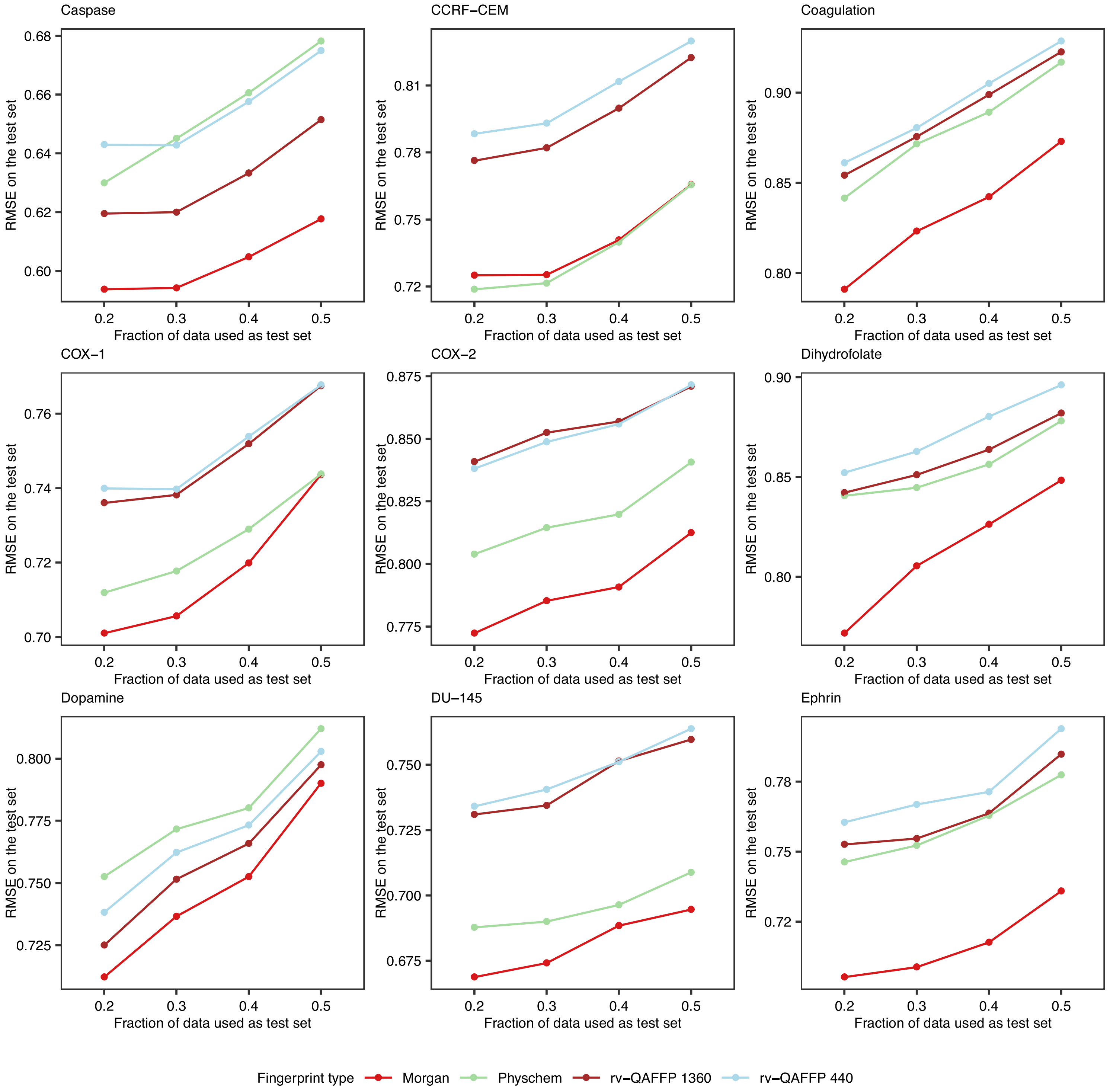

Caspase
CCRF−CEM
Coagulation
COX−1
COX−2
Dihydrofolate
Dopamine
DU−145
Ephrin
RMSE on the test set
Fraction of data used as test set
Fingerprint type
Morgan
Physchem
rv−QAFFP 1360
rv−QAFFP 440

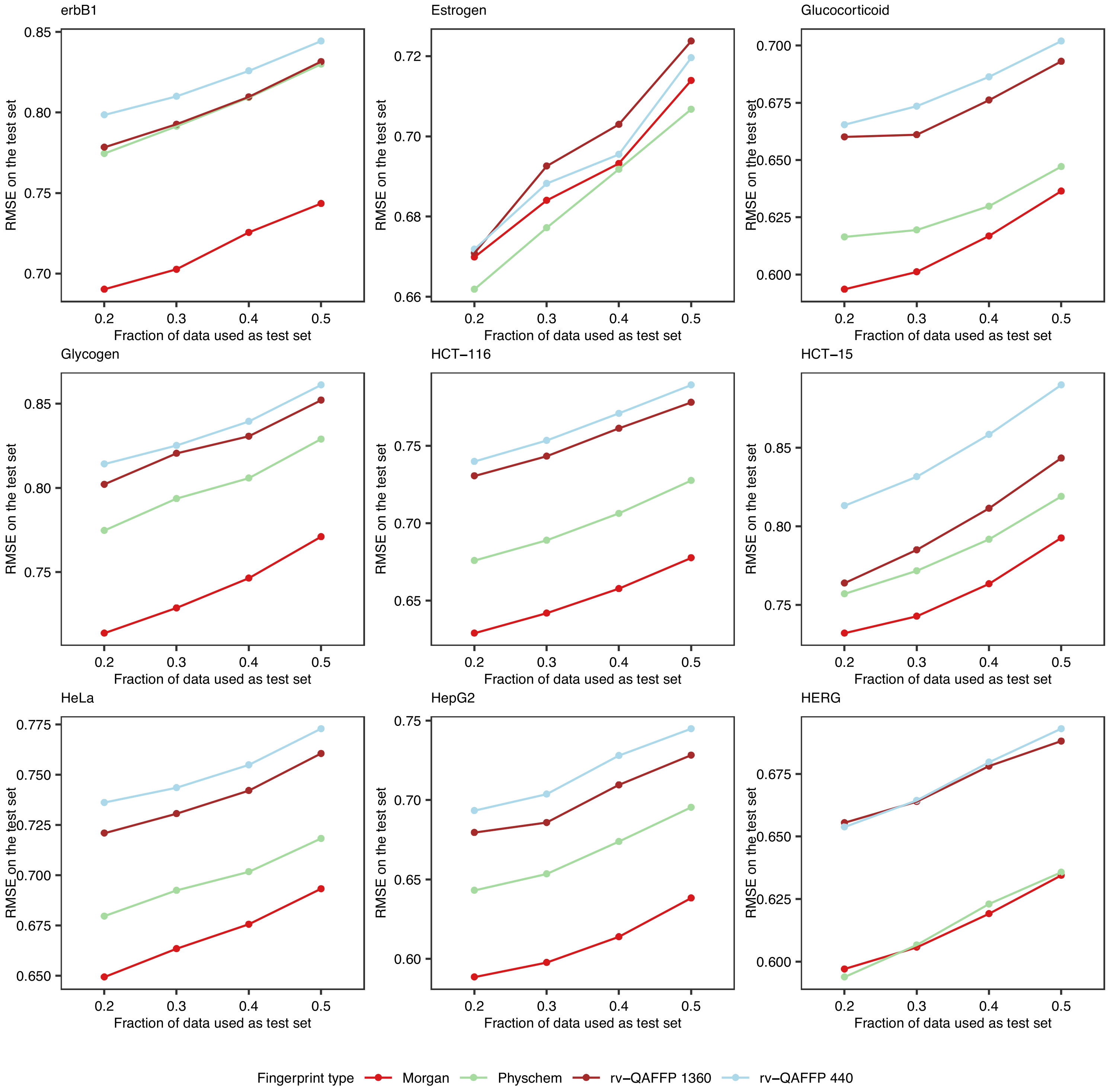
erbB1
Estrogen
Glucocorticoid
Glycogen
HCT−116
HCT−15
HeLa
HepG2
HERG
RMSE on the test set
Fraction of data used as test set
Fingerprint type
Morgan
Physchem
rv−QAFFP 1360
rv−QAFFP 440

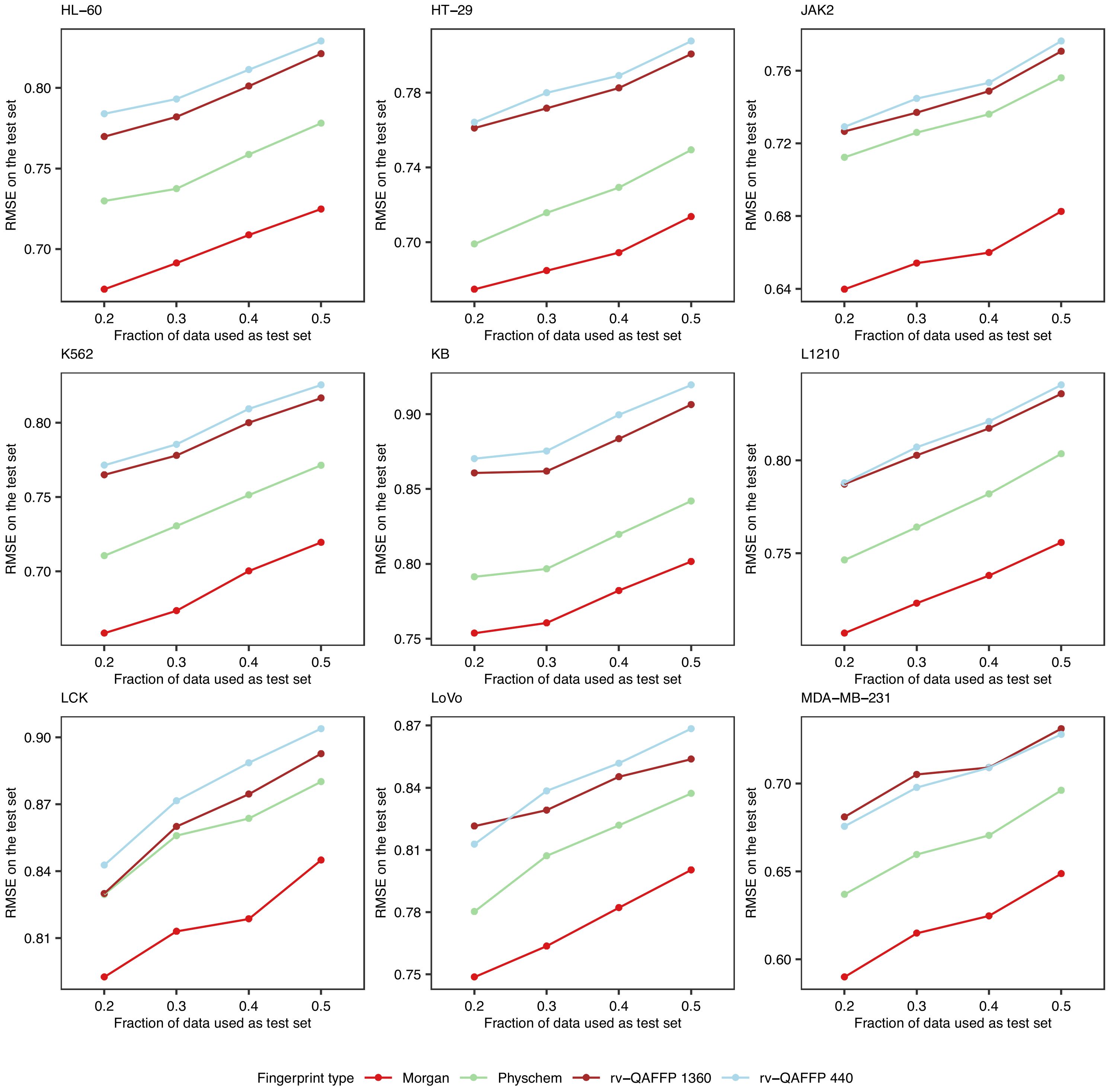

HL−60
HT−29
JAK2
K562
KB
L1210
LCK
LoVo
MDA−MB−231
RMSE on the test set
Fraction of data used as test set
Fingerprint type
Morgan
Physchem
rv−QAFFP 1360
rv−QAFFP 440

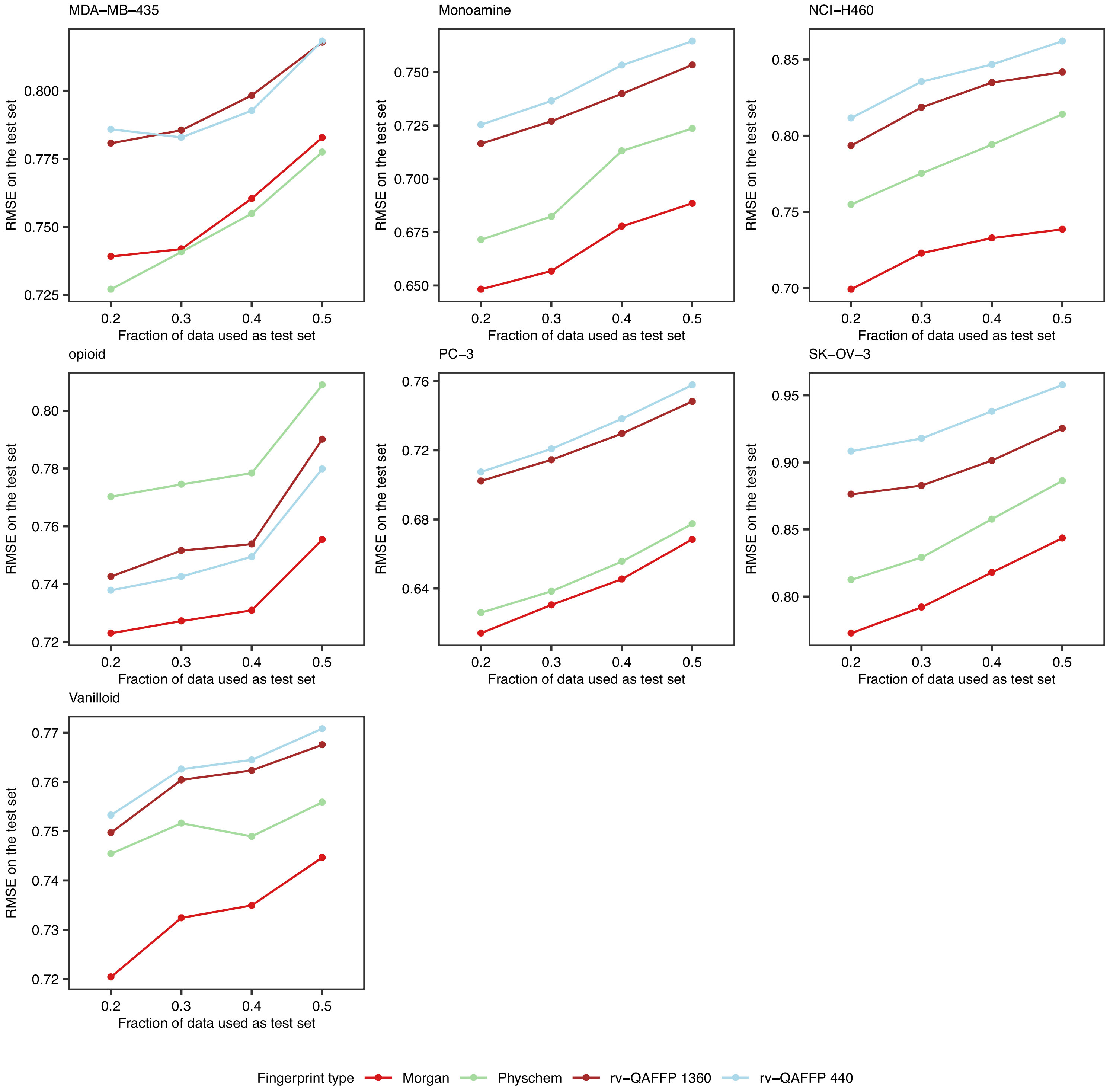

MDA–MB–435
Monoamine
NCI–H460
opioid
PC–3
SK–OV–3
Vanilloid
RMSE on the test set
Fraction of data used as test set
Fingerprint type
Morgan
Physchem
rv–QAFFP 1360
rv–QAFFP 440

Supplementary Figure 6

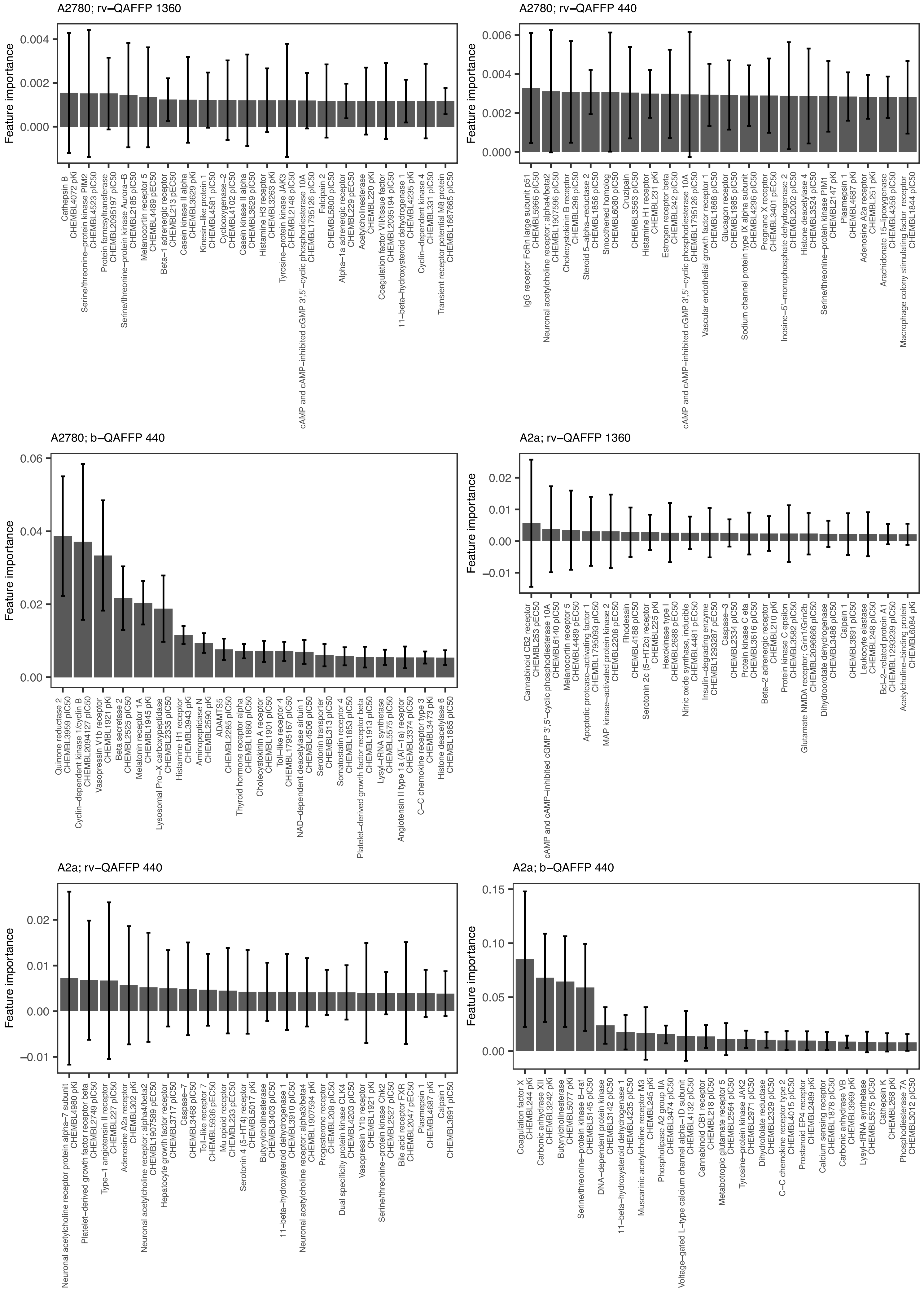

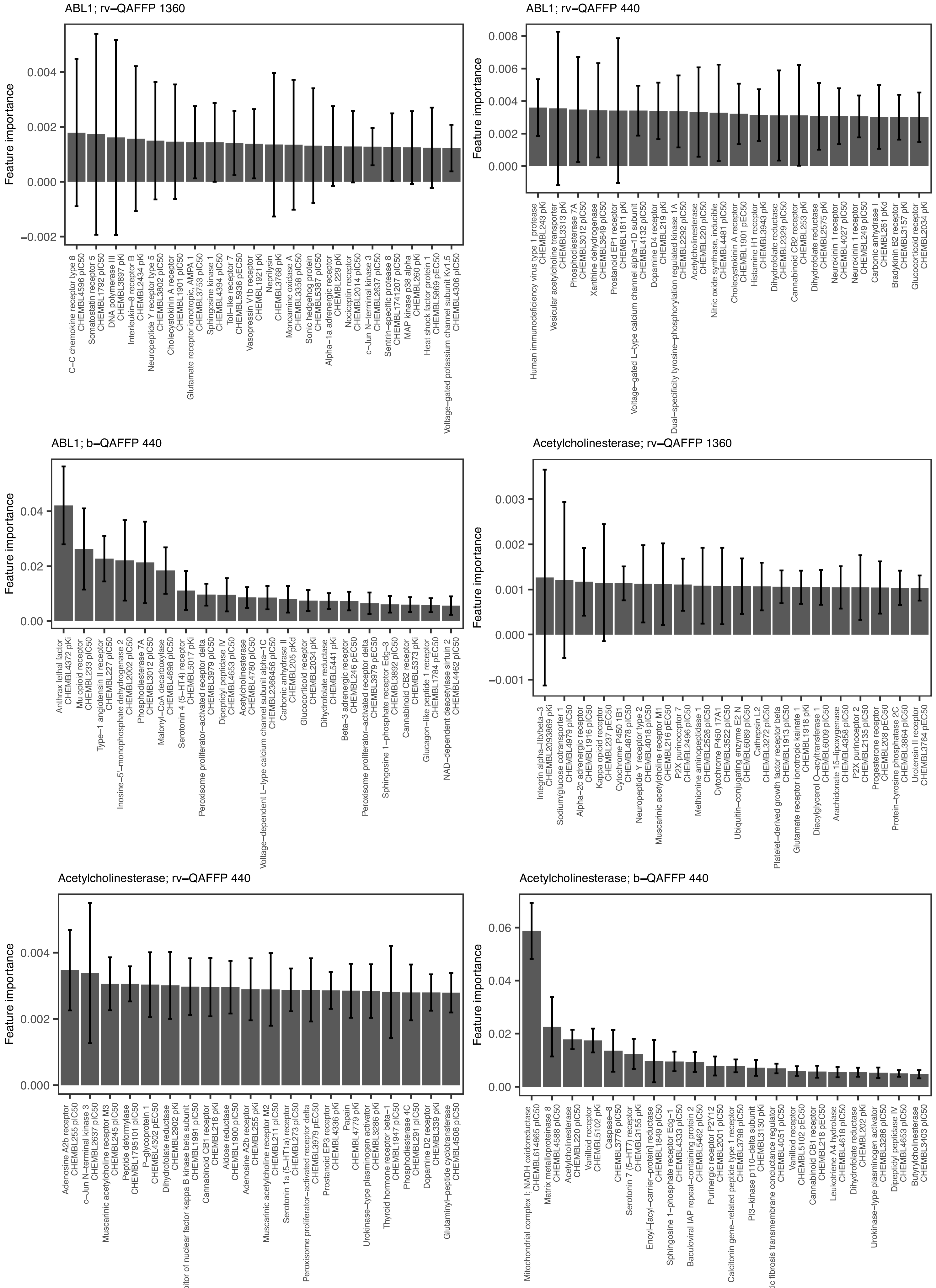

ABL1; rv−QAFFP 1360
Feature importance
0.004
0.002
0.000
−0.002
C−C chemokine receptor type 8 CHEMBL4596 pIC50
Somatostatin receptor 5 CHEMBL1792 pIC50
DNA polymerase III CHEMBL3897 pKi
Interleukin−8 receptor B CHEMBL2434 pIC50
Neuropeptide Y receptor type 5 CHEMBL3802 pIC50
Cholecystokinin A receptor CHEMBL1901 pIC50
Glutamate receptor ionotropic, AMPA 1 CHEMBL3753 pIC50
Sphingosine kinase 1 CHEMBL4394 pIC50
Toll−like receptor 7 CHEMBL5936 pEC50
Vasopressin V1b receptor CHEMBL1921 pKi
Neprilysin CHEMBL3768 pKi
Monoamine oxidase A CHEMBL3358 pIC50
Sonic hedgehog protein CHEMBL5387 pIC50
Alpha−1a adrenergic receptor CHEMBL229 pKi
Nociceptin receptor CHEMBL2014 pIC50
c−Jun N−terminal kinase 3 CHEMBL2637 pIC50
Sentrin−specific protease 8 CHEMBL1741207 pIC50
MAP kinase p38 alpha CHEMBL260 pKi
Heat shock factor protein 1 CHEMBL5869 pEC50
Voltage−gated potassium channel subunit Kv1.5 CHEMBL4306 pIC50
ABL1; rv−QAFFP 440
Feature importance
0.008
0.006
0.004
0.002
0.000
Human immunodeficiency virus type 1 protease CHEMBL243 pKi
Vesicular acetylcholine transporter CHEMBL3313 pKi
Phosphodiesterase 7A CHEMBL3012 pIC50
Xanthine dehydrogenase CHEMBL3649 pIC50
Prostanoid EP1 receptor CHEMBL1811 pKi
Voltage−gated L−type calcium channel alpha−1D subunit CHEMBL4132 pIC50
Dopamine D4 receptor CHEMBL219 pKi
Dual−specificity tyrosine−phosphorylation regulated kinase 1A CHEMBL2292 pIC50
Acetylcholinesterase CHEMBL220 pIC50
Nitric oxide synthase, inducible CHEMBL4481 pIC50
Cholecystokinin A receptor CHEMBL1901 pEC50
Histamine H1 receptor CHEMBL3943 pKi
Dihydrofolate reductase CHEMBL2329 pIC50
Cannabinoid CB2 receptor CHEMBL253 pKi
Dihydrofolate reductase CHEMBL2575 pKi
Neurokinin 1 receptor CHEMBL4027 pIC50
Neurokinin 1 receptor CHEMBL249 pIC50
Carbonic anhydrase I CHEMBL261 pKd
Bradykinin B2 receptor CHEMBL3157 pKi
Glucocorticoid receptor CHEMBL2034 pKi
ABL1; b−QAFFP 440
Feature importance
0.04
0.02
0.00
Anthrax lethal factor CHEMBL4372 pKi
Mu opioid receptor CHEMBL233 pIC50
Type−1 angiotensin II receptor CHEMBL227 pIC50
Inosine−5'−monophosphate dehydrogenase 2 CHEMBL2002 pIC50
Phosphodiesterase 7A CHEMBL3012 pIC50
Malonyl−CoA decarboxylase CHEMBL4698 pIC50
Serotonin 4 (5−HT4) receptor CHEMBL5017 pKi
Peroxisome proliferator−activated receptor delta CHEMBL3979 pIC50
Dipeptidyl peptidase IV CHEMBL4653 pIC50
Acetylcholinesterase CHEMBL4780 pIC50
Voltage−dependent L−type calcium channel subunit alpha−1C CHEMBL2366456 pIC50
Carbonic anhydrase II CHEMBL205 pKd
Glucocorticoid receptor CHEMBL2034 pKi
Dihydrofolate reductase CHEMBL5441 pKi
Beta−3 adrenergic receptor CHEMBL246 pEC50
Peroxisome proliferator−activated receptor delta CHEMBL3979 pEC50
Sphingosine 1−phosphate receptor Edg−3 CHEMBL3892 pIC50
Cannabinoid CB2 receptor CHEMBL5373 pKi
Glucagon−like peptide 1 receptor CHEMBL1784 pEC50
NAD−dependent deacetylase sirtuin 2 CHEMBL4462 pIC50
Acetylcholinesterase; rv−QAFFP 1360
Feature importance
0.003
0.002
0.001
0.000
−0.001
Integrin alpha−IIb/beta−3 CHEMBL2093869 pKi
Sodium/glucose cotransporter 1 CHEMBL4979 pIC50
Alpha−2c adrenergic receptor CHEMBL1916 pIC50
Kappa opioid receptor CHEMBL237 pEC50
Cytochrome P450 1B1 CHEMBL4878 pIC50
Neuropeptide Y receptor type 2 CHEMBL4018 pIC50
Muscarinic acetylcholine receptor M1 CHEMBL216 pEC50
P2X purinoceptor 7 CHEMBL2496 pIC50
Methionine aminopeptidase 1 CHEMBL2526 pIC50
Cytochrome P450 17A1 CHEMBL3522 pIC50
Ubiquitin−conjugating enzyme E2 N CHEMBL6089 pIC50
Cathepsin L2 CHEMBL3272 pIC50
Platelet−derived growth factor receptor beta CHEMBL1913 pIC50
Glutamate receptor ionotropic kainate 1 CHEMBL1918 pKi
Diacylglycerol O−acyltransferase 1 CHEMBL6009 pIC50
Arachidonate 15−lipoxygenase CHEMBL4358 pIC50
P2X purinoceptor 2 CHEMBL2135 pIC50
Progesterone receptor CHEMBL208 pEC50
Protein−tyrosine phosphatase 2C CHEMBL3864 pIC50
Urotensin II receptor CHEMBL3764 pEC50
Acetylcholinesterase; rv−QAFFP 440
Feature importance
0.004
0.002
0.000
Adenosine A2b receptor CHEMBL255 pIC50
c−Jun N−terminal kinase 3 CHEMBL2637 pIC50
Muscarinic acetylcholine receptor M3 CHEMBL245 pIC50
Peptide deformylase CHEMBL1795101 pIC50
P−glycoprotein 1 CHEMBL4302 pEC50
Dihydrofolate reductase CHEMBL2902 pKi
Inhibitor of nuclear factor kappa B kinase beta subunit CHEMBL1991 pIC50
Cannabinoid CB1 receptor CHEMBL218 pKi
Aldose reductase CHEMBL1900 pIC50
Adenosine A2b receptor CHEMBL255 pKi
Muscarinic acetylcholine receptor M2 CHEMBL211 pIC50
Serotonin 1a (5−HT1a) receptor CHEMBL273 pIC50
Peroxisome proliferator−activated receptor delta CHEMBL3979 pEC50
Prostanoid EP3 receptor CHEMBL4336 pIC50
Papain CHEMBL4779 pKi
Urokinase−type plasminogen activator CHEMBL3286 pIC50
Thyroid hormone receptor beta−1 CHEMBL1947 pIC50
Phosphodiesterase 4C CHEMBL291 pIC50
Dopamine D2 receptor CHEMBL339 pKi
Glutaminyl−peptide cyclotransferase CHEMBL4508 pIC50
Acetylcholinesterase; b−QAFFP 440
Feature importance
0.06
0.04
0.02
0.00
Mitochondrial complex I: NADH oxidoreductase CHEMBL614865 pIC50
Matrix metalloproteinase 8 CHEMBL4588 pIC50
Acetylcholinesterase CHEMBL220 pIC50
Vanilloid receptor CHEMBL5102 pKi
Caspase−8 CHEMBL3776 pIC50
Serotonin 7 (5−HT7) receptor CHEMBL3155 pKi
Enoyl−[acyl−carrier−protein] reductase CHEMBL1849 pIC50
Sphingosine 1−phosphate receptor Edg−1 CHEMBL4333 pIC50
Baculoviral IAP repeat−containing protein 2 CHEMBL5462 pIC50
Purinergic receptor P2Y12 CHEMBL2001 pIC50
Calcitonin gene−related peptide type 1 receptor CHEMBL3798 pIC50
PI3−kinase p110−delta subunit CHEMBL3130 pKi
Cystic fibrosis transmembrane conductance regulator CHEMBL4051 pIC50
Vanilloid receptor CHEMBL5102 pEC50
Cannabinoid CB1 receptor CHEMBL218 pEC50
Leukotriene A4 hydrolase CHEMBL4618 pIC50
Dihydrofolate reductase CHEMBL202 pKi
Urokinase−type plasminogen activator CHEMBL3286 pIC50
Dipeptidyl peptidase IV CHEMBL4653 pIC50
Butyrylcholinesterase CHEMBL3403 pIC50

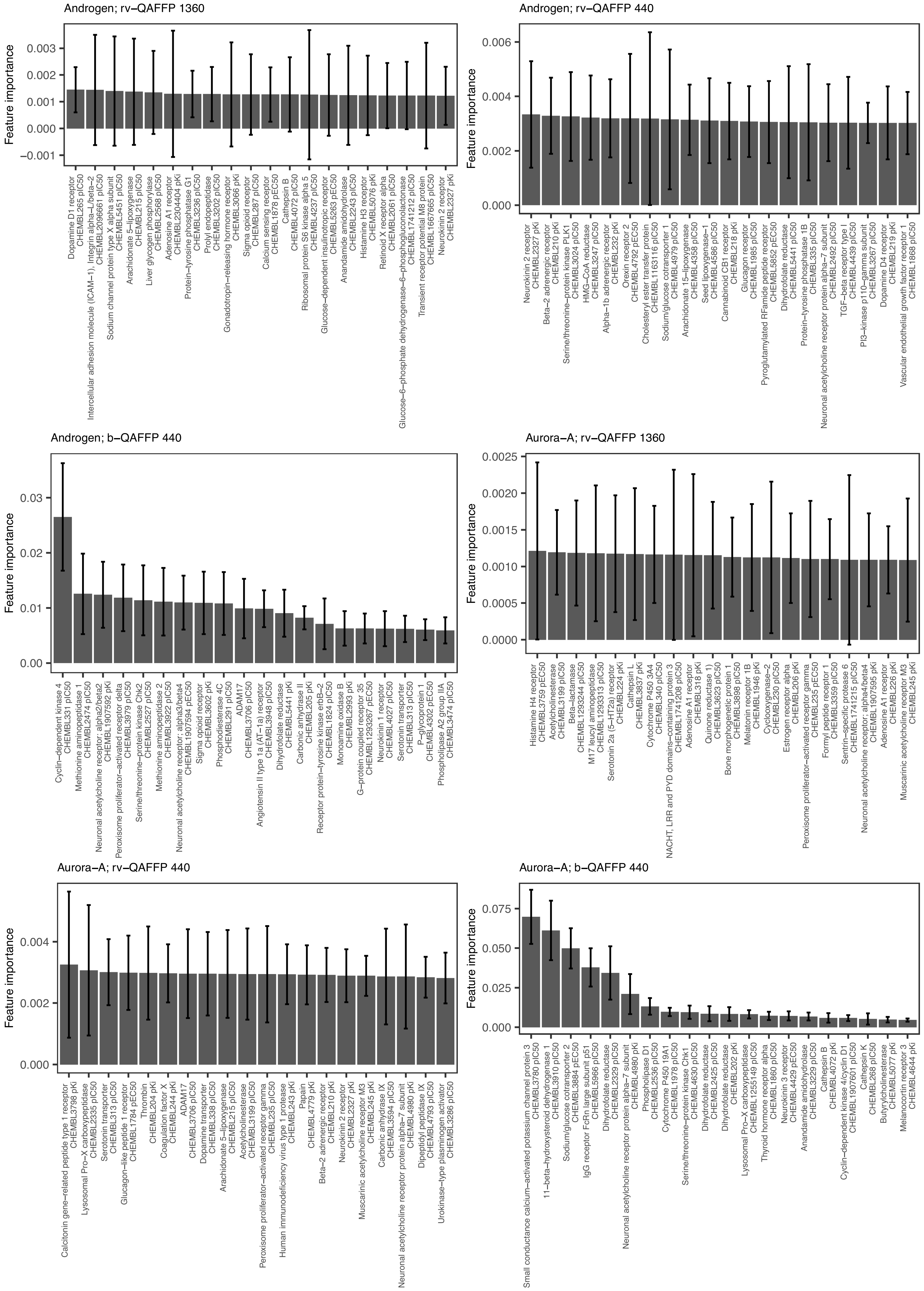
Androgen; rv–QAFFP 1360
Feature importance
0.003
0.002
0.001
0.000
−0.001
Dopamine D1 receptor CHEMBL265 pIC50
Intercellular adhesion molecule (ICAM−1), Integrin alpha−L/beta−2 CHEMBL2096661 pIC50
Sodium channel protein type X alpha subunit CHEMBL5451 pIC50
Arachidonate 5−lipoxygenase CHEMBL215 pIC50
Liver glycogen phosphorylase CHEMBL2568 pIC50
Adenosine A1 receptor CHEMBL2304404 pKi
Protein−tyrosine phosphatase G1 CHEMBL3236 pIC50
Prolyl endopeptidase CHEMBL3202 pIC50
Gonadotropin−releasing hormone receptor CHEMBL3066 pKi
Sigma opioid receptor CHEMBL287 pIC50
Calcium sensing receptor CHEMBL1878 pEC50
Cathepsin B CHEMBL4072 pIC50
Ribosomal protein S6 kinase alpha 5 CHEMBL4237 pIC50
Glucose−dependent insulinotropic receptor CHEMBL5263 pEC50
Anandamide amidohydrolase CHEMBL2243 pIC50
Histamine H3 receptor CHEMBL5076 pKi
Retinoid X receptor alpha CHEMBL2061 pIC50
Glucose−6−phosphate dehydrogenase−6−phosphogluconolactonase CHEMBL1741212 pIC50
Transient receptor potential M8 protein CHEMBL1667665 pIC50
Neurokinin 2 receptor CHEMBL2327 pKi
Androgen; rv–QAFFP 440
Feature importance
0.006
0.004
0.002
0.000
Neurokinin 2 receptor CHEMBL2327 pKi
Beta−2 adrenergic receptor CHEMBL210 pKi
Serine/threonine−protein kinase PLK1 CHEMBL3024 pIC50
HMG−CoA reductase CHEMBL3247 pIC50
Alpha−1b adrenergic receptor CHEMBL232 pKi
Orexin receptor 2 CHEMBL4792 pEC50
Cholesteryl ester transfer protein CHEMBL1163116 pIC50
Sodium/glucose cotransporter 1 CHEMBL4979 pIC50
Arachidonate 15−lipoxygenase CHEMBL4358 pIC50
Seed lipoxygenase−1 CHEMBL4586 pIC50
Cannabinoid CB1 receptor CHEMBL218 pKi
Glucagon receptor CHEMBL1985 pIC50
Pyroglutamylated RFamide peptide receptor CHEMBL5852 pEC50
Dihydrofolate reductase CHEMBL5441 pIC50
Protein−tyrosine phosphatase 1B CHEMBL335 pIC50
Neuronal acetylcholine receptor protein alpha−7 subunit CHEMBL2492 pIC50
TGF−beta receptor type I CHEMBL4439 pIC50
PI3−kinase p110−gamma subunit CHEMBL3267 pIC50
Dopamine D4 receptor CHEMBL219 pKi
Vascular endothelial growth factor receptor 1 CHEMBL1868 pIC50
Androgen; b–QAFFP 440
Feature importance
0.03
0.02
0.01
0.00
Cyclin−dependent kinase 4 CHEMBL331 pIC50
Methionine aminopeptidase 1 CHEMBL2474 pIC50
Neuronal acetylcholine receptor; alpha2/beta2 CHEMBL1907592 pKi
Peroxisome proliferator−activated receptor delta CHEMBL3979 pIC50
Serine/threonine−protein kinase Chk2 CHEMBL2527 pIC50
Methionine aminopeptidase 2 CHEMBL3922 pIC50
Neuronal acetylcholine receptor; alpha3/beta4 CHEMBL1907594 pEC50
Sigma opioid receptor CHEMBL3602 pKi
Phosphodiesterase 4C CHEMBL291 pIC50
ADAM17 CHEMBL3706 pIC50
Angiotensin II type 1a (AT−1a) receptor CHEMBL3948 pIC50
Dihydrofolate reductase CHEMBL5441 pKi
Carbonic anhydrase II CHEMBL205 pIC50
Receptor protein−tyrosine kinase erbB−2 CHEMBL1824 pIC50
Monoamine oxidase B CHEMBL2993 pKi
G−protein coupled receptor 35 CHEMBL1293267 pEC50
Neurokinin 1 receptor CHEMBL4027 pIC50
Serotonin transporter CHEMBL313 pIC50
P−glycoprotein 1 CHEMBL4302 pEC50
Phospholipase A2 group IIA CHEMBL3474 pIC50
Aurora−A; rv–QAFFP 1360
Feature importance
0.0025
0.0020
0.0015
0.0010
0.0005
0.0000
Histamine H4 receptor CHEMBL3759 pEC50
Acetylcholinesterase CHEMBL3199 pIC50
Beta−lactamase CHEMBL1293244 pIC50
M17 leucyl aminopeptidase CHEMBL1293313 pIC50
Serotonin 2a (5−HT2a) receptor CHEMBL224 pKi
Cathepsin L CHEMBL3837 pKi
Cytochrome P450 3A4 CHEMBL340 pIC50
NACHT, LRR and PYD domains−containing protein 3 CHEMBL1741208 pIC50
Adenosine A1 receptor CHEMBL318 pKi
Quinone reductase 1) CHEMBL3623 pIC50
Bone morphogenetic protein 1 CHEMBL3898 pIC50
Melatonin receptor 1B CHEMBL1946 pKi
Cyclooxygenase−2 CHEMBL230 pIC50
Estrogen receptor alpha CHEMBL206 pKi
Peroxisome proliferator−activated receptor gamma CHEMBL235 pEC50
Formyl peptide receptor 1 CHEMBL3359 pIC50
Sentrin−specific protease 6 CHEMBL1741215 pIC50
Neuronal acetylcholine receptor; alpha4/beta4 CHEMBL1907595 pKi
Adenosine A1 receptor CHEMBL226 pKi
Muscarinic acetylcholine receptor M3 CHEMBL245 pKi
Aurora−A; rv–QAFFP 440
Feature importance
0.004
0.002
0.000
Calcitonin gene−related peptide type 1 receptor CHEMBL3798 pKi
Lysosomal Pro−X carboxypeptidase CHEMBL2335 pIC50
Serotonin transporter CHEMBL313 pIC50
Glucagon−like peptide 1 receptor CHEMBL1784 pEC50
Thrombin CHEMBL204 pKi
Coagulation factor X CHEMBL244 pKi
ADAM17 CHEMBL3706 pIC50
Dopamine transporter CHEMBL338 pIC50
Arachidonate 5−lipoxygenase CHEMBL215 pIC50
Acetylcholinesterase CHEMBL3199 pIC50
Peroxisome proliferator−activated receptor gamma CHEMBL235 pIC50
Human immunodeficiency virus type 1 protease CHEMBL243 pKi
Papain CHEMBL4779 pKi
Beta−2 adrenergic receptor CHEMBL210 pKi
Neurokinin 2 receptor CHEMBL2327 pKi
Muscarinic acetylcholine receptor M3 CHEMBL245 pKi
Carbonic anhydrase IX CHEMBL3594 pIC50
Neuronal acetylcholine receptor protein alpha−7 subunit CHEMBL4980 pKi
Dipeptidyl peptidase IX CHEMBL4793 pIC50
Urokinase−type plasminogen activator CHEMBL3286 pIC50
Aurora−A; b–QAFFP 440
Feature importance
0.075
0.050
0.025
0.000
Small conductance calcium−activated potassium channel protein 3 CHEMBL3780 pIC50
11−beta−hydroxysteroid dehydrogenase 1 CHEMBL3910 pIC50
Sodium/glucose cotransporter 2 CHEMBL3884 pEC50
IgG receptor FcRn large subunit p51 CHEMBL5966 pIC50
Dihydrofolate reductase CHEMBL2329 pIC50
Neuronal acetylcholine receptor protein alpha−7 subunit CHEMBL4980 pKi
Phospholipase D1 CHEMBL2536 pIC50
Cytochrome P450 19A1 CHEMBL1978 pIC50
Serine/threonine−protein kinase Chk1 CHEMBL4630 pIC50
Dihydrofolate reductase CHEMBL2425 pIC50
Dihydrofolate reductase CHEMBL202 pKi
Lysosomal Pro−X carboxypeptidase CHEMBL1255149 pIC50
Thyroid hormone receptor alpha CHEMBL1860 pIC50
Neurokinin 3 receptor CHEMBL4429 pEC50
Anandamide amidohydrolase CHEMBL3229 pIC50
Cathepsin B CHEMBL4072 pKi
Cyclin−dependent kinase 4/cyclin D1 CHEMBL1907601 pIC50
Cathepsin K CHEMBL268 pIC50
Butyrylcholinesterase CHEMBL5077 pKi
Melanocortin receptor 3 CHEMBL4644 pKi

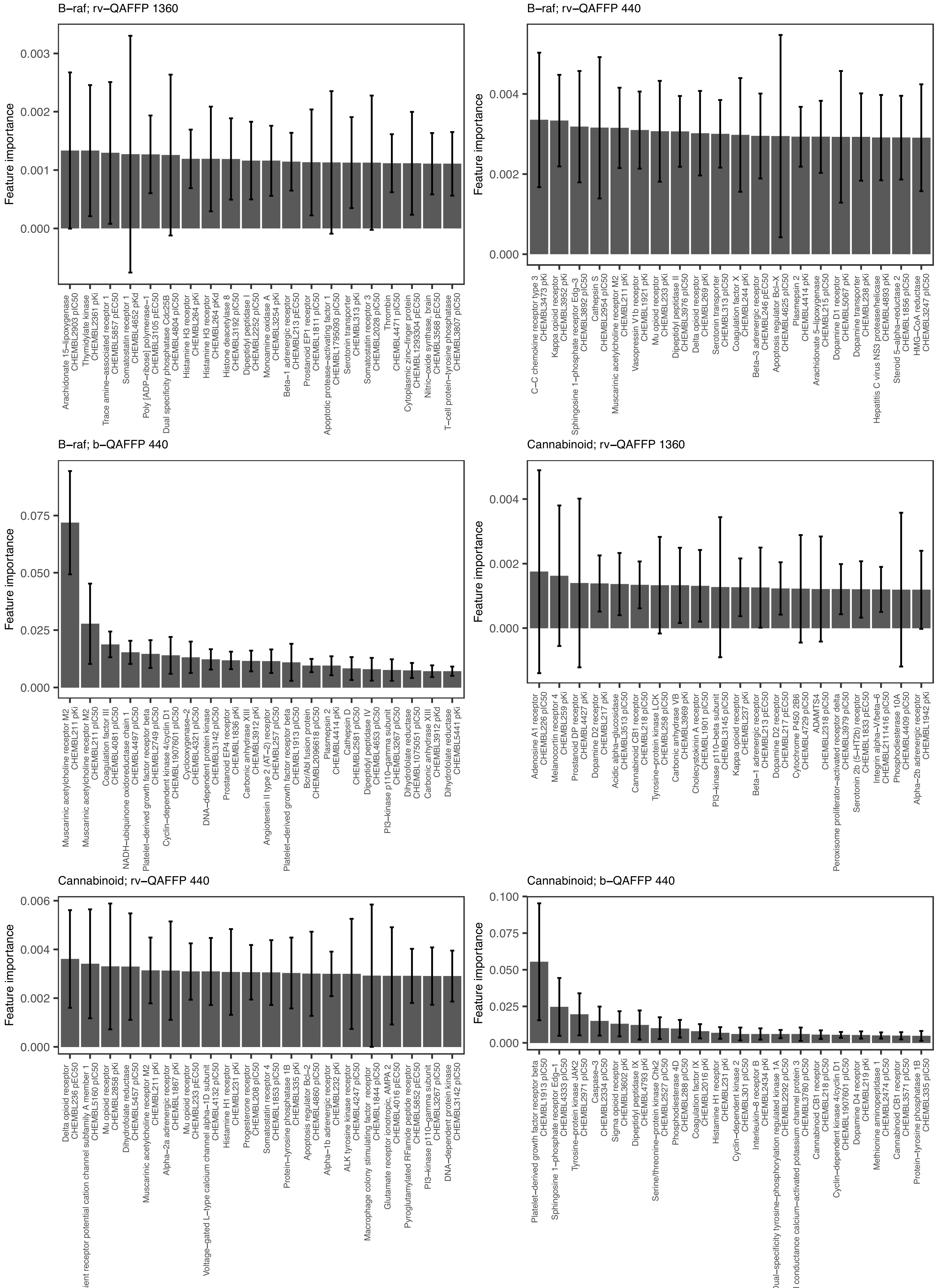
B–raf; rv–QAFFP 1360
Feature importance
0.000
0.001
0.002
0.003
Arachidonate 15–lipoxygenase CHEMBL2903 pIC50
Thymidylate kinase CHEMBL2361 pKi
Trace amine–associated receptor 1 CHEMBL5857 pEC50
Somatostatin receptor 1 CHEMBL4652 pKd
Poly [ADP–ribose] polymerase–1 CHEMBL3105 pEC50
Dual specificity phosphatase Cdc25B CHEMBL4804 pIC50
Histamine H3 receptor CHEMBL264 pKi
Histamine H3 receptor CHEMBL264 pKd
Histone deacetylase 8 CHEMBL3192 pIC50
Dipeptidyl peptidase I CHEMBL2252 pIC50
Monoamine oxidase A CHEMBL3254 pKi
Beta–1 adrenergic receptor CHEMBL213 pEC50
Prostanoid EP1 receptor CHEMBL1811 pIC50
Apoptotic protease–activating factor 1 CHEMBL1795093 pIC50
Serotonin transporter CHEMBL313 pKi
Somatostatin receptor 3 CHEMBL2028 pIC50
Thrombin CHEMBL4471 pIC50
Cytoplasmic zinc–finger protein CHEMBL1293304 pEC50
Nitric–oxide synthase, brain CHEMBL3568 pEC50
T–cell protein–tyrosine phosphatase CHEMBL3807 pIC50
B–raf; rv–QAFFP 440
Feature importance
0.000
0.002
0.004
C–C chemokine receptor type 3 CHEMBL3473 pKi
Kappa opioid receptor CHEMBL3952 pKi
Sphingosine 1–phosphate receptor Edg–3 CHEMBL3892 pIC50
Cathepsin S CHEMBL2954 pIC50
Muscarinic acetylcholine receptor M2 CHEMBL211 pKi
Vasopressin V1b receptor CHEMBL1921 pKi
Mu opioid receptor CHEMBL233 pKi
Dipeptidyl peptidase II CHEMBL3976 pIC50
Delta opioid receptor CHEMBL269 pKi
Serotonin transporter CHEMBL313 pIC50
Coagulation factor X CHEMBL244 pKi
Beta–3 adrenergic receptor CHEMBL246 pEC50
Apoptosis regulator Bcl–X CHEMBL4625 pIC50
Plasmepsin 2 CHEMBL4414 pKi
Arachidonate 5–lipoxygenase CHEMBL215 pIC50
Dopamine D1 receptor CHEMBL5067 pKi
Dopamine transporter CHEMBL238 pKi
Hepatitis C virus NS3 protease/helicase CHEMBL4893 pKi
Steroid 5–alpha–reductase 2 CHEMBL1856 pIC50
HMG–CoA reductase CHEMBL3247 pIC50
B–raf; b–QAFFP 440
Feature importance
0.000
0.025
0.050
0.075
Muscarinic acetylcholine receptor M2 CHEMBL211 pKi
Muscarinic acetylcholine receptor M2 CHEMBL211 pIC50
Coagulation factor III CHEMBL4081 pIC50
NADH–ubiquinone oxidoreductase chain 1 CHEMBL4497 pIC50
Platelet–derived growth factor receptor beta CHEMBL2749 pIC50
Cyclin–dependent kinase 4/cyclin D1 CHEMBL1907601 pIC50
Cyclooxygenase–2 CHEMBL4321 pIC50
DNA–dependent protein kinase CHEMBL3142 pIC50
Prostanoid EP4 receptor CHEMBL1836 pKi
Carbonic anhydrase XIII CHEMBL3912 pKi
Angiotensin II type 2 (AT–2) receptor CHEMBL257 pIC50
Platelet–derived growth factor receptor beta CHEMBL1913 pIC50
Bcr/Abl fusion protein CHEMBL2096618 pIC50
Plasmepsin 2 CHEMBL4414 pKi
Cathepsin D CHEMBL2581 pIC50
Dipeptidyl peptidase IV CHEMBL4653 pIC50
PI3–kinase p110–gamma subunit CHEMBL3267 pIC50
Dihydrofolate reductase CHEMBL1075051 pIC50
Carbonic anhydrase XIII CHEMBL3912 pKd
Dihydrofolate reductase CHEMBL5441 pKi
Cannabinoid; rv–QAFFP 1360
Feature importance
0.000
0.002
0.004
Adenosine A1 receptor CHEMBL226 pIC50
Melanocortin receptor 4 CHEMBL259 pKi
Prostanoid DP receptor CHEMBL4427 pKi
Dopamine D2 receptor CHEMBL217 pKi
Acidic alpha–glucosidase CHEMBL3513 pIC50
Cannabinoid CB1 receptor CHEMBL218 pIC50
Tyrosine–protein kinase LCK CHEMBL258 pKi
Carbonic anhydrase VB CHEMBL3969 pKi
Cholecystokinin A receptor CHEMBL1901 pIC50
PI3–kinase p110–beta subunit CHEMBL3145 pIC50
Kappa opioid receptor CHEMBL237 pKi
Beta–1 adrenergic receptor CHEMBL213 pEC50
Dopamine D2 receptor CHEMBL217 pEC50
Cytochrome P450 2B6 CHEMBL4729 pIC50
ADAMTS4 CHEMBL2318 pIC50
Peroxisome proliferator–activated receptor delta CHEMBL3979 pIC50
Serotonin 2b (5–HT2b) receptor CHEMBL1833 pEC50
Integrin alpha–V/beta–6 CHEMBL2111416 pIC50
Phosphodiesterase 10A CHEMBL4409 pIC50
Alpha–2b adrenergic receptor CHEMBL1942 pKi
Cannabinoid; rv–QAFFP 440
Feature importance
0.000
0.002
0.004
0.006
Delta opioid receptor CHEMBL236 pEC50
Transient receptor potential cation channel subfamily A member 1 CHEMBL5160 pIC50
Mu opioid receptor CHEMBL2858 pKi
Dihydrofolate reductase CHEMBL5457 pIC50
Muscarinic acetylcholine receptor M2 CHEMBL211 pKi
Alpha–2a adrenergic receptor CHEMBL1867 pKi
Mu opioid receptor CHEMBL233 pEC50
Voltage–gated L–type calcium channel alpha–1D subunit CHEMBL4132 pIC50
Histamine H1 receptor CHEMBL231 pKi
Progesterone receptor CHEMBL208 pIC50
Somatostatin receptor 4 CHEMBL1853 pIC50
Protein–tyrosine phosphatase 1B CHEMBL335 pKi
Apoptosis regulator Bcl–2 CHEMBL4860 pIC50
Alpha–1b adrenergic receptor CHEMBL232 pKi
ALK tyrosine kinase receptor CHEMBL4247 pIC50
Macrophage colony stimulating factor receptor CHEMBL1844 pIC50
Glutamate receptor ionotropic, AMPA 2 CHEMBL4016 pEC50
Pyroglutamylated RFamide peptide receptor CHEMBL5852 pEC50
PI3–kinase p110–gamma subunit CHEMBL3267 pIC50
DNA–dependent protein kinase CHEMBL3142 pIC50
Cannabinoid; b–QAFFP 440
Feature importance
0.000
0.025
0.050
0.075
0.100
Platelet–derived growth factor receptor beta CHEMBL1913 pIC50
Sphingosine 1–phosphate receptor Edg–1 CHEMBL4333 pIC50
Tyrosine–protein kinase JAK2 CHEMBL2971 pIC50
Caspase–3 CHEMBL2334 pIC50
Sigma opioid receptor CHEMBL3602 pKi
Dipeptidyl peptidase IX CHEMBL4793 pKi
Serine/threonine–protein kinase Chk2 CHEMBL2527 pIC50
Phosphodiesterase 4D CHEMBL288 pIC50
Coagulation factor IX CHEMBL2016 pKi
Histamine H1 receptor CHEMBL231 pIC50
Cyclin–dependent kinase 2 CHEMBL301 pIC50
Interleukin–8 receptor B CHEMBL2434 pKi
Dual–specificity tyrosine–phosphorylation regulated kinase 1A CHEMBL2292 pIC50
Small conductance calcium–activated potassium channel protein 3 CHEMBL3780 pIC50
Cannabinoid CB1 receptor CHEMBL218 pIC50
Cyclin–dependent kinase 4/cyclin D1 CHEMBL1907601 pIC50
Dopamine D4 receptor CHEMBL219 pKi
Methionine aminopeptidase 1 CHEMBL2474 pIC50
Cannabinoid CB1 receptor CHEMBL3571 pIC50
Protein–tyrosine phosphatase 1B CHEMBL335 pIC50

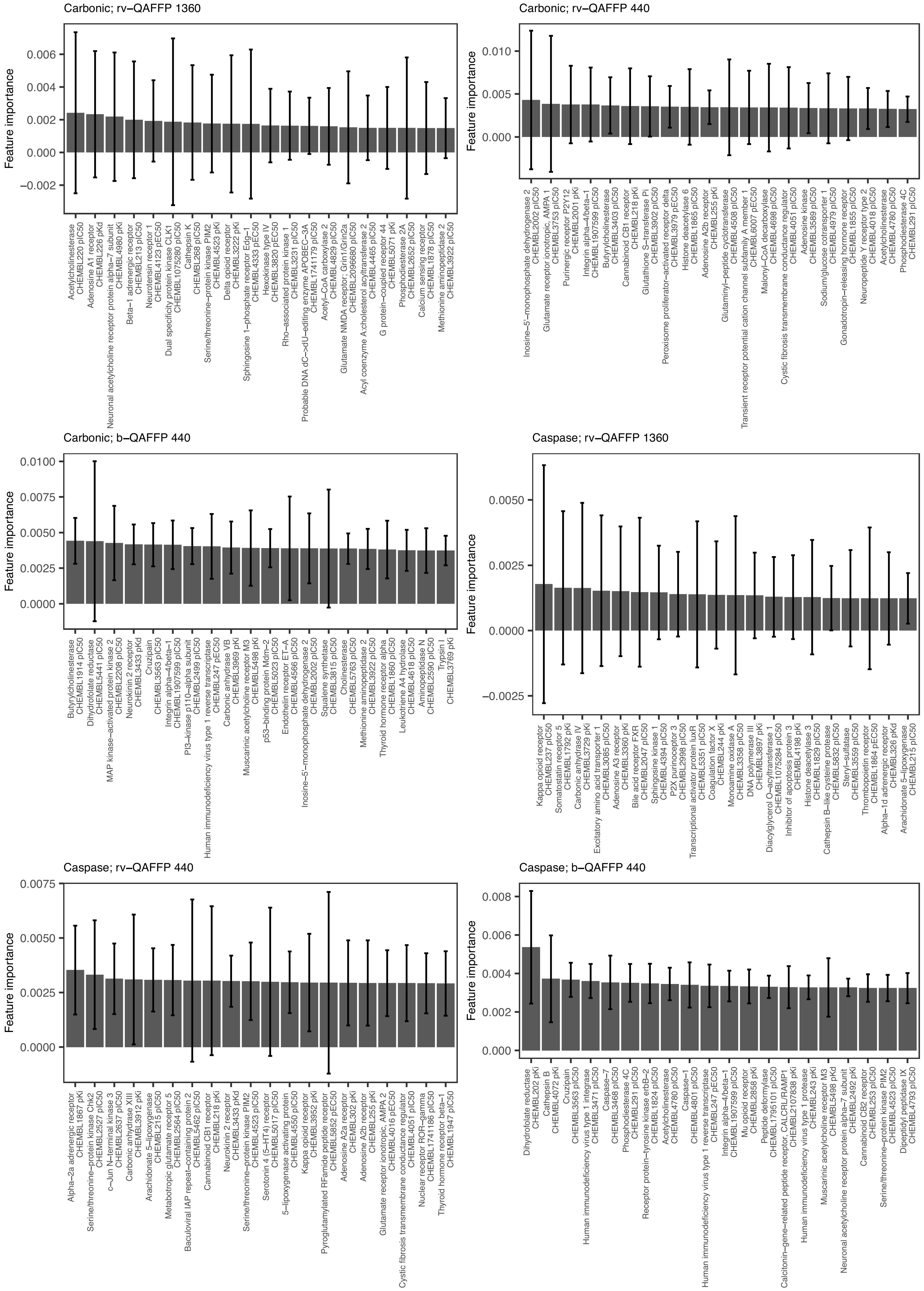

Carbonic; rv−QAFFP 1360
Feature importance
Acetylcholinesterase CHEMBL220 pIC50
Adenosine A1 receptor CHEMBL226 pKd
Neuronal acetylcholine receptor protein alpha−7 subunit CHEMBL4980 pKi
Beta−1 adrenergic receptor CHEMBL213 pIC50
Neurotensin receptor 1 CHEMBL4123 pEC50
Dual specificity protein kinase CLK1 CHEMBL1075280 pIC50
Cathepsin K CHEMBL268 pIC50
Serine/threonine−protein kinase PIM2 CHEMBL4523 pKi
Delta opioid receptor CHEMBL3222 pKi
Sphingosine 1−phosphate receptor Edg−1 CHEMBL4333 pEC50
Hexokinase type IV CHEMBL3820 pEC50
Rho−associated protein kinase 1 CHEMBL3231 pIC50
Probable DNA dC−>dU−editing enzyme APOBEC−3A CHEMBL1741179 pIC50
Acetyl−CoA carboxylase 2 CHEMBL4829 pIC50
Glutamate NMDA receptor; Grin1/Grin2a CHEMBL2096680 pIC50
Acyl coenzyme A:cholesterol acyltransferase 2 CHEMBL4465 pIC50
G protein−coupled receptor 44 CHEMBL5071 pKi
Phosphodiesterase 2A CHEMBL2652 pIC50
Calcium sensing receptor CHEMBL1878 pIC50
Methionine aminopeptidase 2 CHEMBL3922 pIC50
Carbonic; rv−QAFFP 440
Feature importance
Inosine−5'−monophosphate dehydrogenase 2 CHEMBL2002 pIC50
Glutamate receptor ionotropic, AMPA 1 CHEMBL3753 pIC50
Purinergic receptor P2Y12 CHEMBL2001 pKi
Integrin alpha−4/beta−1 CHEMBL1907599 pIC50
Butyrylcholinesterase CHEMBL3403 pIC50
Cannabinoid CB1 receptor CHEMBL218 pKi
Glutathione S−transferase Pi CHEMBL3902 pIC50
Peroxisome proliferator−activated receptor delta CHEMBL3979 pEC50
Histone deacetylase 6 CHEMBL1865 pIC50
Adenosine A2b receptor CHEMBL255 pKi
Glutaminyl−peptide cyclotransferase CHEMBL4508 pIC50
Transient receptor potential cation channel subfamily A member 1 CHEMBL6007 pEC50
Malonyl−CoA decarboxylase CHEMBL4698 pIC50
Cystic fibrosis transmembrane conductance regulator CHEMBL4051 pIC50
Adenosine kinase CHEMBL3589 pIC50
Sodium/glucose cotransporter 1 CHEMBL4979 pIC50
Gonadotropin−releasing hormone receptor CHEMBL1855 pIC50
Neuropeptide Y receptor type 2 CHEMBL4018 pIC50
Acetylcholinesterase CHEMBL4780 pIC50
Phosphodiesterase 4C CHEMBL291 pIC50
Carbonic; b−QAFFP 440
Feature importance
Butyrylcholinesterase CHEMBL1914 pIC50
Dihydrofolate reductase CHEMBL5441 pIC50
MAP kinase−activated protein kinase 2 CHEMBL2208 pIC50
Neurokinin 2 receptor CHEMBL3433 pKd
Cruzipain CHEMBL3563 pIC50
Integrin alpha−4/beta−1 CHEMBL1907599 pIC50
PI3−kinase p110−alpha subunit CHEMBL2499 pIC50
Human immunodeficiency virus type 1 reverse transcriptase CHEMBL247 pEC50
Carbonic anhydrase VB CHEMBL3969 pKi
Muscarinic acetylcholine receptor M3 CHEMBL5498 pKi
p53−binding protein Mdm−2 CHEMBL5023 pIC50
Endothelin receptor ET−A CHEMBL4566 pIC50
Inosine−5'−monophosphate dehydrogenase 2 CHEMBL2002 pIC50
Squalene synthetase CHEMBL3815 pIC50
Cholinesterase CHEMBL5763 pIC50
Methionine aminopeptidase 2 CHEMBL3922 pIC50
Thyroid hormone receptor alpha CHEMBL1860 pIC50
Leukotriene A4 hydrolase CHEMBL4618 pIC50
Aminopeptidase N CHEMBL2590 pIC50
Trypsin I CHEMBL3769 pKi
Caspase; rv−QAFFP 1360
Feature importance
Kappa opioid receptor CHEMBL237 pIC50
Somatostatin receptor 5 CHEMBL1792 pKi
Carbonic anhydrase IV CHEMBL3729 pKi
Excitatory amino acid transporter 1 CHEMBL3085 pIC50
Adenosine A3 receptor CHEMBL3360 pKi
Bile acid receptor FXR CHEMBL2047 pIC50
Sphingosine kinase 1 CHEMBL4394 pIC50
P2X purinoceptor 3 CHEMBL2998 pIC50
Transcriptional activator protein luxR CHEMBL5351 pIC50
Coagulation factor X CHEMBL244 pKi
Monoamine oxidase A CHEMBL3358 pIC50
DNA polymerase III CHEMBL3897 pKi
Diacylglycerol O−acyltransferase 1 CHEMBL1075284 pIC50
Inhibitor of apoptosis protein 3 CHEMBL4198 pKi
Histone deacetylase 3 CHEMBL1829 pIC50
Cathepsin B−like cysteine protease CHEMBL5832 pIC50
Steryl−sulfatase CHEMBL3559 pIC50
Thrombopoietin receptor CHEMBL1864 pEC50
Alpha−1d adrenergic receptor CHEMBL326 pKd
Arachidonate 5−lipoxygenase CHEMBL215 pIC50
Caspase; rv−QAFFP 440
Feature importance
Alpha−2a adrenergic receptor CHEMBL1867 pKi
Serine/threonine−protein kinase Chk2 CHEMBL2527 pIC50
c−Jun N−terminal kinase 3 CHEMBL2637 pIC50
Carbonic anhydrase XIII CHEMBL3912 pKi
Arachidonate 5−lipoxygenase CHEMBL215 pIC50
Metabotropic glutamate receptor 5 CHEMBL2564 pIC50
Baculoviral IAP repeat−containing protein 2 CHEMBL5462 pIC50
Cannabinoid CB1 receptor CHEMBL218 pKi
Neurokinin 2 receptor CHEMBL3433 pKd
Serine/threonine−protein kinase PIM2 CHEMBL4523 pIC50
Serotonin 4 (5−HT4) receptor CHEMBL5017 pIC50
5−lipoxygenase activating protein CHEMBL4550 pIC50
Kappa opioid receptor CHEMBL3952 pKi
Pyroglutamylated RFamide peptide receptor CHEMBL5852 pEC50
Adenosine A2a receptor CHEMBL302 pKi
Adenosine A2b receptor CHEMBL255 pKi
Glutamate receptor ionotropic, AMPA 2 CHEMBL4016 pEC50
Cystic fibrosis transmembrane conductance regulator CHEMBL4051 pIC50
Nuclear receptor ROR−gamma CHEMBL1741186 pIC50
Thyroid hormone receptor beta−1 CHEMBL1947 pIC50
Caspase; b−QAFFP 440
Feature importance
Dihydrofolate reductase CHEMBL202 pKi
Cathepsin B CHEMBL4072 pKi
Cruzipain CHEMBL3563 pIC50
Human immunodeficiency virus type 1 integrase CHEMBL3471 pIC50
Caspase−7 CHEMBL3468 pIC50
Phosphodiesterase 4C CHEMBL291 pIC50
Receptor protein−tyrosine kinase erbB−2 CHEMBL1824 pIC50
Acetylcholinesterase CHEMBL4780 pIC50
Caspase−1 CHEMBL4801 pIC50
Human immunodeficiency virus type 1 reverse transcriptase CHEMBL247 pEC50
Integrin alpha−4/beta−1 CHEMBL1907599 pIC50
Mu opioid receptor CHEMBL2858 pKi
Peptide deformylase CHEMBL1795101 pIC50
Calcitonin−gene−related peptide receptor; CALCRL/RAMP1 CHEMBL2107838 pIC50
Human immunodeficiency virus type 1 protease CHEMBL243 pKi
Muscarinic acetylcholine receptor M3 CHEMBL5498 pKd
Neuronal acetylcholine receptor protein alpha−7 subunit CHEMBL2492 pIC50
Cannabinoid CB2 receptor CHEMBL253 pIC50
Serine/threonine−protein kinase PIM2 CHEMBL4523 pIC50
Dipeptidyl peptidase IX CHEMBL4793 pIC50

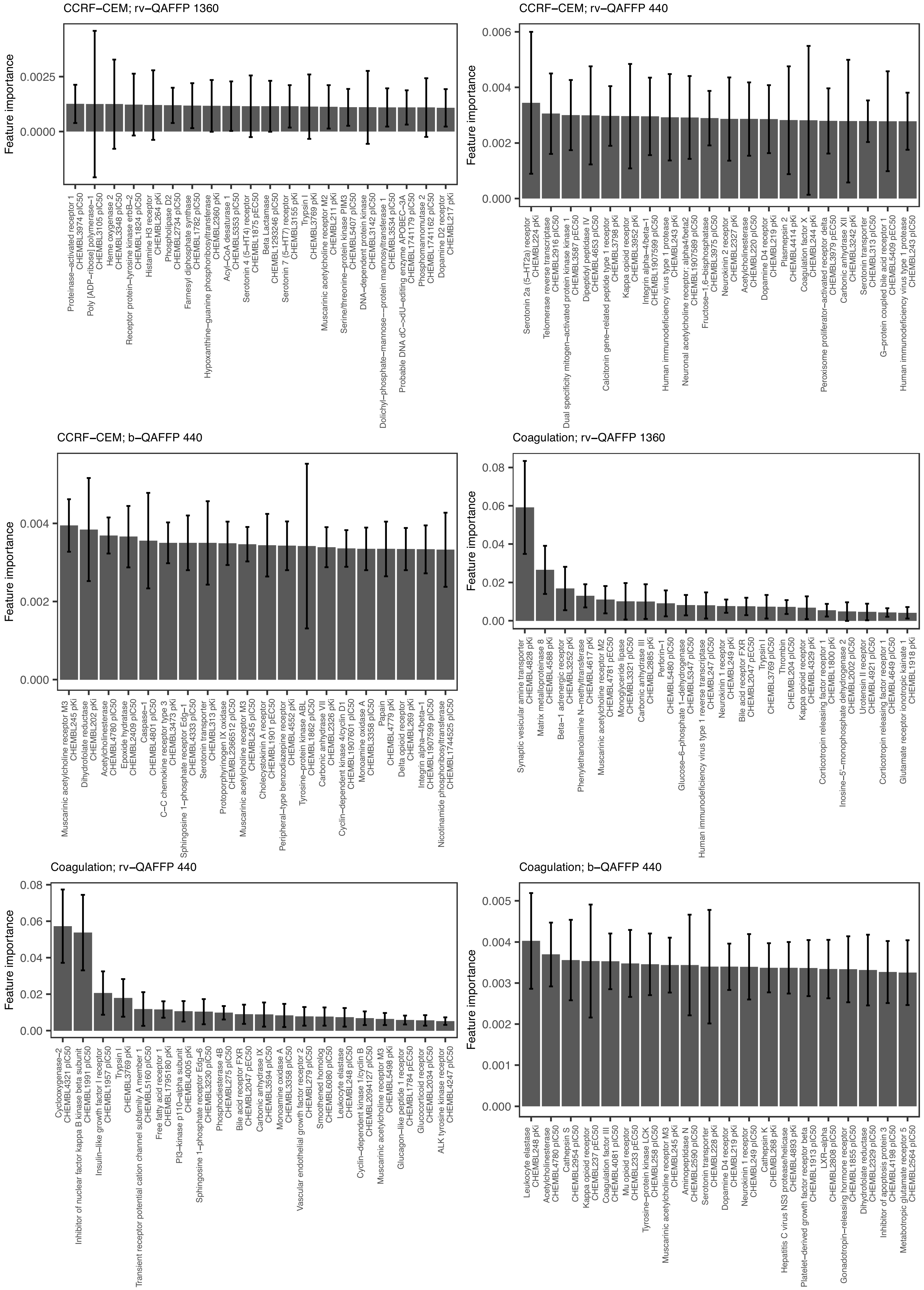
CCRF−CEM; rv−QAFFP 1360
Feature importance
0.0000
0.0025
Proteinase−activated receptor 1 CHEMBL3974 pIC50
Poly [ADP−ribose] polymerase−1 CHEMBL3105 pIC50
Heme oxygenase 2 CHEMBL3348 pIC50
Receptor protein−tyrosine kinase erbB−2 CHEMBL1824 pIC50
Histamine H3 receptor CHEMBL264 pKi
Phospholipase D2 CHEMBL2734 pIC50
Farnesyl diphosphate synthase CHEMBL1782 pIC50
Hypoxanthine−guanine phosphoribosyltransferase CHEMBL2360 pKi
Acyl−CoA desaturase 1 CHEMBL5353 pIC50
Serotonin 4 (5−HT4) receptor CHEMBL1875 pEC50
Beta Lactamase CHEMBL1293246 pIC50
Serotonin 7 (5−HT7) receptor CHEMBL3155 pKi
Trypsin I CHEMBL3769 pKi
Muscarinic acetylcholine receptor M2 CHEMBL211 pKi
Serine/threonine−protein kinase PIM3 CHEMBL5407 pIC50
DNA−dependent protein kinase CHEMBL3142 pIC50
Dolichyl−phosphate−mannose−protein mannosyltransferase 1 CHEMBL3534 pIC50
Probable DNA dC−>dU−editing enzyme APOBEC−3A CHEMBL1741179 pIC50
Phosphomannomutase 2 CHEMBL1741162 pIC50
Dopamine D2 receptor CHEMBL217 pKi
CCRF−CEM; rv−QAFFP 440
Feature importance
0.000
0.002
0.004
0.006
Serotonin 2a (5−HT2a) receptor CHEMBL224 pKi
Telomerase reverse transcriptase CHEMBL2916 pIC50
Dual specificity mitogen−activated protein kinase kinase 1 CHEMBL3587 pEC50
Dipeptidyl peptidase IV CHEMBL4653 pIC50
Calcitonin gene−related peptide type 1 receptor CHEMBL3798 pKi
Kappa opioid receptor CHEMBL3952 pKi
Integrin alpha−4/beta−1 CHEMBL1907599 pIC50
Human immunodeficiency virus type 1 protease CHEMBL243 pKi
Neuronal acetylcholine receptor; alpha4/beta2 CHEMBL1907589 pIC50
Fructose−1,6−bisphosphatase CHEMBL3975 pIC50
Neurokinin 2 receptor CHEMBL2327 pKi
Acetylcholinesterase CHEMBL220 pIC50
Dopamine D4 receptor CHEMBL219 pKi
Plasmepsin 2 CHEMBL4414 pKi
Coagulation factor X CHEMBL244 pKi
Peroxisome proliferator−activated receptor delta CHEMBL3979 pEC50
Carbonic anhydrase XII CHEMBL3242 pKi
Serotonin transporter CHEMBL313 pIC50
G−protein coupled bile acid receptor 1 CHEMBL5409 pEC50
Human immunodeficiency virus type 1 protease CHEMBL243 pIC50
CCRF−CEM; b−QAFFP 440
Feature importance
0.000
0.002
0.004
Muscarinic acetylcholine receptor M3 CHEMBL245 pKi
Dihydrofolate reductase CHEMBL202 pKi
Acetylcholinesterase CHEMBL4780 pIC50
Epoxide hydratase CHEMBL2409 pIC50
Caspase−1 CHEMBL4801 pIC50
C−C chemokine receptor type 3 CHEMBL3473 pIC50
Sphingosine 1−phosphate receptor Edg−1 CHEMBL4333 pIC50
Serotonin transporter CHEMBL313 pKi
Protoporphyrinogen IX oxidase CHEMBL2366512 pIC50
Muscarinic acetylcholine receptor M3 CHEMBL245 pIC50
Cholecystokinin A receptor CHEMBL1901 pEC50
Peripheral−type benzodiazepine receptor CHEMBL4552 pKi
Tyrosine−protein kinase ABL CHEMBL1862 pIC50
Carbonic anhydrase VII CHEMBL2326 pKi
Cyclin−dependent kinase 4/cyclin D1 CHEMBL1907601 pIC50
Monoamine oxidase A CHEMBL3358 pIC50
Papain CHEMBL4779 pKi
Delta opioid receptor CHEMBL269 pKi
Integrin alpha−4/beta−1 CHEMBL1907599 pIC50
Nicotinamide phosphoribosyltransferase CHEMBL1744525 pIC50
Coagulation; rv−QAFFP 1360
Feature importance
0.00
0.02
0.04
0.06
0.08
Synaptic vesicular amine transporter CHEMBL4828 pKi
Matrix metalloproteinase 8 CHEMBL4588 pKi
Beta−1 adrenergic receptor CHEMBL3252 pKi
Phenylethanolamine N−methyltransferase CHEMBL4617 pKi
Muscarinic acetylcholine receptor M2 CHEMBL4781 pEC50
Monoglyceride lipase CHEMBL3321 pIC50
Carbonic anhydrase III CHEMBL2885 pKi
Perforin−1 CHEMBL5480 pIC50
Glucose−6−phosphate 1−dehydrogenase CHEMBL5347 pIC50
Human immunodeficiency virus type 1 reverse transcriptase CHEMBL247 pIC50
Neurokinin 1 receptor CHEMBL249 pKi
Bile acid receptor FXR CHEMBL2047 pEC50
Trypsin I CHEMBL3769 pIC50
Thrombin CHEMBL204 pIC50
Kappa opioid receptor CHEMBL4329 pKi
Corticotropin releasing factor receptor 1 CHEMBL1800 pKi
Inosine−5'−monophosphate dehydrogenase 2 CHEMBL2002 pIC50
Urotensin II receptor CHEMBL4921 pIC50
Corticotropin releasing factor receptor 1 CHEMBL4649 pIC50
Glutamate receptor ionotropic kainate 1 CHEMBL1918 pKi
Coagulation; rv−QAFFP 440
Feature importance
0.00
0.02
0.04
0.06
0.08
Cyclooxygenase−2 CHEMBL4321 pIC50
Inhibitor of nuclear factor kappa B kinase beta subunit CHEMBL1991 pIC50
Insulin−like growth factor I receptor CHEMBL1957 pIC50
Trypsin I CHEMBL3769 pKi
Transient receptor potential cation channel subfamily A member 1 CHEMBL5160 pIC50
Free fatty acid receptor 1 CHEMBL1795180 pKi
PI3−kinase p110−alpha subunit CHEMBL4005 pKi
Sphingosine 1−phosphate receptor Edg−6 CHEMBL3230 pIC50
Phosphodiesterase 4B CHEMBL275 pIC50
Bile acid receptor FXR CHEMBL2047 pEC50
Carbonic anhydrase IX CHEMBL3594 pIC50
Monoamine oxidase A CHEMBL3358 pIC50
Vascular endothelial growth factor receptor 2 CHEMBL279 pIC50
Smoothened homolog CHEMBL6080 pIC50
Leukocyte elastase CHEMBL248 pIC50
Cyclin−dependent kinase 1/cyclin B CHEMBL2094127 pIC50
Muscarinic acetylcholine receptor M3 CHEMBL5498 pKi
Glucagon−like peptide 1 receptor CHEMBL1784 pEC50
Glucocorticoid receptor CHEMBL2034 pIC50
ALK tyrosine kinase receptor CHEMBL4247 pIC50
Coagulation; b−QAFFP 440
Feature importance
0.000
0.001
0.002
0.003
0.004
0.005
Leukocyte elastase CHEMBL248 pKi
Acetylcholinesterase CHEMBL4780 pIC50
Cathepsin S CHEMBL2954 pIC50
Kappa opioid receptor CHEMBL237 pEC50
Coagulation factor III CHEMBL4081 pIC50
Mu opioid receptor CHEMBL233 pEC50
Tyrosine−protein kinase LCK CHEMBL258 pIC50
Muscarinic acetylcholine receptor M3 CHEMBL245 pIC50
Aminopeptidase N CHEMBL2590 pIC50
Serotonin transporter CHEMBL228 pKi
Dopamine D4 receptor CHEMBL219 pKi
Neurokinin 1 receptor CHEMBL249 pIC50
Cathepsin K CHEMBL268 pKi
Hepatitis C virus NS3 protease/helicase CHEMBL4893 pKi
Platelet−derived growth factor receptor beta CHEMBL1913 pIC50
LXR−alpha CHEMBL2808 pIC50
Gonadotropin−releasing hormone receptor CHEMBL1855 pIC50
Dihydrofolate reductase CHEMBL2329 pIC50
Inhibitor of apoptosis protein 3 CHEMBL4198 pIC50
Metabotropic glutamate receptor 5 CHEMBL2564 pIC50

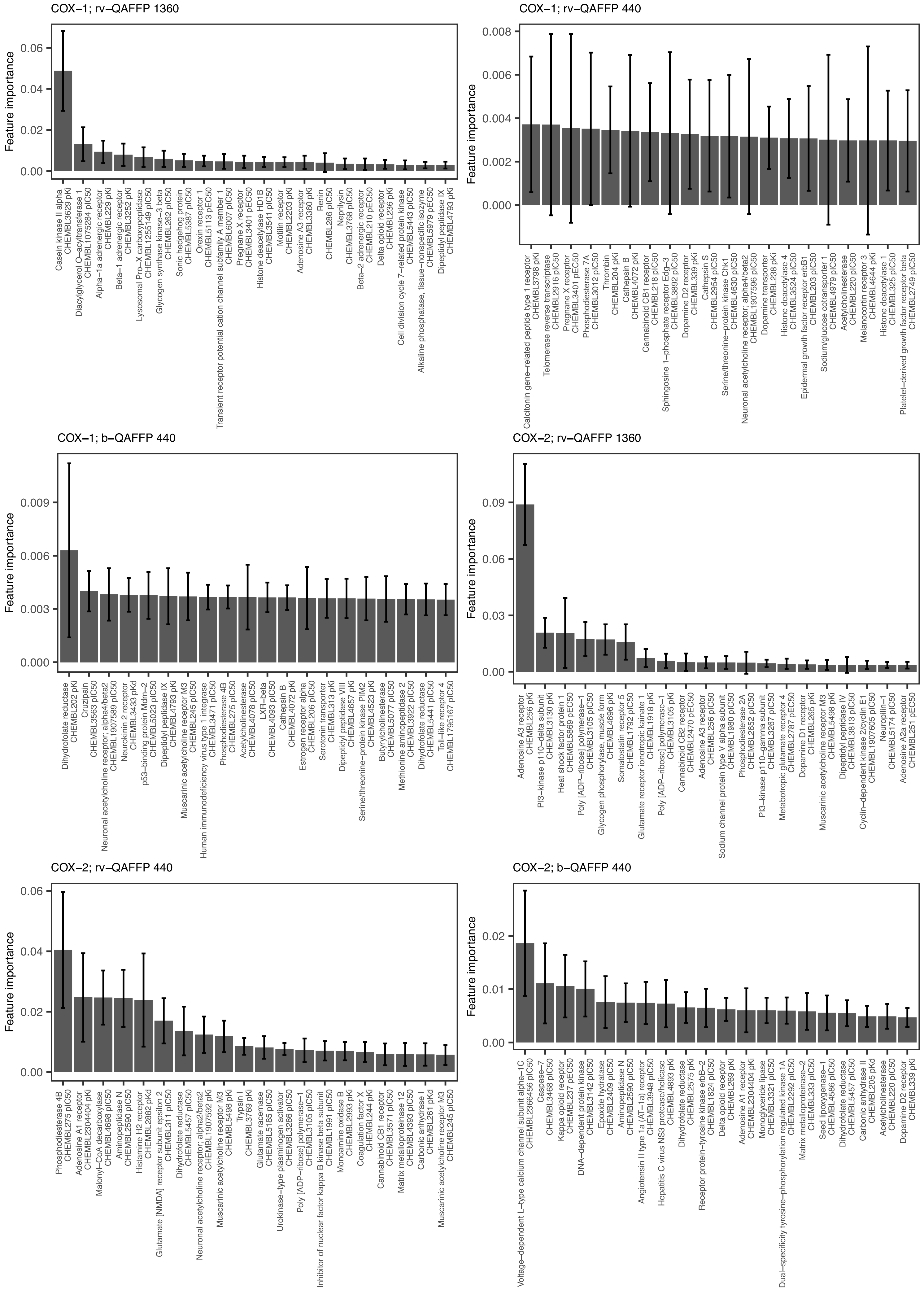
COX−1; rv−QAFFP 1360
Feature importance
Casein kinase II alpha CHEMBL3629 pKi
Diacylglycerol O−acyltransferase 1 CHEMBL1075284 pIC50
Alpha−1a adrenergic receptor CHEMBL229 pKi
Beta−1 adrenergic receptor CHEMBL3252 pKi
Lysosomal Pro−X carboxypeptidase CHEMBL1255149 pIC50
Glycogen synthase kinase−3 beta CHEMBL262 pIC50
Sonic hedgehog protein CHEMBL5387 pIC50
Orexin receptor 1 CHEMBL5113 pEC50
Transient receptor potential cation channel subfamily A member 1 CHEMBL6007 pIC50
Pregnane X receptor CHEMBL3401 pEC50
Histone deacetylase HD1B CHEMBL3541 pIC50
Motilin receptor CHEMBL2203 pKi
Adenosine A3 receptor CHEMBL3360 pKi
Renin CHEMBL286 pIC50
Neprilysin CHEMBL3768 pIC50
Beta−2 adrenergic receptor CHEMBL210 pEC50
Delta opioid receptor CHEMBL236 pKi
Cell division cycle 7−related protein kinase CHEMBL5443 pIC50
Alkaline phosphatase, tissue−nonspecific isozyme CHEMBL5979 pEC50
Dipeptidyl peptidase IX CHEMBL4793 pKi
COX−1; rv−QAFFP 440
Feature importance
Calcitonin gene−related peptide type 1 receptor CHEMBL3798 pKi
Telomerase reverse transcriptase CHEMBL2916 pIC50
Pregnane X receptor CHEMBL3401 pEC50
Phosphodiesterase 7A CHEMBL3012 pIC50
Thrombin CHEMBL204 pKi
Cathepsin B CHEMBL4072 pKi
Cannabinoid CB1 receptor CHEMBL218 pIC50
Sphingosine 1−phosphate receptor Edg−3 CHEMBL3892 pIC50
Dopamine D2 receptor CHEMBL339 pKi
Cathepsin S CHEMBL2954 pIC50
Serine/threonine−protein kinase Chk1 CHEMBL4630 pIC50
Neuronal acetylcholine receptor; alpha4/beta2 CHEMBL1907596 pIC50
Dopamine transporter CHEMBL238 pKi
Histone deacetylase 4 CHEMBL3524 pIC50
Epidermal growth factor receptor erbB1 CHEMBL203 pIC50
Sodium/glucose cotransporter 1 CHEMBL4979 pIC50
Acetylcholinesterase CHEMBL220 pIC50
Melanocortin receptor 3 CHEMBL4644 pKi
Histone deacetylase 1 CHEMBL325 pIC50
Platelet−derived growth factor receptor beta CHEMBL2749 pIC50
COX−1; b−QAFFP 440
Feature importance
Dihydrofolate reductase CHEMBL202 pKi
Cruzipain CHEMBL3563 pIC50
Neuronal acetylcholine receptor; alpha4/beta2 CHEMBL1907589 pIC50
Neurokinin 2 receptor CHEMBL3433 pKd
p53−binding protein Mdm−2 CHEMBL5023 pIC50
Dipeptidyl peptidase IX CHEMBL4793 pIC50
Muscarinic acetylcholine receptor M3 CHEMBL245 pIC50
Human immunodeficiency virus type 1 integrase CHEMBL3471 pIC50
Phosphodiesterase 4B CHEMBL275 pIC50
Acetylcholinesterase CHEMBL4078 pIC50
LXR−beta CHEMBL4093 pIC50
Cathepsin B CHEMBL4072 pIC50
Estrogen receptor alpha CHEMBL206 pIC50
Serotonin transporter CHEMBL313 pKi
Dipeptidyl peptidase VIII CHEMBL4657 pKi
Serine/threonine−protein kinase PIM2 CHEMBL4523 pKi
Butyrylcholinesterase CHEMBL5077 pIC50
Methionine aminopeptidase 2 CHEMBL3922 pIC50
Dihydrofolate reductase CHEMBL5441 pIC50
Toll−like receptor 4 CHEMBL1795167 pIC50
COX−2; rv−QAFFP 1360
Feature importance
Adenosine A3 receptor CHEMBL256 pKi
PI3−kinase p110−delta subunit CHEMBL3130 pKi
Heat shock factor protein 1 CHEMBL5869 pEC50
Poly [ADP−ribose] polymerase−1 CHEMBL3105 pIC50
Glycogen phosphorylase, muscle form CHEMBL4696 pKi
Somatostatin receptor 5 CHEMBL1792 pIC50
Glutamate receptor ionotropic kainate 1 CHEMBL1918 pKi
Poly [ADP−ribose] polymerase−1 CHEMBL3105 pKi
Cannabinoid CB2 receptor CHEMBL2470 pEC50
Adenosine A3 receptor CHEMBL256 pIC50
Sodium channel protein type V alpha subunit CHEMBL1980 pIC50
Phosphodiesterase 2A CHEMBL2652 pIC50
PI3−kinase p110−gamma subunit CHEMBL3267 pIC50
Metabotropic glutamate receptor 4 CHEMBL2787 pEC50
Dopamine D1 receptor CHEMBL265 pKi
Muscarinic acetylcholine receptor M3 CHEMBL5498 pKi
Dipeptidyl peptidase IV CHEMBL3813 pIC50
Cyclin−dependent kinase 2/cyclin E1 CHEMBL1907605 pIC50
Neuropilin−1 CHEMBL5174 pIC50
Adenosine A2a receptor CHEMBL251 pEC50
COX−2; rv−QAFFP 440
Feature importance
Phosphodiesterase 4B CHEMBL275 pIC50
Adenosine A1 receptor CHEMBL2304404 pKi
Malonyl−CoA decarboxylase CHEMBL4698 pIC50
Aminopeptidase N CHEMBL2590 pIC50
Histamine H2 receptor CHEMBL2882 pKd
Glutamate [NMDA] receptor subunit epsilon 2 CHEMBL311 pIC50
Dihydrofolate reductase CHEMBL5457 pIC50
Neuronal acetylcholine receptor; alpha2/beta2 CHEMBL1907592 pKi
Muscarinic acetylcholine receptor M3 CHEMBL5498 pKi
Trypsin I CHEMBL3769 pKi
Glutamate racemase CHEMBL5185 pIC50
Urokinase−type plasminogen activator CHEMBL3286 pIC50
Poly [ADP−ribose] polymerase−1 CHEMBL3105 pIC50
Inhibitor of nuclear factor kappa B kinase beta subunit CHEMBL1991 pIC50
Monoamine oxidase B CHEMBL2993 pKi
Coagulation factor X CHEMBL244 pKi
Cannabinoid CB1 receptor CHEMBL3571 pIC50
Matrix metalloproteinase 12 CHEMBL4393 pIC50
Carbonic anhydrase I CHEMBL261 pKd
Muscarinic acetylcholine receptor M3 CHEMBL245 pIC50
COX−2; b−QAFFP 440
Feature importance
Voltage−dependent L−type calcium channel subunit alpha−1C CHEMBL2366456 pIC50
Caspase−7 CHEMBL3468 pIC50
Kappa opioid receptor CHEMBL237 pEC50
DNA−dependent protein kinase CHEMBL3142 pIC50
Epoxide hydratase CHEMBL2409 pIC50
Aminopeptidase N CHEMBL2590 pIC50
Angiotensin II type 1a (AT−1a) receptor CHEMBL3948 pIC50
Hepatitis C virus NS3 protease/helicase CHEMBL4893 pKi
Dihydrofolate reductase CHEMBL2575 pKi
Receptor protein−tyrosine kinase erbB−2 CHEMBL1824 pIC50
Delta opioid receptor CHEMBL269 pKi
Adenosine A1 receptor CHEMBL2304404 pKi
Monoglyceride lipase CHEMBL3321 pIC50
Dual−specificity tyrosine−phosphorylation regulated kinase 1A CHEMBL2292 pIC50
Matrix metalloproteinase−2 CHEMBL333 pIC50
Seed lipoxygenase−1 CHEMBL4586 pIC50
Dihydrofolate reductase CHEMBL5457 pIC50
Carbonic anhydrase II CHEMBL205 pKd
Acetylcholinesterase CHEMBL220 pIC50
Dopamine D2 receptor CHEMBL339 pKi

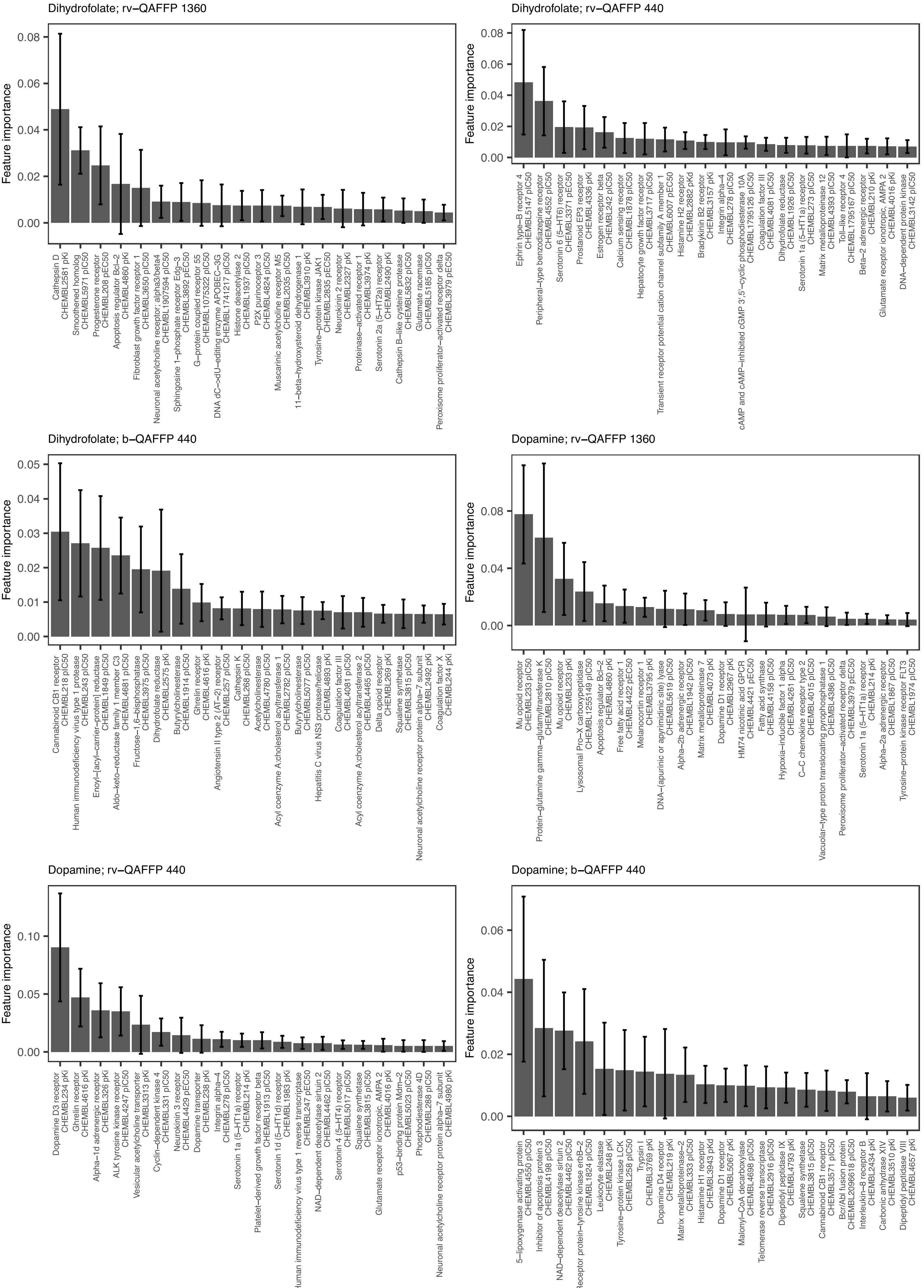

Dihydrofolate; rv−QAFFP 1360
Feature importance
0.00
0.02
0.04
0.06
0.08
Cathepsin D CHEMBL2581 pIC50
Smoothened homolog CHEMBL5971 pIC50
Progesterone receptor CHEMBL208 pEC50
Apoptosis regulator Bcl−2 CHEMBL4860 pKi
Fibroblast growth factor receptor 1 CHEMBL3650 pIC50
Neuronal acetylcholine receptor; alpha3/beta4 CHEMBL1907594 pIC50
Sphingosine 1−phosphate receptor Edg−3 CHEMBL3892 pEC50
G−protein coupled receptor 55 CHEMBL1075322 pIC50
DNA dC−>dU−editing enzyme APOBEC−3G CHEMBL1741217 pIC50
Histone deacetylase 2 CHEMBL1937 pIC50
P2X purinoceptor 3 CHEMBL4824 pIC50
Muscarinic acetylcholine receptor M5 CHEMBL2035 pIC50
11−beta−hydroxysteroid dehydrogenase 1 CHEMBL3910 pKi
Tyrosine−protein kinase JAK1 CHEMBL2835 pEC50
Neurokinin 2 receptor CHEMBL2327 pKi
Proteinase−activated receptor 1 CHEMBL3974 pIC50
Serotonin 2a (5−HT2a) receptor CHEMBL2490 pKi
Cathepsin B−like cysteine protease CHEMBL5832 pIC50
Glutamate racemase CHEMBL5185 pIC50
Peroxisome proliferator−activated receptor delta CHEMBL3979 pEC50
Dihydrofolate; rv−QAFFP 440
Feature importance
0.00
0.02
0.04
0.06
0.08
Ephrin type−B receptor 4 CHEMBL5147 pIC50
Peripheral−type benzodiazepine receptor CHEMBL4552 pIC50
Serotonin 6 (5−HT6) receptor CHEMBL3371 pEC50
Prostanoid EP3 receptor CHEMBL4336 pKi
Estrogen receptor beta CHEMBL242 pIC50
Calcium sensing receptor CHEMBL1878 pIC50
Hepatocyte growth factor receptor CHEMBL3717 pIC50
Transient receptor potential cation channel subfamily A member 1 CHEMBL6007 pEC50
Histamine H2 receptor CHEMBL2882 pKd
Bradykinin B2 receptor CHEMBL3157 pKi
Integrin alpha−4 CHEMBL278 pIC50
cAMP and cAMP−inhibited cGMP 3',5'−cyclic phosphodiesterase 10A CHEMBL1795126 pIC50
Coagulation factor III CHEMBL4081 pIC50
Dihydrofolate reductase CHEMBL1926 pIC50
Serotonin 1a (5−HT1a) receptor CHEMBL273 pIC50
Matrix metalloproteinase 12 CHEMBL4393 pIC50
Toll−like receptor 4 CHEMBL1795167 pIC50
Beta−2 adrenergic receptor CHEMBL210 pKi
Glutamate receptor ionotropic, AMPA 2 CHEMBL4016 pKi
DNA−dependent protein kinase CHEMBL3142 pIC50
Dihydrofolate; b−QAFFP 440
Feature importance
0.00
0.01
0.02
0.03
0.04
0.05
Cannabinoid CB1 receptor CHEMBL218 pIC50
Human immunodeficiency virus type 1 protease CHEMBL243 pIC50
Enoyl−[acyl−carrier−protein] reductase CHEMBL1849 pIC50
Aldo−keto−reductase family 1 member C3 CHEMBL4681 pIC50
Fructose−1,6−bisphosphatase CHEMBL3975 pIC50
Dihydrofolate reductase CHEMBL2575 pKi
Butyrylcholinesterase CHEMBL1914 pIC50
Ghrelin receptor CHEMBL4616 pKi
Angiotensin II type 2 (AT−2) receptor CHEMBL257 pIC50
Cathepsin K CHEMBL268 pIC50
Acetylcholinesterase CHEMBL4780 pIC50
Acyl coenzyme A:cholesterol acyltransferase 1 CHEMBL2782 pIC50
Butyrylcholinesterase CHEMBL5077 pIC50
Hepatitis C virus NS3 protease/helicase CHEMBL4893 pKi
Coagulation factor III CHEMBL4081 pIC50
Acyl coenzyme A:cholesterol acyltransferase 2 CHEMBL4465 pIC50
Delta opioid receptor CHEMBL269 pKi
Squalene synthetase CHEMBL3815 pIC50
Neuronal acetylcholine receptor protein alpha−7 subunit CHEMBL2492 pKi
Coagulation factor X CHEMBL244 pKi
Dopamine; rv−QAFFP 1360
Feature importance
0.00
0.04
0.08
Mu opioid receptor CHEMBL233 pIC50
Protein−glutamine gamma−glutamyltransferase K CHEMBL2810 pIC50
Mu opioid receptor CHEMBL233 pKi
Lysosomal Pro−X carboxypeptidase CHEMBL1255149 pIC50
Apoptosis regulator Bcl−2 CHEMBL4860 pKi
Free fatty acid receptor 1 CHEMBL4422 pEC50
Melanocortin receptor 1 CHEMBL3795 pKi
DNA−(apurinic or apyrimidinic site) lyase CHEMBL5619 pIC50
Alpha−2b adrenergic receptor CHEMBL1942 pIC50
Matrix metalloproteinase 7 CHEMBL4073 pKi
Dopamine D1 receptor CHEMBL2967 pKi
HM74 nicotinic acid GPCR CHEMBL4421 pEC50
Fatty acid synthase CHEMBL4158 pIC50
Hypoxia−inducible factor 1 alpha CHEMBL4261 pIC50
C−C chemokine receptor type 2 CHEMBL4015 pIC50
Vacuolar−type proton translocating pyrophosphatase 1 CHEMBL4386 pIC50
Peroxisome proliferator−activated receptor delta CHEMBL3979 pEC50
Serotonin 1a (5−HT1a) receptor CHEMBL214 pKi
Alpha−2a adrenergic receptor CHEMBL1867 pIC50
Tyrosine−protein kinase receptor FLT3 CHEMBL1974 pIC50
Dopamine; rv−QAFFP 440
Feature importance
0.00
0.05
0.10
Dopamine D3 receptor CHEMBL234 pKi
Ghrelin receptor CHEMBL4616 pKi
Alpha−1d adrenergic receptor CHEMBL326 pKi
ALK tyrosine kinase receptor CHEMBL4247 pIC50
Vesicular acetylcholine transporter CHEMBL3313 pKi
Cyclin−dependent kinase 4 CHEMBL331 pIC50
Neurokinin 3 receptor CHEMBL4429 pEC50
Dopamine transporter CHEMBL238 pKi
Integrin alpha−4 CHEMBL278 pIC50
Serotonin 1a (5−HT1a) receptor CHEMBL214 pKi
Platelet−derived growth factor receptor beta CHEMBL1913 pIC50
Serotonin 1d (5−HT1d) receptor CHEMBL1983 pKi
Human immunodeficiency virus type 1 reverse transcriptase CHEMBL247 pEC50
NAD−dependent deacetylase sirtuin 2 CHEMBL4462 pIC50
Serotonin 4 (5−HT4) receptor CHEMBL5017 pIC50
Squalene synthetase CHEMBL3815 pIC50
Glutamate receptor ionotropic, AMPA 2 CHEMBL4016 pKi
p53−binding protein Mdm−2 CHEMBL5023 pIC50
Phosphodiesterase 4D CHEMBL288 pIC50
Neuronal acetylcholine receptor protein alpha−7 subunit CHEMBL4980 pKi
Dopamine; b−QAFFP 440
Feature importance
0.00
0.02
0.04
0.06
5−lipoxygenase activating protein CHEMBL4550 pIC50
Inhibitor of apoptosis protein 3 CHEMBL4198 pIC50
NAD−dependent deacetylase sirtuin 2 CHEMBL4462 pIC50
Receptor protein−tyrosine kinase erbB−2 CHEMBL1824 pIC50
Leukocyte elastase CHEMBL248 pIC50
Tyrosine−protein kinase LCK CHEMBL258 pIC50
Trypsin I CHEMBL3769 pKi
Dopamine D4 receptor CHEMBL219 pKi
Matrix metalloproteinase−2 CHEMBL333 pIC50
Histamine H1 receptor CHEMBL3943 pKd
Dopamine D1 receptor CHEMBL5067 pIC50
Malonyl−CoA decarboxylase CHEMBL4698 pIC50
Telomerase reverse transcriptase CHEMBL2916 pIC50
Dipeptidyl peptidase IX CHEMBL4793 pKi
Squalene synthetase CHEMBL3815 pIC50
Cannabinoid CB1 receptor CHEMBL3571 pIC50
Bcr/Abl fusion protein CHEMBL2096618 pIC50
Interleukin−8 receptor B CHEMBL2434 pKi
Carbonic anhydrase XIV CHEMBL3510 pKi
Dipeptidyl peptidase VIII CHEMBL4657 pKi

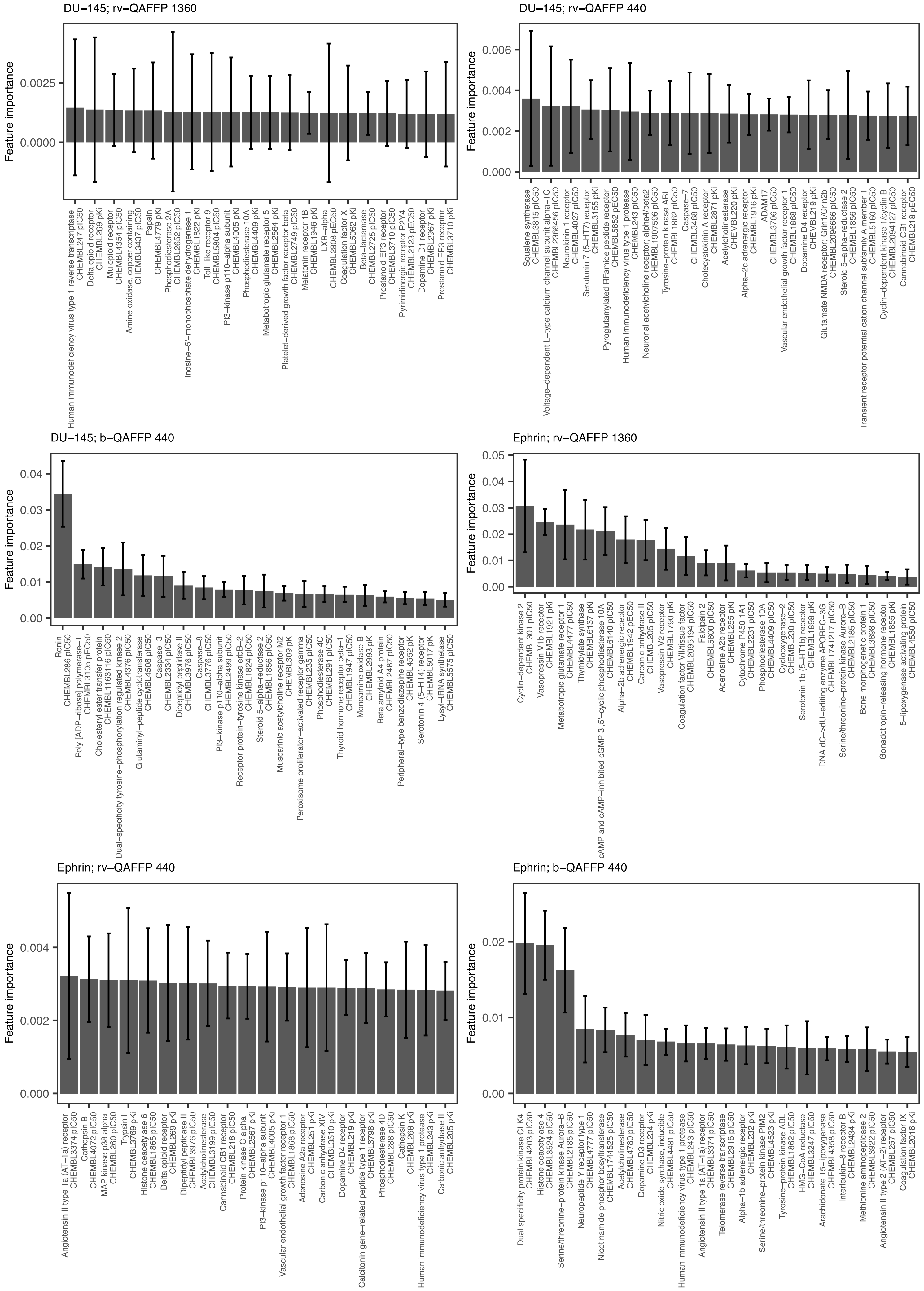
DU–145; rv–QAFFP 1360
Feature importance
0.0000
0.0025
Human immunodeficiency virus type 1 reverse transcriptase CHEMBL247 pIC50
Delta opioid receptor CHEMBL269 pKi
Mu opioid receptor CHEMBL4354 pIC50
Amine oxidase, copper containing CHEMBL3437 pIC50
Papain CHEMBL4779 pKi
Phosphodiesterase 2A CHEMBL2652 pIC50
Inosine−5'−monophosphate dehydrogenase 1 CHEMBL1822 pKi
Toll−like receptor 9 CHEMBL5804 pIC50
PI3−kinase p110−alpha subunit CHEMBL4005 pKi
Phosphodiesterase 10A CHEMBL4409 pKi
Metabotropic glutamate receptor 5 CHEMBL2564 pKi
Platelet−derived growth factor receptor beta CHEMBL2749 pIC50
Melatonin receptor 1B CHEMBL1946 pKi
LXR−alpha CHEMBL2808 pEC50
Coagulation factor X CHEMBL5062 pKi
Beta−lactamase CHEMBL2725 pIC50
Prostanoid EP3 receptor CHEMBL3710 pIC50
Pyrimidinergic receptor P2Y4 CHEMBL2123 pEC50
Dopamine D1 receptor CHEMBL2967 pKi
Prostanoid EP3 receptor CHEMBL3710 pKi
DU–145; rv–QAFFP 440
Feature importance
0.000
0.002
0.004
0.006
Squalene synthetase CHEMBL3815 pIC50
Voltage−dependent L−type calcium channel subunit alpha−1C CHEMBL2366456 pIC50
Neurokinin 1 receptor CHEMBL4027 pIC50
Serotonin 7 (5−HT7) receptor CHEMBL3155 pKi
Pyroglutamylated RFamide peptide receptor CHEMBL5852 pEC50
Human immunodeficiency virus type 1 protease CHEMBL243 pIC50
Neuronal acetylcholine receptor; alpha4/beta2 CHEMBL1907596 pIC50
Tyrosine−protein kinase ABL CHEMBL1862 pIC50
Caspase−7 CHEMBL3468 pIC50
Cholecystokinin A receptor CHEMBL2871 pKi
Acetylcholinesterase CHEMBL220 pKi
Alpha−2c adrenergic receptor CHEMBL1916 pKi
ADAM17 CHEMBL3706 pIC50
Vascular endothelial growth factor receptor 1 CHEMBL1868 pIC50
Dopamine D4 receptor CHEMBL219 pKi
Glutamate NMDA receptor; Grin1/Grin2b CHEMBL2096666 pIC50
Steroid 5−alpha−reductase 2 CHEMBL1856 pIC50
Transient receptor potential cation channel subfamily A member 1 CHEMBL5160 pIC50
Cyclin−dependent kinase 1/cyclin B CHEMBL2094127 pIC50
Cannabinoid CB1 receptor CHEMBL218 pEC50
DU–145; b–QAFFP 440
Feature importance
0.00
0.01
0.02
0.03
0.04
Renin CHEMBL286 pIC50
Poly [ADP−ribose] polymerase−1 CHEMBL3105 pEC50
Cholesteryl ester transfer protein CHEMBL3116 pIC50
Dual−specificity tyrosine−phosphorylation regulated kinase 2 CHEMBL4376 pIC50
Glutaminyl−peptide cyclotransferase CHEMBL4508 pIC50
Caspase−3 CHEMBL2334 pIC50
Dipeptidyl peptidase II CHEMBL3976 pIC50
Caspase−8 CHEMBL3776 pIC50
PI3−kinase p110−alpha subunit CHEMBL2499 pIC50
Receptor protein−tyrosine kinase erbB−2 CHEMBL1824 pIC50
Steroid 5−alpha−reductase 2 CHEMBL1856 pIC50
Muscarinic acetylcholine receptor M2 CHEMBL309 pKi
Peroxisome proliferator−activated receptor gamma CHEMBL235 pIC50
Phosphodiesterase 4C CHEMBL291 pIC50
Thyroid hormone receptor beta−1 CHEMBL1947 pIC50
Monoamine oxidase B CHEMBL2993 pKi
Beta amyloid A4 protein CHEMBL2487 pIC50
Peripheral−type benzodiazepine receptor CHEMBL4552 pKi
Serotonin 4 (5−HT4) receptor CHEMBL5017 pKi
Lysyl−tRNA synthetase CHEMBL5575 pIC50
Ephrin; rv–QAFFP 1360
Feature importance
0.00
0.01
0.02
0.03
0.04
0.05
Cyclin−dependent kinase 2 CHEMBL301 pIC50
Vasopressin V1b receptor CHEMBL1921 pKi
Metabotropic glutamate receptor 1 CHEMBL4477 pIC50
Thymidylate synthase CHEMBL6137 pKi
cAMP and cAMP−inhibited cGMP 3',5'−cyclic phosphodiesterase 10A CHEMBL6140 pIC50
Alpha−2b adrenergic receptor CHEMBL1942 pEC50
Carbonic anhydrase II CHEMBL205 pIC50
Vasopressin V2 receptor CHEMBL1790 pKi
Coagulation factor VII/tissue factor CHEMBL2095194 pIC50
Falcipain 2 CHEMBL5800 pIC50
Adenosine A2b receptor CHEMBL255 pKi
Cytochrome P450 1A1 CHEMBL2231 pIC50
Phosphodiesterase 10A CHEMBL4409 pIC50
Cyclooxygenase−2 CHEMBL230 pIC50
Serotonin 1b (5−HT1b) receptor CHEMBL1898 pKi
DNA dC−>dU−editing enzyme APOBEC−3G CHEMBL1741217 pIC50
Serine/threonine−protein kinase Aurora−B CHEMBL2185 pIC50
Bone morphogenetic protein 1 CHEMBL3898 pIC50
Gonadotropin−releasing hormone receptor CHEMBL1855 pKi
5−lipoxygenase activating protein CHEMBL4550 pIC50
Ephrin; rv–QAFFP 440
Feature importance
0.000
0.002
0.004
Angiotensin II type 1a (AT−1a) receptor CHEMBL3374 pIC50
Cathepsin B CHEMBL4072 pIC50
MAP kinase p38 alpha CHEMBL260 pIC50
Trypsin I CHEMBL3769 pKi
Histone deacetylase 6 CHEMBL1865 pIC50
Delta opioid receptor CHEMBL269 pKi
Dipeptidyl peptidase II CHEMBL3976 pIC50
Acetylcholinesterase CHEMBL3199 pIC50
Cannabinoid CB1 receptor CHEMBL218 pIC50
Protein kinase C alpha CHEMBL2567 pKi
PI3−kinase p110−alpha subunit CHEMBL4005 pIC50
Vascular endothelial growth factor receptor 1 CHEMBL1868 pIC50
Adenosine A2a receptor CHEMBL251 pKi
Carbonic anhydrase XIV CHEMBL3510 pKi
Dopamine D4 receptor CHEMBL219 pIC50
Calcitonin gene−related peptide type 1 receptor CHEMBL3798 pIC50
Phosphodiesterase 4D CHEMBL288 pIC50
Cathepsin K CHEMBL268 pKi
Human immunodeficiency virus type 1 protease CHEMBL243 pKi
Carbonic anhydrase II CHEMBL205 pKi
Ephrin; b–QAFFP 440
Feature importance
0.00
0.01
0.02
Dual specificity protein kinase CLK4 CHEMBL4203 pIC50
Histone deacetylase 4 CHEMBL3524 pIC50
Serine/threonine−protein kinase Aurora−B CHEMBL2185 pIC50
Neuropeptide Y receptor type 1 CHEMBL4777 pIC50
Nicotinamide phosphoribosyltransferase CHEMBL1744525 pIC50
Acetylcholinesterase CHEMBL4780 pIC50
Dopamine D3 receptor CHEMBL234 pKi
Nitric oxide synthase, inducible CHEMBL4481 pIC50
Human immunodeficiency virus type 1 protease CHEMBL243 pIC50
Angiotensin II type 1a (AT−1a) receptor CHEMBL3374 pIC50
Telomerase reverse transcriptase CHEMBL2916 pIC50
Alpha−1b adrenergic receptor CHEMBL232 pKi
Serine/threonine−protein kinase PIM2 CHEMBL4523 pKi
Tyrosine−protein kinase ABL CHEMBL1862 pIC50
HMG−CoA reductase CHEMBL3247 pIC50
Arachidonate 15−lipoxygenase CHEMBL4358 pIC50
Interleukin−8 receptor B CHEMBL2434 pKi
Methionine aminopeptidase 2 CHEMBL3922 pIC50
Angiotensin II type 2 (AT−2) receptor CHEMBL257 pIC50
Coagulation factor IX CHEMBL2016 pKi

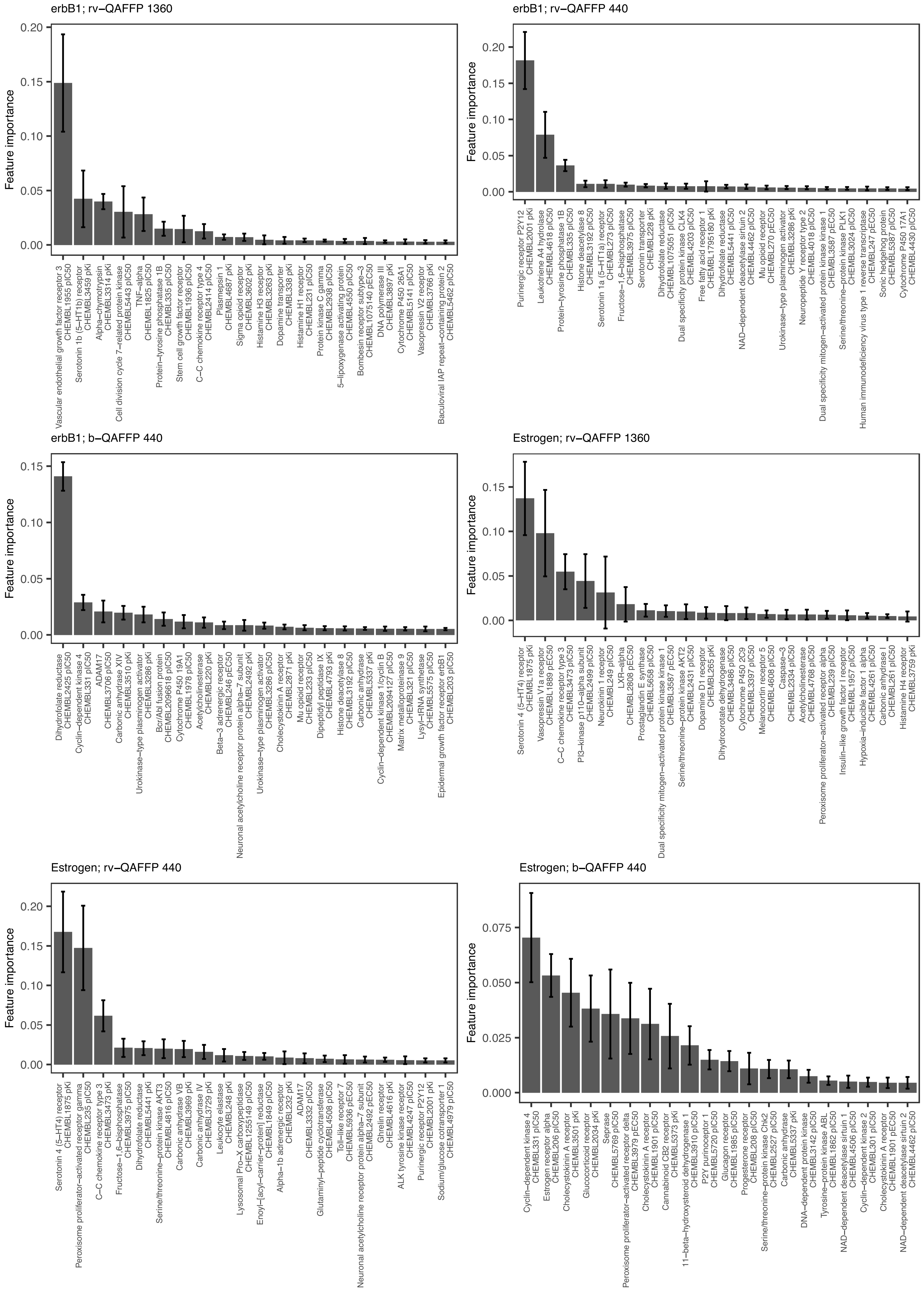
erbB1; rv−QAFFP 1360
Feature importance
0.00
0.05
0.10
0.15
0.20
Vascular endothelial growth factor receptor 3 CHEMBL1955 pIC50
Serotonin 1b (5−HT1b) receptor CHEMBL3459 pKi
Alpha−chymotrypsin CHEMBL3314 pKi
Cell division cycle 7−related protein kinase CHEMBL5443 pIC50
TNF−alpha CHEMBL1825 pIC50
Protein−tyrosine phosphatase 1B CHEMBL335 pIC50
Stem cell growth factor receptor CHEMBL1936 pIC50
C−C chemokine receptor type 4 CHEMBL2414 pIC50
Plasmepsin 1 CHEMBL4687 pIC50
Sigma opioid receptor CHEMBL3602 pIC50
Histamine H3 receptor CHEMBL3263 pIC50
Dopamine transporter CHEMBL338 pIC50
Histamine H1 receptor CHEMBL231 pIC50
Protein kinase C gamma CHEMBL2938 pIC50
5−lipoxygenase activating protein CHEMBL4550 pIC50
Bombesin receptor subtype−3 CHEMBL1075140 pEC50
DNA polymerase III CHEMBL3897 pIC50
Cytochrome P450 26A1 CHEMBL5141 pIC50
Vasopressin V2 receptor CHEMBL3766 pKi
Baculoviral IAP repeat−containing protein 2 CHEMBL5462 pIC50
erbB1; rv−QAFFP 440
Feature importance
0.00
0.05
0.10
0.15
0.20
Purinergic receptor P2Y12 CHEMBL2001 pKi
Leukotriene A4 hydrolase CHEMBL4618 pIC50
Protein−tyrosine phosphatase 1B CHEMBL335 pIC50
Histone deacetylase 8 CHEMBL3192 pIC50
Serotonin 1a (5−HT1a) receptor CHEMBL273 pIC50
Fructose−1,6−bisphosphatase CHEMBL3975 pIC50
Serotonin transporter CHEMBL228 pKi
Dihydrofolate reductase CHEMBL1075051 pIC50
Dual specificity protein kinase CLK4 CHEMBL4203 pIC50
Free fatty acid receptor 1 CHEMBL1795180 pKi
Dihydrofolate reductase CHEMBL5441 pIC50
NAD−dependent deacetylase sirtuin 2 CHEMBL4462 pIC50
Mu opioid receptor CHEMBL270 pEC50
Urokinase−type plasminogen activator CHEMBL3286 pKi
Neuropeptide Y receptor type 2 CHEMBL4018 pIC50
Dual specificity mitogen−activated protein kinase kinase 1 CHEMBL3587 pEC50
Serine/threonine−protein kinase PLK1 CHEMBL3024 pIC50
Human immunodeficiency virus type 1 reverse transcriptase CHEMBL247 pEC50
Sonic hedgehog protein CHEMBL5387 pIC50
Cytochrome P450 17A1 CHEMBL4430 pIC50
erbB1; b−QAFFP 440
Feature importance
0.00
0.05
0.10
0.15
Dihydrofolate reductase CHEMBL2425 pIC50
Cyclin−dependent kinase 4 CHEMBL331 pIC50
ADAM17 CHEMBL3706 pIC50
Carbonic anhydrase XIV CHEMBL3510 pKi
Urokinase−type plasminogen activator CHEMBL3286 pKi
Bcr/Abl fusion protein CHEMBL2096618 pIC50
Cytochrome P450 19A1 CHEMBL1978 pIC50
Acetylcholinesterase CHEMBL220 pKi
Beta−3 adrenergic receptor CHEMBL246 pEC50
Neuronal acetylcholine receptor protein alpha−7 subunit CHEMBL2492 pKi
Urokinase−type plasminogen activator CHEMBL3286 pIC50
Cholecystokinin A receptor CHEMBL2871 pKi
Mu opioid receptor CHEMBL233 pIC50
Dipeptidyl peptidase IX CHEMBL4793 pKi
Histone deacetylase 8 CHEMBL3192 pIC50
Carbonic anhydrase CHEMBL5337 pKi
Cyclin−dependent kinase 1/cyclin B CHEMBL2094127 pIC50
Matrix metalloproteinase 9 CHEMBL321 pIC50
Lysyl−tRNA synthetase CHEMBL5575 pIC50
Epidermal growth factor receptor erbB1 CHEMBL203 pIC50
Estrogen; rv−QAFFP 1360
Feature importance
0.00
0.05
0.10
0.15
Serotonin 4 (5−HT4) receptor CHEMBL1875 pKi
Vasopressin V1a receptor CHEMBL1889 pEC50
C−C chemokine receptor type 3 CHEMBL3473 pIC50
PI3−kinase p110−alpha subunit CHEMBL2499 pIC50
Neurokinin 1 receptor CHEMBL249 pIC50
LXR−alpha CHEMBL2808 pEC50
Prostaglandin E synthase CHEMBL5658 pIC50
Dual specificity mitogen−activated protein kinase kinase 1 CHEMBL3587 pEC50
Serine/threonine−protein kinase AKT2 CHEMBL2431 pIC50
Dopamine D1 receptor CHEMBL265 pKi
Dihydroorotate dehydrogenase CHEMBL3486 pIC50
Cytochrome P450 2C9 CHEMBL3397 pIC50
Melanocortin receptor 5 CHEMBL4608 pIC50
Caspase−3 CHEMBL2334 pIC50
Acetylcholinesterase CHEMBL4768 pIC50
Peroxisome proliferator−activated receptor alpha CHEMBL239 pIC50
Insulin−like growth factor I receptor CHEMBL1957 pIC50
Hypoxia−inducible factor 1 alpha CHEMBL4261 pIC50
Carbonic anhydrase I CHEMBL261 pIC50
Histamine H4 receptor CHEMBL3759 pKi
Estrogen; rv−QAFFP 440
Feature importance
0.00
0.05
0.10
0.15
0.20
Serotonin 4 (5−HT4) receptor CHEMBL1875 pKi
Peroxisome proliferator−activated receptor gamma CHEMBL235 pIC50
C−C chemokine receptor type 3 CHEMBL3473 pIC50
Fructose−1,6−bisphosphatase CHEMBL3975 pIC50
Dihydrofolate reductase CHEMBL5441 pKi
Serine/threonine−protein kinase AKT3 CHEMBL4816 pIC50
Carbonic anhydrase VB CHEMBL3969 pKi
Carbonic anhydrase IV CHEMBL3729 pKi
Leukocyte elastase CHEMBL248 pKi
Lysosomal Pro−X carboxypeptidase CHEMBL1255149 pIC50
Enoyl−[acyl−carrier−protein] reductase CHEMBL1849 pIC50
Alpha−1b adrenergic receptor CHEMBL232 pKi
ADAM17 CHEMBL3332 pIC50
Glutaminyl−peptide cyclotransferase CHEMBL4508 pIC50
Toll−like receptor 7 CHEMBL5936 pEC50
Neuronal acetylcholine receptor protein alpha−7 subunit CHEMBL2492 pEC50
Ghrelin receptor CHEMBL4616 pKi
ALK tyrosine kinase receptor CHEMBL4247 pIC50
Purinergic receptor P2Y12 CHEMBL2001 pIC50
Sodium/glucose cotransporter 1 CHEMBL4979 pIC50
Estrogen; b−QAFFP 440
Feature importance
0.000
0.025
0.050
0.075
Cyclin−dependent kinase 4 CHEMBL331 pIC50
Estrogen receptor alpha CHEMBL206 pIC50
Cholecystokinin A receptor CHEMBL3501 pKi
Glucocorticoid receptor CHEMBL2034 pKi
Seprase CHEMBL5769 pIC50
Peroxisome proliferator−activated receptor delta CHEMBL3979 pEC50
Cholecystokinin A receptor CHEMBL1901 pIC50
Cannabinoid CB2 receptor CHEMBL5373 pKi
11−beta−hydroxysteroid dehydrogenase 1 CHEMBL3910 pIC50
P2Y purinoceptor 1 CHEMBL5720 pIC50
Glucagon receptor CHEMBL1985 pIC50
Progesterone receptor CHEMBL208 pIC50
Serine/threonine−protein kinase Chk2 CHEMBL2527 pIC50
Carbonic anhydrase CHEMBL5337 pKi
DNA−dependent protein kinase CHEMBL3142 pIC50
Tyrosine−protein kinase ABL CHEMBL1862 pIC50
NAD−dependent deacetylase sirtuin 1 CHEMBL4506 pIC50
Cyclin−dependent kinase 2 CHEMBL301 pIC50
Cholecystokinin A receptor CHEMBL1901 pEC50
NAD−dependent deacetylase sirtuin 2 CHEMBL4462 pIC50

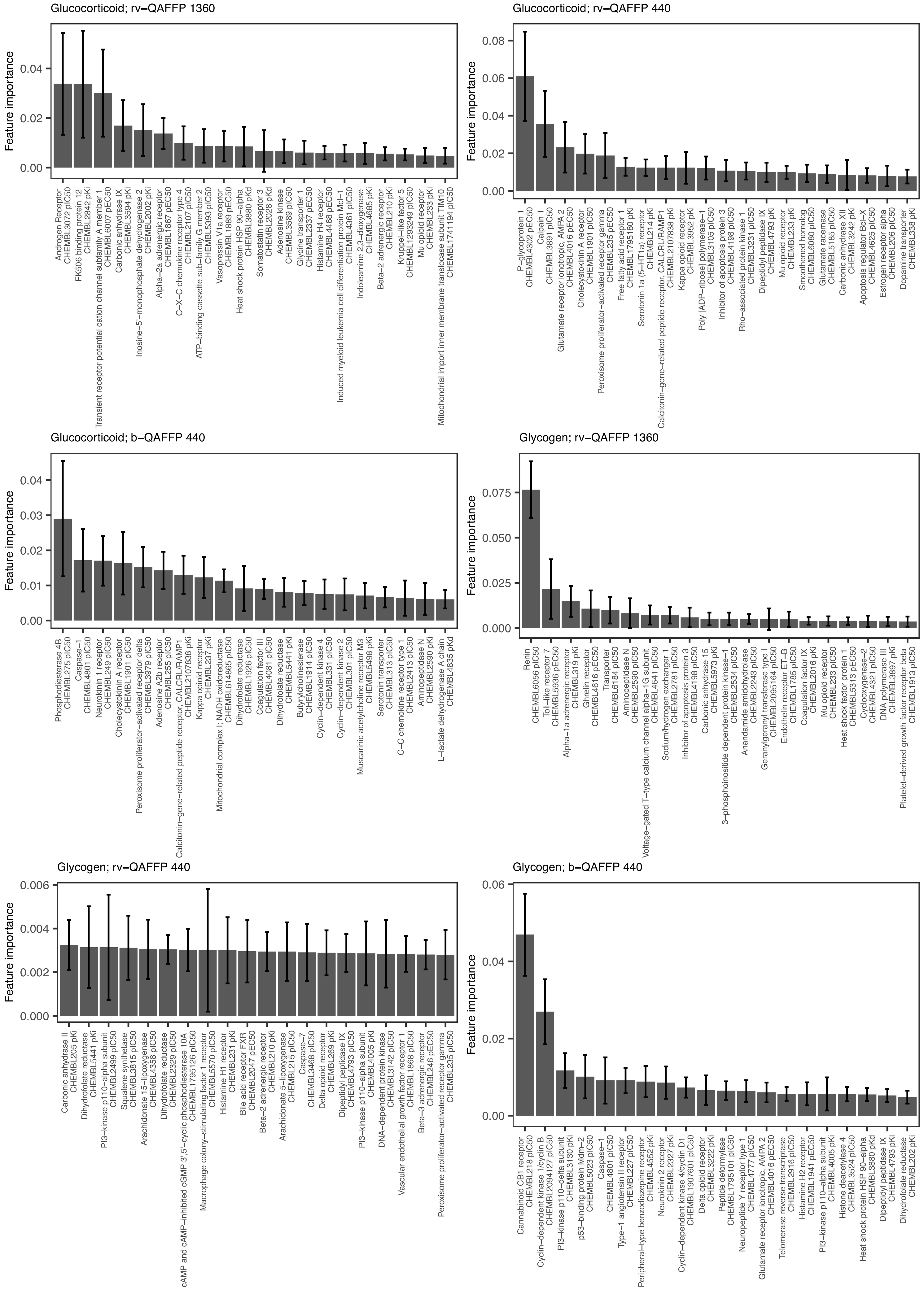
Glucocorticoid; rv−QAFFP 1360
Feature importance
0.00
0.02
0.04
Androgen Receptor CHEMBL3072 pIC50
FK506 binding protein 12 CHEMBL2842 pKi
Transient receptor potential cation channel subfamily A member 1 CHEMBL6007 pEC50
Carbonic anhydrase IX CHEMBL3594 pKi
Inosine−5'−monophosphate dehydrogenase 2 CHEMBL2002 pKi
Alpha−2a adrenergic receptor CHEMBL1867 pEC50
C−X−C chemokine receptor type 4 CHEMBL2107 pEC50
ATP−binding cassette sub−family G member 2 CHEMBL5393 pIC50
Vasopressin V1a receptor CHEMBL1889 pEC50
Heat shock protein HSP 90−alpha CHEMBL3880 pKd
Somatostatin receptor 3 CHEMBL2028 pKd
Adenosine kinase CHEMBL3589 pIC50
Glycine transporter 1 CHEMBL2337 pEC50
Histamine H4 receptor CHEMBL4468 pEC50
Induced myeloid leukemia cell differentiation protein Mcl−1 CHEMBL4361 pIC50
Indoleamine 2,3−dioxygenase CHEMBL4685 pKi
Beta−2 adrenergic receptor CHEMBL210 pKi
Kruppel−like factor 5 CHEMBL1293249 pIC50
Mu opioid receptor CHEMBL233 pKi
Mitochondrial import inner membrane translocase subunit TIM10 CHEMBL1741194 pIC50
Glucocorticoid; rv−QAFFP 440
Feature importance
0.00
0.02
0.04
0.06
0.08
P−glycoprotein 1 CHEMBL4302 pEC50
Calpain 1 CHEMBL3891 pIC50
Glutamate receptor ionotropic, AMPA 2 CHEMBL4016 pEC50
Cholecystokinin A receptor CHEMBL1901 pIC50
Peroxisome proliferator−activated receptor gamma CHEMBL235 pEC50
Free fatty acid receptor 1 CHEMBL1795180 pKi
Serotonin 1a (5−HT1a) receptor CHEMBL214 pKi
Calcitonin−gene−related peptide receptor, CALCRL/RAMP1 CHEMBL2107838 pKi
Kappa opioid receptor CHEMBL3952 pKi
Poly [ADP−ribose] polymerase−1 CHEMBL3105 pIC50
Inhibitor of apoptosis protein 3 CHEMBL4198 pIC50
Rho−associated protein kinase 1 CHEMBL3231 pIC50
Dipeptidyl peptidase IX CHEMBL4793 pKi
Mu opioid receptor CHEMBL233 pKi
Smoothened homolog CHEMBL6080 pIC50
Glutamate racemase CHEMBL5185 pIC50
Carbonic anhydrase XII CHEMBL3242 pKi
Apoptosis regulator Bcl−X CHEMBL4625 pIC50
Estrogen receptor alpha CHEMBL206 pIC50
Dopamine transporter CHEMBL338 pKi
Glucocorticoid; b−QAFFP 440
Feature importance
0.00
0.01
0.02
0.03
0.04
Phosphodiesterase 4B CHEMBL275 pIC50
Caspase−1 CHEMBL4801 pIC50
Neurokinin 1 receptor CHEMBL249 pIC50
Cholecystokinin A receptor CHEMBL1901 pIC50
Peroxisome proliferator−activated receptor delta CHEMBL3979 pIC50
Adenosine A2b receptor CHEMBL255 pIC50
Calcitonin−gene−related peptide receptor, CALCRL/RAMP1 CHEMBL2107838 pIC50
Kappa opioid receptor CHEMBL237 pKi
Mitochondrial complex I; NADH oxidoreductase CHEMBL614865 pIC50
Dihydrofolate reductase CHEMBL1926 pIC50
Coagulation factor III CHEMBL4081 pIC50
Dihydrofolate reductase CHEMBL5441 pKi
Butyrylcholinesterase CHEMBL1914 pIC50
Cyclin−dependent kinase 4 CHEMBL331 pIC50
Cyclin−dependent kinase 2 CHEMBL301 pIC50
Muscarinic acetylcholine receptor M3 CHEMBL5498 pKi
Serotonin transporter CHEMBL313 pIC50
C−C chemokine receptor type 1 CHEMBL2413 pIC50
Aminopeptidase N CHEMBL2590 pKi
L−lactate dehydrogenase A chain CHEMBL4835 pKd
Glycogen; rv−QAFFP 1360
Feature importance
0.000
0.025
0.050
0.075
Renin CHEMBL6056 pIC50
Toll−like receptor 7 CHEMBL5936 pEC50
Alpha−1a adrenergic receptor CHEMBL319 pKi
Ghrelin receptor CHEMBL4616 pEC50
Transporter CHEMBL6184 pIC50
Aminopeptidase N CHEMBL2590 pIC50
Voltage−gated T−type calcium channel alpha−1G subunit CHEMBL4641 pIC50
Sodium/hydrogen exchanger 1 CHEMBL2781 pIC50
Inhibitor of apoptosis protein 3 CHEMBL4198 pIC50
Carbonic anhydrase 15 CHEMBL5973 pKi
3−phosphoinositide dependent protein kinase−1 CHEMBL2534 pIC50
Anandamide amidohydrolase CHEMBL2243 pIC50
Geranylgeranyl transferase type I CHEMBL2095164 pIC50
Endothelin receptor ET−B CHEMBL1785 pIC50
Coagulation factor IX CHEMBL2016 pKi
Mu opioid receptor CHEMBL233 pIC50
Heat shock factor protein 1 CHEMBL5313 pEC50
Cyclooxygenase−2 CHEMBL4321 pIC50
DNA polymerase III CHEMBL3897 pKi
Platelet−derived growth factor receptor beta CHEMBL1913 pIC50
Glycogen; rv−QAFFP 440
Feature importance
0.000
0.002
0.004
0.006
Carbonic anhydrase II CHEMBL205 pKi
Dihydrofolate reductase CHEMBL5441 pKi
PI3−kinase p110−alpha subunit CHEMBL2499 pIC50
Squalene synthetase CHEMBL3815 pIC50
Arachidonate 15−lipoxygenase CHEMBL4358 pIC50
Dihydrofolate reductase CHEMBL2329 pIC50
cAMP and cAMP−inhibited cGMP 3',5'−cyclic phosphodiesterase 10A CHEMBL1795126 pIC50
Macrophage colony−stimulating factor 1 receptor CHEMBL5570 pIC50
Histamine H1 receptor CHEMBL231 pKi
Bile acid receptor FXR CHEMBL2047 pEC50
Beta−2 adrenergic receptor CHEMBL210 pKi
Arachidonate 5−lipoxygenase CHEMBL215 pIC50
Caspase−7 CHEMBL3468 pIC50
Delta opioid receptor CHEMBL269 pKi
Dipeptidyl peptidase IX CHEMBL4793 pIC50
PI3−kinase p110−alpha subunit CHEMBL4005 pKi
DNA−dependent protein kinase CHEMBL3142 pIC50
Vascular endothelial growth factor receptor 1 CHEMBL1868 pIC50
Beta−3 adrenergic receptor CHEMBL246 pEC50
Peroxisome proliferator−activated receptor gamma CHEMBL235 pIC50
Glycogen; b−QAFFP 440
Feature importance
0.00
0.02
0.04
0.06
Cannabinoid CB1 receptor CHEMBL218 pIC50
Cyclin−dependent kinase 1/cyclin B CHEMBL2094127 pIC50
PI3−kinase p110−delta subunit CHEMBL3130 pKi
p53−binding protein Mdm−2 CHEMBL5023 pIC50
Caspase−1 CHEMBL4801 pIC50
Type−1 angiotensin II receptor CHEMBL227 pIC50
Peripheral−type benzodiazepine receptor CHEMBL4552 pKi
Neurokinin 2 receptor CHEMBL2327 pKi
Cyclin−dependent kinase 4/cyclin D1 CHEMBL1907601 pIC50
Delta opioid receptor CHEMBL3222 pKi
Peptide deformylase CHEMBL1795101 pIC50
Neuropeptide Y receptor type 1 CHEMBL4777 pIC50
Glutamate receptor ionotropic, AMPA 2 CHEMBL4016 pEC50
Telomerase reverse transcriptase CHEMBL2916 pIC50
Histamine H2 receptor CHEMBL1941 pEC50
PI3−kinase p110−alpha subunit CHEMBL4005 pIC50
Histone deacetylase 4 CHEMBL3524 pIC50
Heat shock protein HSP 90−alpha CHEMBL3880 pKd
Dipeptidyl peptidase IX CHEMBL4793 pKi
Dihydrofolate reductase CHEMBL202 pKi

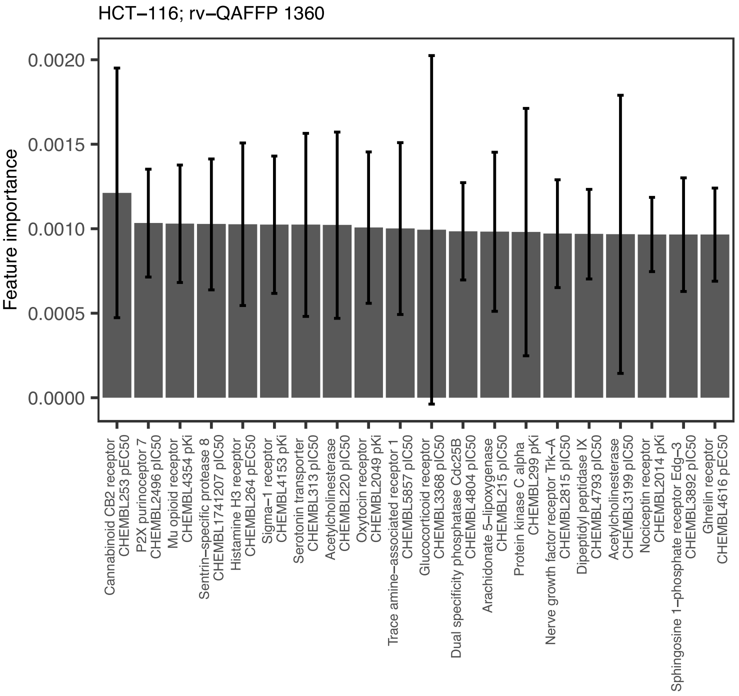
HCT−116; rv−QAFFP 1360
Feature importance
0.0020
0.0015
0.0010
0.0005
0.0000
Cannabinoid CB2 receptor CHEMBL253 pEC50
P2X purinoceptor 7 CHEMBL2496 pIC50
Mu opioid receptor CHEMBL4354 pKi
Sentrin−specific protease 8 CHEMBL1741207 pIC50
Histamine H3 receptor CHEMBL264 pEC50
Sigma−1 receptor CHEMBL4153 pKi
Serotonin transporter CHEMBL313 pIC50
Acetylcholinesterase CHEMBL220 pIC50
Oxytocin receptor CHEMBL2049 pKi
Trace amine−associated receptor 1 CHEMBL5857 pIC50
Glucocorticoid receptor CHEMBL3368 pIC50
Dual specificity phosphatase Cdc25B CHEMBL4804 pIC50
Arachidonate 5−lipoxygenase CHEMBL215 pIC50
Protein kinase C alpha CHEMBL299 pKi
Nerve growth factor receptor Trk−A CHEMBL2815 pIC50
Dipeptidyl peptidase IX CHEMBL4793 pIC50
Acetylcholinesterase CHEMBL3199 pIC50
Nociceptin receptor CHEMBL2014 pKi
Sphingosine 1−phosphate receptor Edg−3 CHEMBL3892 pIC50
Ghrelin receptor CHEMBL4616 pEC50

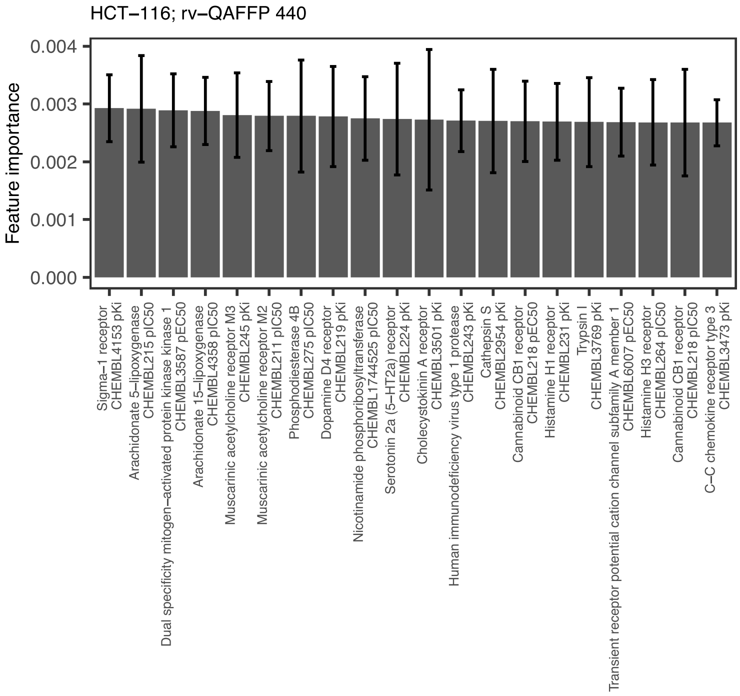
HCT−116; rv−QAFFP 440
Feature importance
0.004
0.003
0.002
0.001
0.000
Sigma−1 receptor CHEMBL4153 pKi
Arachidonate 5−lipoxygenase CHEMBL215 pIC50
Dual specificity mitogen−activated protein kinase kinase 1 CHEMBL3587 pEC50
Arachidonate 15−lipoxygenase CHEMBL4358 pIC50
Muscarinic acetylcholine receptor M3 CHEMBL245 pKi
Muscarinic acetylcholine receptor M2 CHEMBL211 pIC50
Phosphodiesterase 4B CHEMBL275 pIC50
Dopamine D4 receptor CHEMBL219 pKi
Nicotinamide phosphoribosyltransferase CHEMBL1744525 pIC50
Serotonin 2a (5−HT2a) receptor CHEMBL224 pKi
Cholecystokinin A receptor CHEMBL3501 pKi
Human immunodeficiency virus type 1 protease CHEMBL243 pKi
Cathepsin S CHEMBL2954 pKi
Cannabinoid CB1 receptor CHEMBL218 pEC50
Histamine H1 receptor CHEMBL231 pKi
Trypsin I CHEMBL3769 pKi
Transient receptor potential cation channel subfamily A member 1 CHEMBL6007 pEC50
Histamine H3 receptor CHEMBL264 pKi
Cannabinoid CB1 receptor CHEMBL218 pIC50
C−C chemokine receptor type 3 CHEMBL3473 pKi

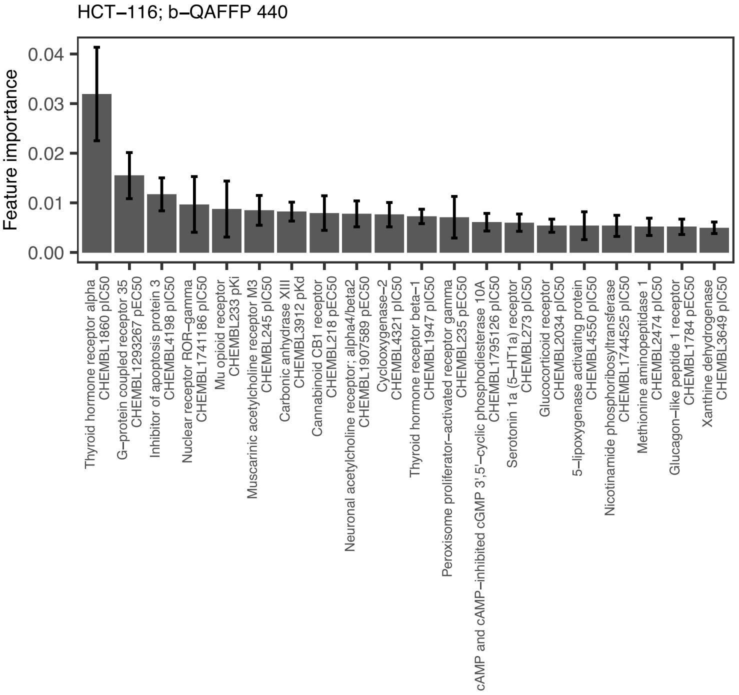
HCT−116; b−QAFFP 440
Feature importance
0.04
0.03
0.02
0.01
0.00
Thyroid hormone receptor alpha CHEMBL1860 pIC50
G−protein coupled receptor 35 CHEMBL1293267 pEC50
Inhibitor of apoptosis protein 3 CHEMBL4198 pIC50
Nuclear receptor ROR−gamma CHEMBL1741186 pIC50
Mu opioid receptor CHEMBL233 pKi
Muscarinic acetylcholine receptor M3 CHEMBL245 pIC50
Carbonic anhydrase XIII CHEMBL3912 pKd
Cannabinoid CB1 receptor CHEMBL218 pEC50
Neuronal acetylcholine receptor; alpha4/beta2 CHEMBL1907589 pEC50
Cyclooxygenase−2 CHEMBL4321 pIC50
Thyroid hormone receptor beta−1 CHEMBL1947 pIC50
Peroxisome proliferator−activated receptor gamma CHEMBL235 pEC50
cAMP and cAMP−inhibited cGMP 3',5'−cyclic phosphodiesterase 10A CHEMBL1795126 pIC50
Serotonin 1a (5−HT1a) receptor CHEMBL273 pIC50
Glucocorticoid receptor CHEMBL2034 pIC50
5−lipoxygenase activating protein CHEMBL4550 pIC50
Nicotinamide phosphoribosyltransferase CHEMBL1744525 pIC50
Methionine aminopeptidase 1 CHEMBL2474 pIC50
Glucagon−like peptide 1 receptor CHEMBL1784 pEC50
Xanthine dehydrogenase CHEMBL3649 pIC50

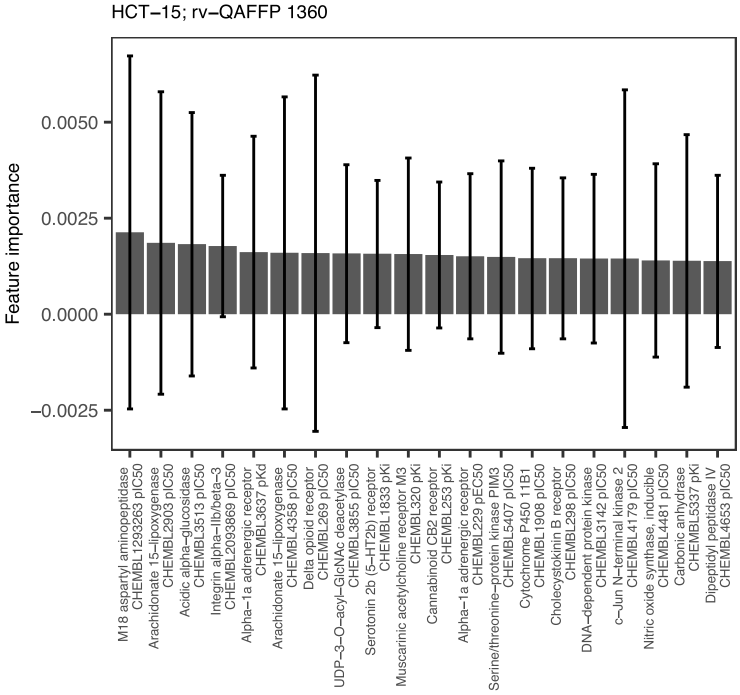
HCT−15; rv−QAFFP 1360
Feature importance
0.0050
0.0025
0.0000
−0.0025
M18 aspartyl aminopeptidase CHEMBL1293263 pIC50
Arachidonate 15−lipoxygenase CHEMBL2903 pIC50
Acidic alpha−glucosidase CHEMBL3513 pIC50
Integrin alpha−IIb/beta−3 CHEMBL2093869 pIC50
Alpha−1a adrenergic receptor CHEMBL3637 pKd
Arachidonate 15−lipoxygenase CHEMBL4358 pIC50
Delta opioid receptor CHEMBL269 pIC50
UDP−3−O−acyl−GlcNAc deacetylase CHEMBL3855 pIC50
Serotonin 2b (5−HT2b) receptor CHEMBL1833 pKi
Muscarinic acetylcholine receptor M3 CHEMBL320 pKi
Cannabinoid CB2 receptor CHEMBL253 pKi
Alpha−1a adrenergic receptor CHEMBL229 pEC50
Serine/threonine−protein kinase PIM3 CHEMBL5407 pIC50
Cytochrome P450 11B1 CHEMBL1908 pIC50
Cholecystokinin B receptor CHEMBL298 pIC50
DNA−dependent protein kinase CHEMBL3142 pIC50
c−Jun N−terminal kinase 2 CHEMBL4179 pIC50
Nitric oxide synthase, inducible CHEMBL4481 pIC50
Carbonic anhydrase CHEMBL5337 pKi
Dipeptidyl peptidase IV CHEMBL4653 pIC50

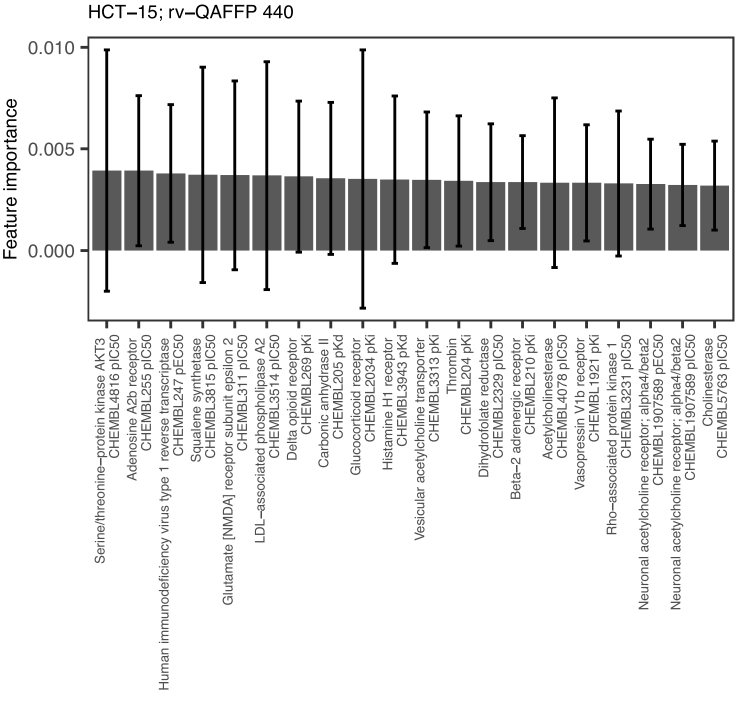
HCT−15; rv−QAFFP 440
Feature importance
0.010
0.005
0.000
Serine/threonine−protein kinase AKT3 CHEMBL4816 pIC50
Adenosine A2b receptor CHEMBL255 pIC50
Human immunodeficiency virus type 1 integrase CHEMBL247 pEC50
Squalene synthetase CHEMBL3815 pIC50
Glutamate [NMDA] receptor subunit epsilon 2 CHEMBL311 pIC50
LDL−associated phospholipase A2 CHEMBL3514 pIC50
Delta opioid receptor CHEMBL269 pKi
Carbonic anhydrase II CHEMBL205 pKd
Glucocorticoid receptor CHEMBL2034 pKi
Histamine H1 receptor CHEMBL3943 pKd
Vesicular acetylcholine transporter CHEMBL3313 pKi
Thrombin CHEMBL204 pKi
Dihydrofolate reductase CHEMBL2329 pIC50
Beta−2 adrenergic receptor CHEMBL210 pKi
Acetylcholinesterase CHEMBL4078 pIC50
Vasopressin V1b receptor CHEMBL1921 pKi
Rho−associated protein kinase 1 CHEMBL3231 pIC50
Neuronal acetylcholine receptor; alpha4/beta2 CHEMBL1907589 pEC50
Neuronal acetylcholine receptor; alpha4/beta2 CHEMBL1907589 pIC50
Cholinesterase CHEMBL5763 pIC50

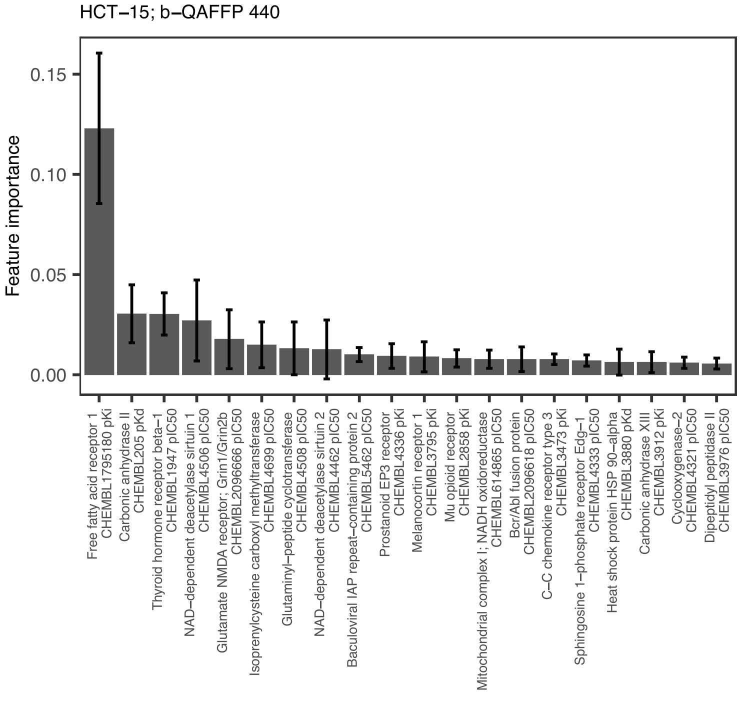
HCT−15; b−QAFFP 440
Feature importance
0.15
0.10
0.05
0.00
Free fatty acid receptor 1 CHEMBL1795180 pKi
Carbonic anhydrase II CHEMBL205 pKd
Thyroid hormone receptor beta−1 CHEMBL1947 pIC50
NAD−dependent deacetylase sirtuin 1 CHEMBL4506 pIC50
Glutamate NMDA receptor; Grin1/Grin2b CHEMBL2096666 pIC50
Isoprenylcysteine carboxyl methyltransferase CHEMBL4699 pIC50
Glutaminyl−peptide cyclotransferase CHEMBL4508 pIC50
NAD−dependent deacetylase sirtuin 2 CHEMBL4462 pIC50
Baculoviral IAP repeat−containing protein 2 CHEMBL5462 pIC50
Prostanoid EP3 receptor CHEMBL4336 pKi
Melanocortin receptor 1 CHEMBL3795 pIC50
Mu opioid receptor CHEMBL2858 pKi
Mitochondrial complex I; NADH oxidoreductase CHEMBL614865 pIC50
Bcr/Abl fusion protein CHEMBL2096618 pIC50
C−C chemokine receptor type 3 CHEMBL3473 pKi
Sphingosine 1−phosphate receptor Edg−1 CHEMBL4333 pIC50
Heat shock protein HSP 90−alpha CHEMBL3880 pKd
Carbonic anhydrase XIII CHEMBL3912 pKi
Cyclooxygenase−2 CHEMBL4321 pIC50
Dipeptidyl peptidase II CHEMBL3976 pIC50

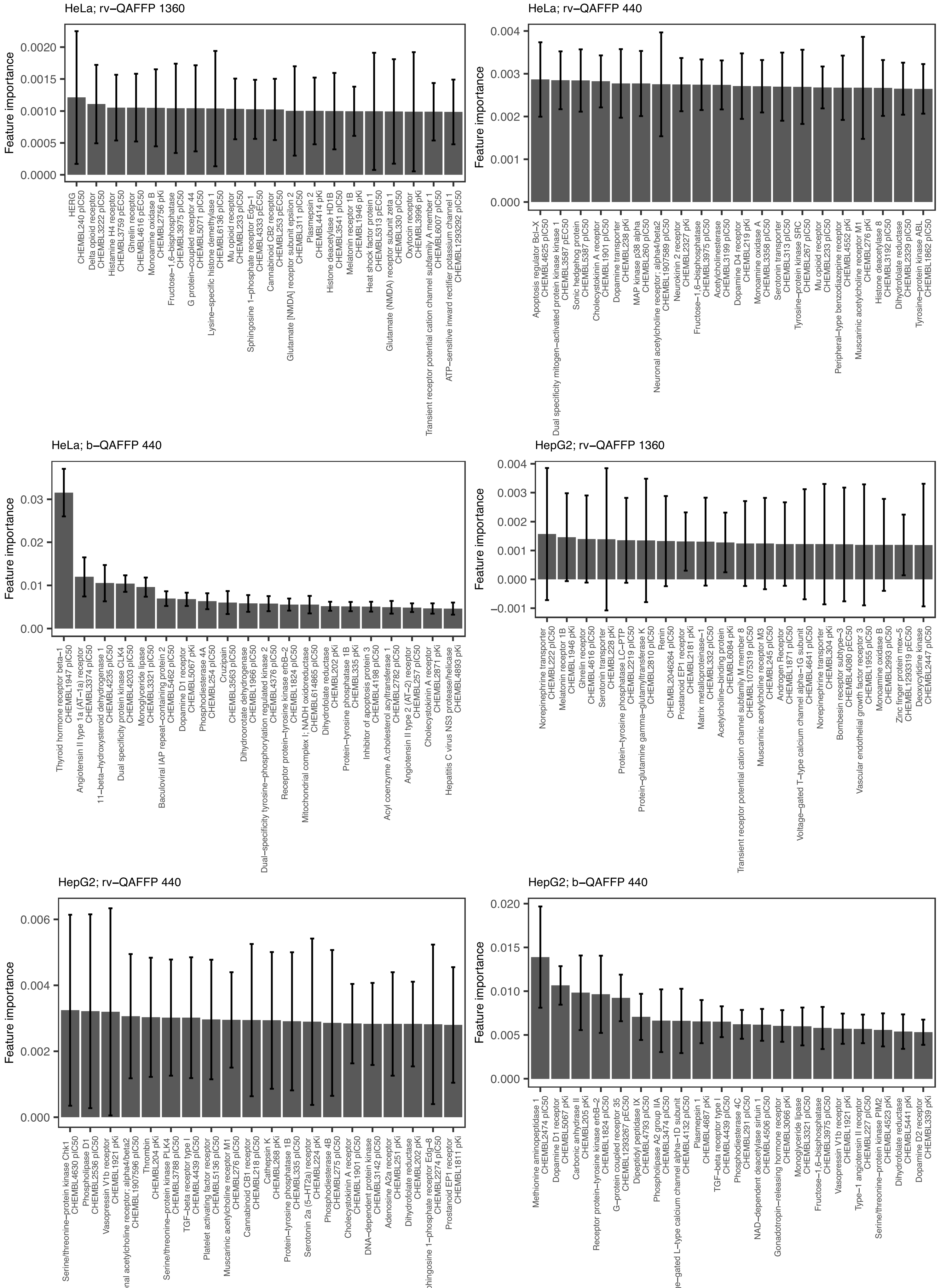
HeLa; rv–QAFFP 1360
Feature importance
0.0000
0.0005
0.0010
0.0015
0.0020
HERG CHEMBL240 pIC50
Delta opioid receptor CHEMBL3222 pIC50
Histamine H4 receptor CHEMBL3759 pEC50
Ghrelin receptor CHEMBL4616 pEC50
Monoamine oxidase B CHEMBL2756 pKi
Fructose–1,6–bisphosphatase CHEMBL3975 pIC50
G protein–coupled receptor 44 CHEMBL5071 pIC50
Lysine–specific histone demethylase 1 CHEMBL6136 pIC50
Mu opioid receptor CHEMBL233 pIC50
Sphingosine 1–phosphate receptor Edg–1 CHEMBL4333 pEC50
Cannabinoid CB2 receptor CHEMBL253 pEC50
Glutamate [NMDA] receptor subunit epsilon 2 CHEMBL311 pIC50
Plasmepsin 2 CHEMBL4414 pKi
Histone deacetylase HD1B CHEMBL3541 pIC50
Melatonin receptor 1B CHEMBL1946 pKi
Heat shock factor protein 1 CHEMBL5313 pEC50
Glutamate (NMDA) receptor subunit zeta 1 CHEMBL330 pIC50
Oxytocin receptor CHEMBL3996 pKi
Transient receptor potential cation channel subfamily A member 1 CHEMBL6007 pIC50
ATP–sensitive inward rectifier potassium channel 1 CHEMBL1293292 pIC50
HeLa; rv–QAFFP 440
Feature importance
0.000
0.001
0.002
0.003
0.004
Apoptosis regulator Bcl–X CHEMBL4625 pIC50
Dual specificity mitogen–activated protein kinase kinase 1 CHEMBL3587 pEC50
Sonic hedgehog protein CHEMBL5387 pIC50
Cholecystokinin A receptor CHEMBL1901 pIC50
Dopamine transporter CHEMBL238 pKi
MAP kinase p38 alpha CHEMBL260 pIC50
Neuronal acetylcholine receptor; alpha4/beta2 CHEMBL1907589 pIC50
Neurokinin 2 receptor CHEMBL2327 pKi
Fructose–1,6–bisphosphatase CHEMBL3975 pIC50
Acetylcholinesterase CHEMBL3199 pIC50
Dopamine D4 receptor CHEMBL219 pKi
Monoamine oxidase A CHEMBL3358 pIC50
Serotonin transporter CHEMBL313 pIC50
Tyrosine–protein kinase SRC CHEMBL267 pIC50
Mu opioid receptor CHEMBL233 pIC50
Peripheral–type benzodiazepine receptor CHEMBL4552 pKi
Muscarinic acetylcholine receptor M1 CHEMBL276 pKi
Histone deacetylase 8 CHEMBL3192 pIC50
Dihydrofolate reductase CHEMBL2329 pIC50
Tyrosine–protein kinase ABL CHEMBL1862 pIC50
HeLa; b–QAFFP 440
Feature importance
0.00
0.01
0.02
0.03
Thyroid hormone receptor beta–1 CHEMBL1947 pIC50
Angiotensin II type 1a (AT–1a) receptor CHEMBL3374 pIC50
11–beta–hydroxysteroid dehydrogenase 1 CHEMBL4235 pIC50
Dual specificity protein kinase CLK4 CHEMBL4203 pIC50
Monoglyceride lipase CHEMBL3321 pIC50
Baculoviral IAP repeat–containing protein 2 CHEMBL5462 pIC50
Dopamine D1 receptor CHEMBL5067 pKi
Phosphodiesterase 4A CHEMBL254 pIC50
Cruzipain CHEMBL3563 pIC50
Dihydroorotate dehydrogenase CHEMBL1966 pIC50
Dual–specificity tyrosine–phosphorylation regulated kinase 2 CHEMBL4376 pIC50
Receptor protein–tyrosine kinase erbB–2 CHEMBL1824 pIC50
Mitochondrial complex I; NADH oxidoreductase CHEMBL614865 pIC50
Dihydrofolate reductase CHEMBL202 pKi
Protein–tyrosine phosphatase 1B CHEMBL335 pKi
Inhibitor of apoptosis protein 3 CHEMBL4198 pIC50
Acyl coenzyme A:cholesterol acyltransferase 1 CHEMBL2782 pIC50
Angiotensin II type 2 (AT–2) receptor CHEMBL257 pIC50
Cholecystokinin A receptor CHEMBL2871 pIC50
Hepatitis C virus NS3 protease/helicase CHEMBL4893 pKi
HepG2; rv–QAFFP 1360
Feature importance
−0.001
0.000
0.001
0.002
0.003
0.004
Norepinephrine transporter CHEMBL222 pIC50
Melatonin receptor 1B CHEMBL1946 pKi
Ghrelin receptor CHEMBL4616 pIC50
Serotonin transporter CHEMBL228 pKi
Protein–tyrosine phosphatase LC–PTP CHEMBL2219 pIC50
Protein–glutamine gamma–glutamyltransferase K CHEMBL2810 pIC50
Renin CHEMBL2046264 pIC50
Prostanoid EP1 receptor CHEMBL2181 pKi
Matrix metalloproteinase–1 CHEMBL332 pIC50
Acetylcholine–binding protein CHEMBL6084 pKi
Transient receptor potential cation channel subfamily M member 8 CHEMBL1075319 pIC50
Muscarinic acetylcholine receptor M3 CHEMBL245 pIC50
Androgen Receptor CHEMBL1871 pIC50
Voltage–gated T–type calcium channel alpha–1G subunit CHEMBL4641 pIC50
Norepinephrine transporter CHEMBL304 pKi
Bombesin receptor subtype–3 CHEMBL4080 pEC50
Vascular endothelial growth factor receptor 3 CHEMBL1955 pIC50
Monoamine oxidase B CHEMBL2993 pIC50
Zinc finger protein mex–5 CHEMBL1293319 pEC50
Deoxycytidine kinase CHEMBL2447 pIC50
HepG2; rv–QAFFP 440
Feature importance
0.000
0.002
0.004
0.006
Serine/threonine–protein kinase Chk1 CHEMBL4630 pIC50
Phospholipase D1 CHEMBL2536 pIC50
Vasopressin V1b receptor CHEMBL1921 pKi
Neuronal acetylcholine receptor; alpha4/beta2 CHEMBL1907596 pIC50
Thrombin CHEMBL204 pKi
Serine/threonine–protein kinase PLK4 CHEMBL3788 pIC50
TGF–beta receptor type I CHEMBL4439 pIC50
Platelet activating factor receptor CHEMBL5136 pIC50
Muscarinic acetylcholine receptor M1 CHEMBL276 pIC50
Cannabinoid CB1 receptor CHEMBL218 pIC50
Cathepsin K CHEMBL268 pKi
Protein–tyrosine phosphatase 1B CHEMBL335 pIC50
Serotonin 2a (5–HT2a) receptor CHEMBL224 pKi
Phosphodiesterase 4B CHEMBL275 pIC50
Cholecystokinin A receptor CHEMBL1901 pIC50
DNA–dependent protein kinase CHEMBL3142 pIC50
Adenosine A2a receptor CHEMBL251 pIC50
Dihydrofolate reductase CHEMBL202 pKi
Sphingosine 1–phosphate receptor Edg–8 CHEMBL2274 pIC50
Prostanoid EP1 receptor CHEMBL1811 pKi
HepG2; b–QAFFP 440
Feature importance
0.000
0.005
0.010
0.015
0.020
Methionine aminopeptidase 1 CHEMBL2474 pIC50
Dopamine D1 receptor CHEMBL5067 pKi
Carbonic anhydrase II CHEMBL205 pKi
Receptor protein–tyrosine kinase erbB–2 CHEMBL1824 pIC50
G–protein coupled receptor 35 CHEMBL1293267 pEC50
Dipeptidyl peptidase IX CHEMBL4793 pIC50
Phospholipase A2 group IIA CHEMBL3474 pIC50
Voltage–gated L–type calcium channel alpha–1D subunit CHEMBL4132 pIC50
Plasmepsin 1 CHEMBL4687 pKi
TGF–beta receptor type I CHEMBL4439 pIC50
Phosphodiesterase 4C CHEMBL291 pIC50
NAD–dependent deacetylase sirtuin 1 CHEMBL4506 pIC50
Gonadotropin–releasing hormone receptor CHEMBL3066 pKi
Monoglyceride lipase CHEMBL3321 pIC50
Fructose–1,6–bisphosphatase CHEMBL3975 pIC50
Vasopressin V1b receptor CHEMBL1921 pKi
Type–1 angiotensin II receptor CHEMBL227 pIC50
Serine/threonine–protein kinase PIM2 CHEMBL4523 pKi
Dihydrofolate reductase CHEMBL5441 pKi
Dopamine D2 receptor CHEMBL339 pKi

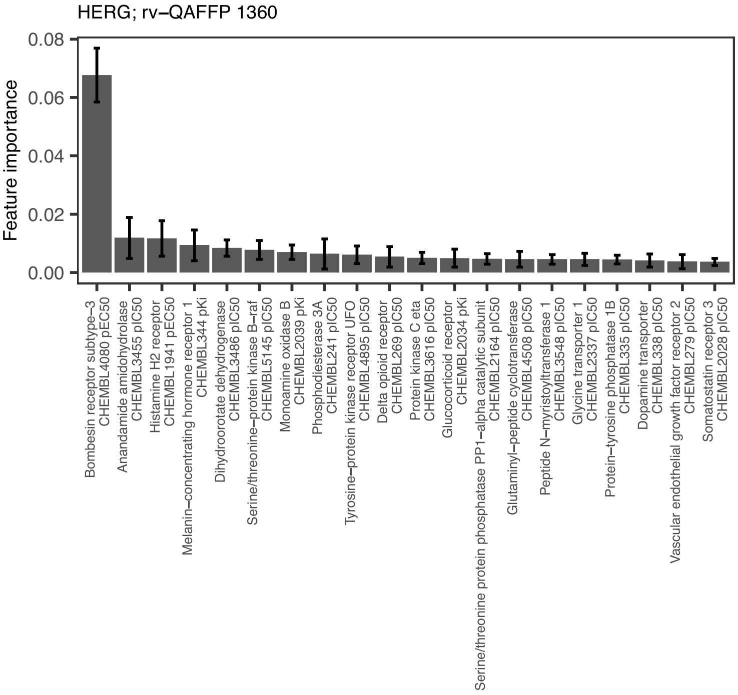
HERG; rv–QAFFP 1360
Feature importance
0.00
0.02
0.04
0.06
0.08
Bombesin receptor subtype–3 CHEMBL4080 pEC50
Anandamide amidohydrolase CHEMBL3455 pIC50
Histamine H2 receptor CHEMBL1941 pEC50
Melanin–concentrating hormone receptor 1 CHEMBL344 pKi
Dihydroorotate dehydrogenase CHEMBL3486 pIC50
Serine/threonine–protein kinase B–raf CHEMBL5145 pIC50
Monoamine oxidase B CHEMBL2039 pKi
Phosphodiesterase 3A CHEMBL241 pIC50
Tyrosine–protein kinase receptor UFO CHEMBL4895 pIC50
Delta opioid receptor CHEMBL269 pIC50
Protein kinase C eta CHEMBL3616 pIC50
Glucocorticoid receptor CHEMBL2034 pKi
Serine/threonine protein phosphatase PP1–alpha catalytic subunit CHEMBL2164 pIC50
Glutaminyl–peptide cyclotransferase CHEMBL4508 pIC50
Peptide N–myristoyltransferase 1 CHEMBL3548 pIC50
Glycine transporter 1 CHEMBL2337 pIC50
Protein–tyrosine phosphatase 1B CHEMBL335 pIC50
Dopamine transporter CHEMBL338 pIC50
Vascular endothelial growth factor receptor 2 CHEMBL279 pIC50
Somatostatin receptor 3 CHEMBL2028 pIC50

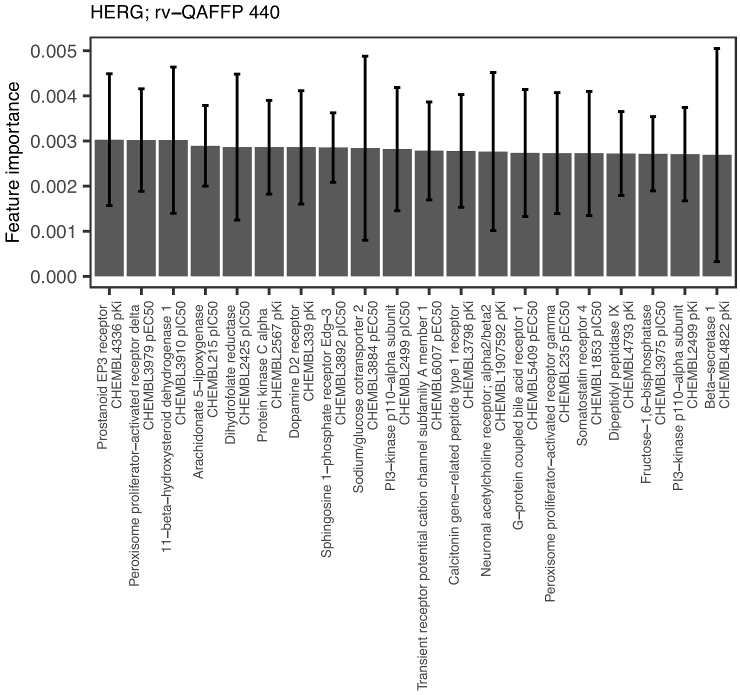
HERG; rv–QAFFP 440
Feature importance
0.000
0.001
0.002
0.003
0.004
0.005
Prostanoid EP3 receptor CHEMBL4336 pKi
Peroxisome proliferator–activated receptor delta CHEMBL3979 pEC50
11–beta–hydroxysteroid dehydrogenase 1 CHEMBL3910 pIC50
Arachidonate 5–lipoxygenase CHEMBL215 pIC50
Dihydrofolate reductase CHEMBL2425 pIC50
Protein kinase C alpha CHEMBL2567 pKi
Dopamine D2 receptor CHEMBL339 pKi
Sphingosine 1–phosphate receptor Edg–3 CHEMBL3892 pIC50
Sodium/glucose cotransporter 2 CHEMBL3884 pEC50
PI3–kinase p110–alpha subunit CHEMBL2499 pIC50
Transient receptor potential cation channel subfamily A member 1 CHEMBL6007 pEC50
Calcitonin gene–related peptide type 1 receptor CHEMBL3798 pIC50
Neuronal acetylcholine receptor; alpha2/beta2 CHEMBL1907592 pKi
G–protein coupled bile acid receptor 1 CHEMBL5409 pEC50
Peroxisome proliferator–activated receptor gamma CHEMBL235 pEC50
Somatostatin receptor 4 CHEMBL1853 pIC50
Dipeptidyl peptidase IX CHEMBL4793 pKi
Fructose–1,6–bisphosphatase CHEMBL3975 pIC50
PI3–kinase p110–alpha subunit CHEMBL2499 pKi
Beta–secretase 1 CHEMBL4822 pKi

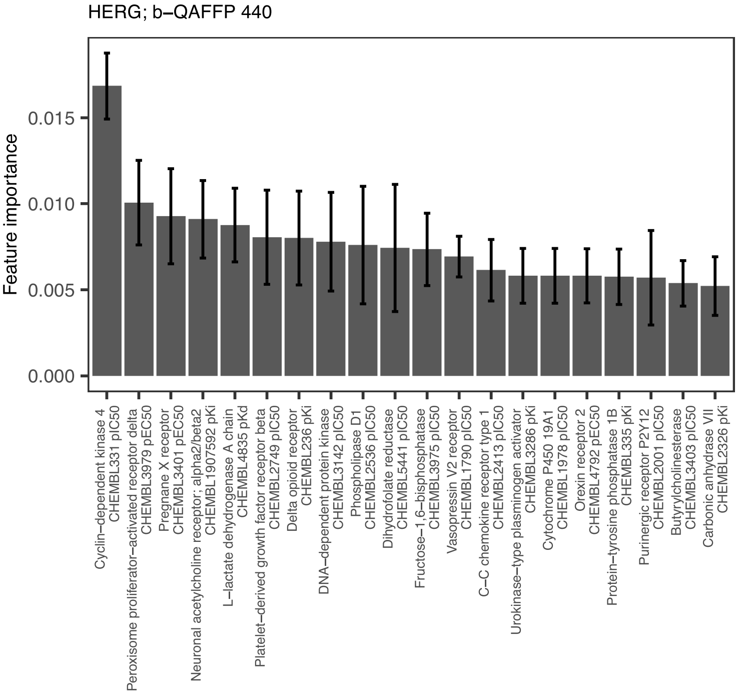
HERG; b–QAFFP 440
Feature importance
0.000
0.005
0.010
0.015
Cyclin–dependent kinase 4 CHEMBL331 pIC50
Peroxisome proliferator–activated receptor delta CHEMBL3979 pEC50
Pregnane X receptor CHEMBL3401 pEC50
Neuronal acetylcholine receptor; alpha2/beta2 CHEMBL1907592 pKi
L–lactate dehydrogenase A chain CHEMBL4835 pKd
Platelet–derived growth factor receptor beta CHEMBL2749 pIC50
Delta opioid receptor CHEMBL236 pKi
DNA–dependent protein kinase CHEMBL3142 pIC50
Phospholipase D1 CHEMBL2536 pIC50
Dihydrofolate reductase CHEMBL5441 pIC50
Fructose–1,6–bisphosphatase CHEMBL3975 pIC50
Vasopressin V2 receptor CHEMBL1790 pIC50
C–C chemokine receptor type 1 CHEMBL2413 pIC50
Urokinase–type plasminogen activator CHEMBL3286 pKi
Cytochrome P450 19A1 CHEMBL1978 pIC50
Orexin receptor 2 CHEMBL4792 pEC50
Protein–tyrosine phosphatase 1B CHEMBL335 pKi
Purinergic receptor P2Y12 CHEMBL2001 pIC50
Butyrylcholinesterase CHEMBL3403 pIC50
Carbonic anhydrase VII CHEMBL2326 pKi

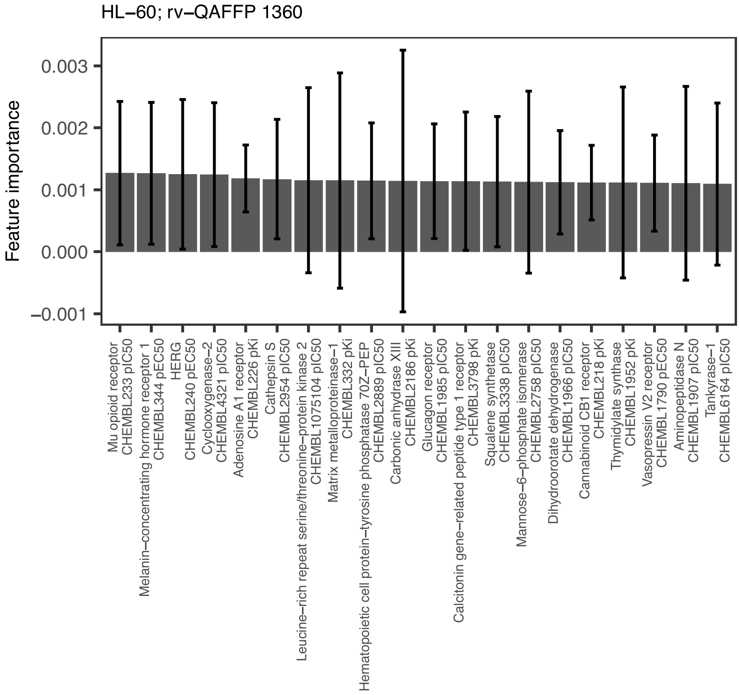
HL–60; rv–QAFFP 1360
Feature importance
−0.001
0.000
0.001
0.002
0.003
Mu opioid receptor CHEMBL233 pIC50
Melanin–concentrating hormone receptor 1 CHEMBL344 pEC50
HERG CHEMBL240 pEC50
Cyclooxygenase–2 CHEMBL4321 pIC50
Adenosine A1 receptor CHEMBL226 pKi
Cathepsin S CHEMBL2954 pIC50
Leucine–rich repeat serine/threonine–protein kinase 2 CHEMBL1075104 pIC50
Matrix metalloproteinase–1 CHEMBL332 pKi
Hematopoietic cell protein–tyrosine phosphatase 70Z–PEP CHEMBL2889 pEC50
Carbonic anhydrase XIII CHEMBL2186 pKi
Glucagon receptor CHEMBL1985 pIC50
Calcitonin gene–related peptide type 1 receptor CHEMBL3798 pKi
Squalene synthetase CHEMBL3338 pIC50
Mannose–6–phosphate isomerase CHEMBL2758 pIC50
Dihydroorotate dehydrogenase CHEMBL1966 pIC50
Cannabinoid CB1 receptor CHEMBL218 pKi
Thymidylate synthase CHEMBL1952 pKi
Vasopressin V2 receptor CHEMBL1790 pEC50
Aminopeptidase N CHEMBL1907 pIC50
Tankyrase–1 CHEMBL6164 pIC50

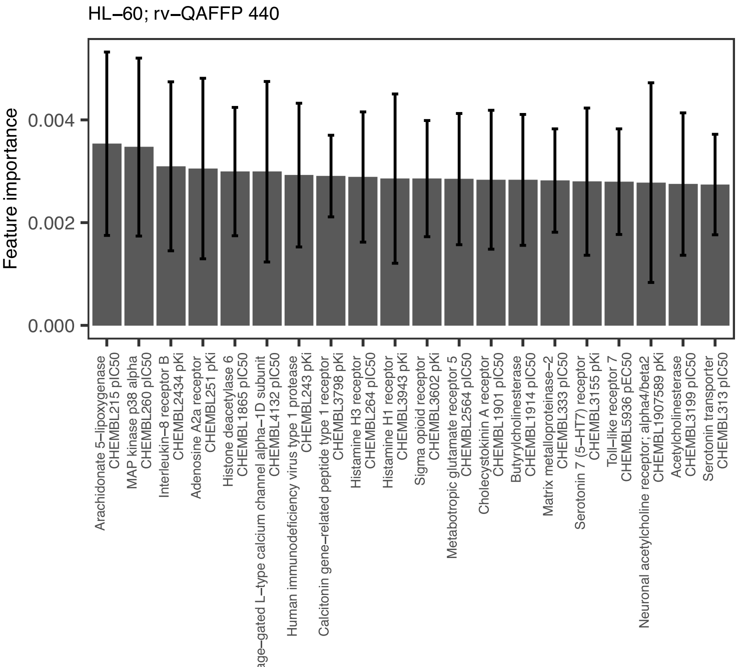
HL–60; rv–QAFFP 440
Feature importance
0.000
0.002
0.004
Arachidonate 5–lipoxygenase CHEMBL215 pIC50
MAP kinase p38 alpha CHEMBL260 pIC50
Interleukin–8 receptor B CHEMBL2434 pKi
Adenosine A2a receptor CHEMBL251 pKi
Histone deacetylase 6 CHEMBL1865 pIC50
Voltage–gated L–type calcium channel alpha–1D subunit CHEMBL4132 pIC50
Human immunodeficiency virus type 1 protease CHEMBL243 pKi
Calcitonin gene–related peptide type 1 receptor CHEMBL3798 pKi
Histamine H3 receptor CHEMBL264 pIC50
Histamine H1 receptor CHEMBL3943 pKi
Sigma opioid receptor CHEMBL3602 pKi
Metabotropic glutamate receptor 5 CHEMBL2564 pIC50
Cholecystokinin A receptor CHEMBL1901 pIC50
Butyrylcholinesterase CHEMBL1914 pIC50
Matrix metalloproteinase–2 CHEMBL333 pIC50
Serotonin 7 (5–HT7) receptor CHEMBL3155 pKi
Toll–like receptor 7 CHEMBL5936 pEC50
Neuronal acetylcholine receptor; alpha4/beta2 CHEMBL1907589 pKi
Acetylcholinesterase CHEMBL3199 pIC50
Serotonin transporter CHEMBL313 pIC50

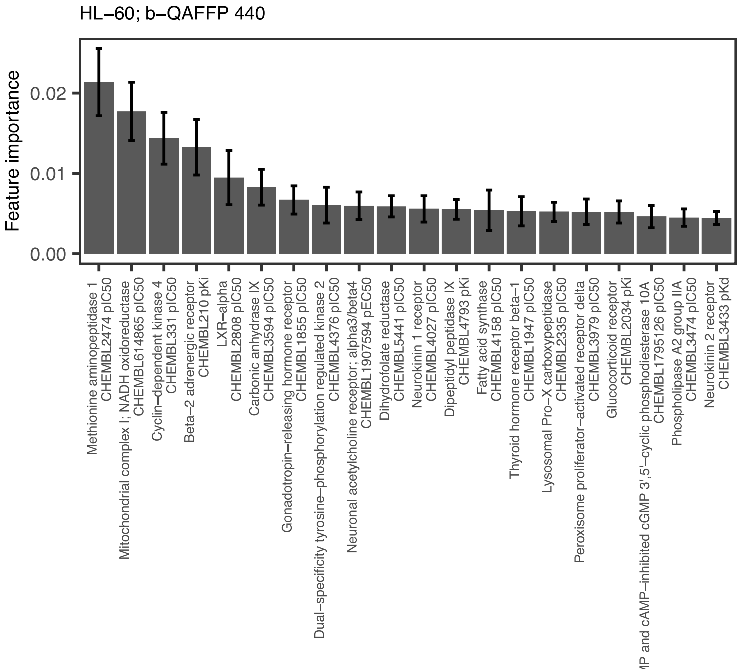
HL–60; b–QAFFP 440
Feature importance
0.00
0.01
0.02
Methionine aminopeptidase 1 CHEMBL2474 pIC50
Mitochondrial complex I; NADH oxidoreductase CHEMBL614865 pIC50
Cyclin–dependent kinase 4 CHEMBL331 pIC50
Beta–2 adrenergic receptor CHEMBL210 pKi
LXR–alpha CHEMBL2808 pIC50
Carbonic anhydrase IX CHEMBL3594 pIC50
Gonadotropin–releasing hormone receptor CHEMBL1855 pIC50
Dual–specificity tyrosine–phosphorylation regulated kinase 2 CHEMBL4376 pIC50
Neuronal acetylcholine receptor; alpha3/beta4 CHEMBL1907594 pEC50
Dihydrofolate reductase CHEMBL5441 pIC50
Neurokinin 1 receptor CHEMBL4027 pIC50
Dipeptidyl peptidase IX CHEMBL4793 pKi
Fatty acid synthase CHEMBL4158 pIC50
Thyroid hormone receptor beta–1 CHEMBL1947 pIC50
Lysosomal Pro–X carboxypeptidase CHEMBL2335 pIC50
Peroxisome proliferator–activated receptor delta CHEMBL3979 pIC50
Glucocorticoid receptor CHEMBL2034 pKi
cAMP and cAMP–inhibited cGMP 3',5'–cyclic phosphodiesterase 10A CHEMBL1795126 pIC50
Phospholipase A2 group IIA CHEMBL3474 pIC50
Neurokinin 2 receptor CHEMBL3433 pKd

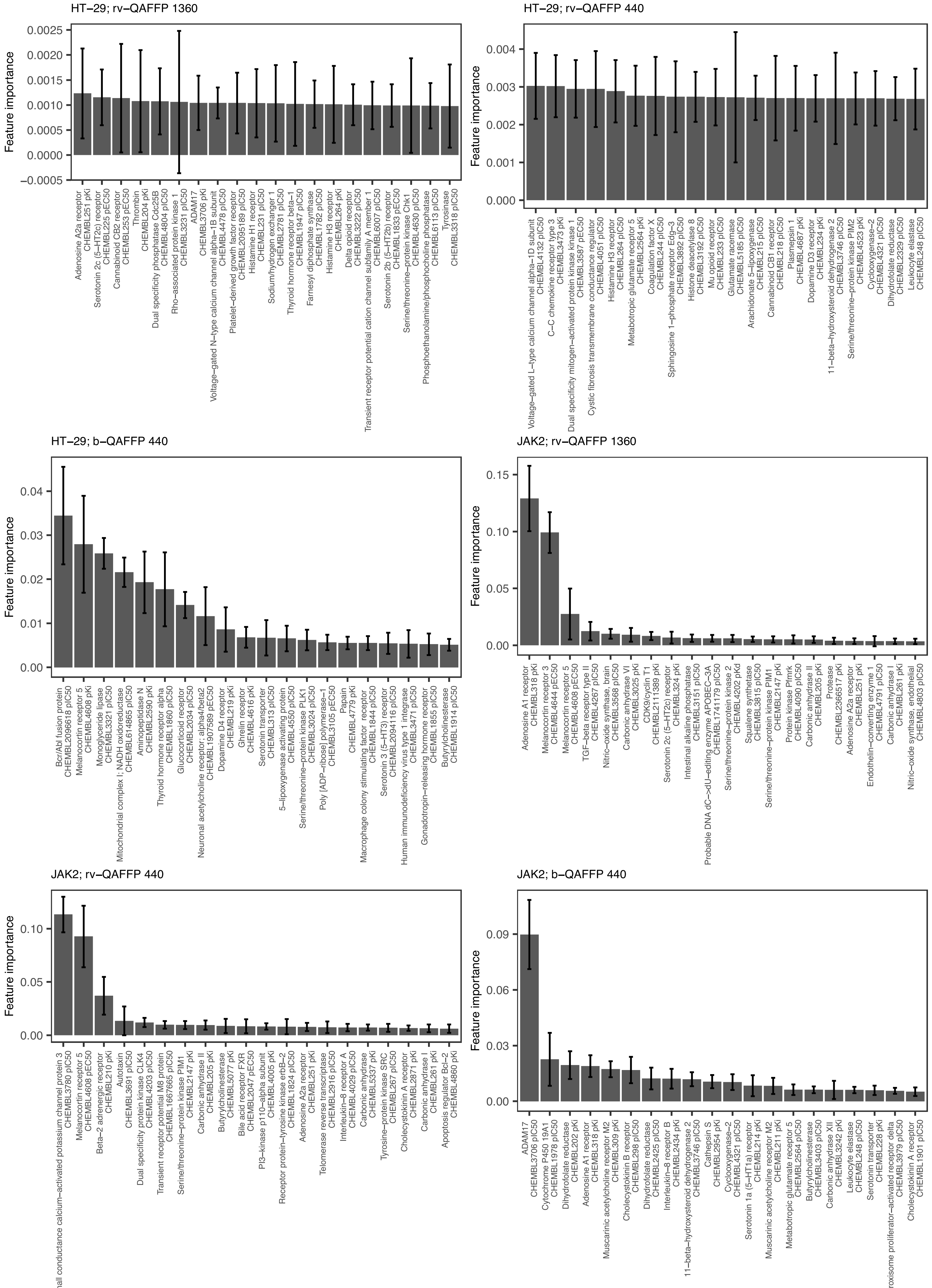

HT−29; rv−QAFFP 1360
Feature importance
0.0025
0.0020
0.0015
0.0010
0.0005
0.0000
−0.0005
Adenosine A2a receptor CHEMBL251 pKi
Serotonin 2c (5−HT2c) receptor CHEMBL225 pEC50
Cannabinoid CB2 receptor CHEMBL253 pEC50
Thrombin CHEMBL204 pKi
Dual specificity phosphatase Cdc25B CHEMBL4804 pIC50
Rho−associated protein kinase 1 CHEMBL3231 pIC50
ADAM17 CHEMBL3706 pKi
Voltage−gated N−type calcium channel alpha−1B subunit CHEMBL4478 pIC50
Platelet−derived growth factor receptor CHEMBL2095189 pIC50
Histamine H1 receptor CHEMBL231 pIC50
Sodium/hydrogen exchanger 1 CHEMBL2781 pIC50
Thyroid hormone receptor beta−1 CHEMBL1947 pIC50
Farnesyl diphosphate synthase CHEMBL1782 pIC50
Histamine H3 receptor CHEMBL264 pKi
Delta opioid receptor CHEMBL3222 pIC50
Transient receptor potential cation channel subfamily A member 1 CHEMBL6007 pIC50
Serotonin 2b (5−HT2b) receptor CHEMBL1833 pIC50
Serine/threonine−protein kinase Chk1 CHEMBL4630 pIC50
Phosphoethanolamine/phosphocholine phosphatase CHEMBL6113 pIC50
Tyrosinase CHEMBL3318 pIC50
HT−29; rv−QAFFP 440
Feature importance
0.004
0.003
0.002
0.001
0.000
Voltage−gated L−type calcium channel alpha−1D subunit CHEMBL4132 pIC50
C−C chemokine receptor type 3 CHEMBL3473 pKi
Dual specificity mitogen−activated protein kinase kinase 1 CHEMBL3587 pEC50
Cystic fibrosis transmembrane conductance regulator CHEMBL4051 pIC50
Histamine H3 receptor CHEMBL264 pIC50
Metabotropic glutamate receptor 5 CHEMBL2564 pKi
Coagulation factor X CHEMBL244 pIC50
Sphingosine 1−phosphate receptor Edg−3 CHEMBL3892 pIC50
Histone deacetylase 8 CHEMBL3192 pIC50
Mu opioid receptor CHEMBL233 pIC50
Glutamate racemase CHEMBL5185 pIC50
Arachidonate 5−lipoxygenase CHEMBL215 pIC50
Cannabinoid CB1 receptor CHEMBL218 pIC50
Plasmepsin 1 CHEMBL4687 pKi
Dopamine D3 receptor CHEMBL234 pKi
11−beta−hydroxysteroid dehydrogenase 2 CHEMBL3746 pIC50
Serine/threonine−protein kinase PIM2 CHEMBL4523 pKi
Cyclooxygenase−2 CHEMBL4321 pIC50
Dihydrofolate reductase CHEMBL2329 pIC50
Leukocyte elastase CHEMBL248 pIC50
HT−29; b−QAFFP 440
Feature importance
0.04
0.03
0.02
0.01
0.00
Bcr/Abl fusion protein CHEMBL2096618 pIC50
Melanocortin receptor 5 CHEMBL4608 pKi
Monoglyceride lipase CHEMBL3321 pIC50
Mitochondrial complex I; NADH oxidoreductase CHEMBL614865 pIC50
Aminopeptidase N CHEMBL2590 pKi
Thyroid hormone receptor alpha CHEMBL1860 pIC50
Glucocorticoid receptor CHEMBL2034 pIC50
Neuronal acetylcholine receptor; alpha4/beta2 CHEMBL1907589 pEC50
Dopamine D4 receptor CHEMBL219 pKi
Ghrelin receptor CHEMBL4616 pKi
Serotonin transporter CHEMBL313 pIC50
5−lipoxygenase activating protein CHEMBL4550 pIC50
Serine/threonine−protein kinase PLK1 CHEMBL3024 pIC50
Poly [ADP−ribose] polymerase−1 CHEMBL3105 pEC50
Papain CHEMBL4779 pKi
Macrophage colony stimulating factor receptor CHEMBL1844 pIC50
Serotonin 3 (5−HT3) receptor CHEMBL2094116 pIC50
Human immunodeficiency virus type 1 integrase CHEMBL3471 pIC50
Gonadotropin−releasing hormone receptor CHEMBL1855 pIC50
Butyrylcholinesterase CHEMBL1914 pIC50
JAK2; rv−QAFFP 1360
Feature importance
0.15
0.10
0.05
0.00
Adenosine A1 receptor CHEMBL318 pKi
Melanocortin receptor 3 CHEMBL4644 pEC50
Melanocortin receptor 5 CHEMBL4608 pEC50
TGF−beta receptor type II CHEMBL4267 pIC50
Nitric−oxide synthase, brain CHEMBL3568 pIC50
Carbonic anhydrase VI CHEMBL3025 pKi
CDK9/cyclin T1 CHEMBL2111389 pKi
Serotonin 2c (5−HT2c) receptor CHEMBL324 pKi
Intestinal alkaline phosphatase CHEMBL3151 pIC50
Probable DNA dC−>dU−editing enzyme APOBEC−3A CHEMBL1741179 pIC50
Serine/threonine−protein kinase 2 CHEMBL4202 pKd
Squalene synthetase CHEMBL3815 pIC50
Serine/threonine−protein kinase PIM1 CHEMBL2147 pKi
Protein kinase Pfmrk CHEMBL4090 pIC50
Carbonic anhydrase II CHEMBL205 pKi
Protease CHEMBL2366517 pKi
Adenosine A2a receptor CHEMBL251 pKi
Endothelin−converting enzyme 1 CHEMBL4791 pIC50
Carbonic anhydrase I CHEMBL261 pKi
Nitric−oxide synthase, endothelial CHEMBL4803 pIC50
JAK2; rv−QAFFP 440
Feature importance
0.10
0.05
0.00
Small conductance calcium−activated potassium channel protein 3 CHEMBL3780 pIC50
Melanocortin receptor 5 CHEMBL4608 pEC50
Beta−2 adrenergic receptor CHEMBL210 pKi
Autotaxin CHEMBL3691 pIC50
Dual specificity protein kinase CLK4 CHEMBL4203 pIC50
Transient receptor potential M8 protein CHEMBL1667665 pIC50
Serine/threonine−protein kinase PIM1 CHEMBL2147 pKi
Carbonic anhydrase II CHEMBL205 pKi
Butyrylcholinesterase CHEMBL5077 pKi
Bile acid receptor FXR CHEMBL2047 pEC50
PI3−kinase p110−alpha subunit CHEMBL4005 pKi
Receptor protein−tyrosine kinase erbB−2 CHEMBL1824 pIC50
Adenosine A2a receptor CHEMBL251 pKi
Telomerase reverse transcriptase CHEMBL2916 pIC50
Interleukin−8 receptor A CHEMBL4029 pIC50
Carbonic anhydrase CHEMBL5337 pKi
Tyrosine−protein kinase SRC CHEMBL267 pIC50
Cholecystokinin A receptor CHEMBL2871 pKi
Carbonic anhydrase I CHEMBL261 pKi
Apoptosis regulator Bcl−2 CHEMBL4860 pKi
JAK2; b−QAFFP 440
Feature importance
0.09
0.06
0.03
0.00
ADAM17 CHEMBL3706 pIC50
Cytochrome P450 19A1 CHEMBL1978 pIC50
Dihydrofolate reductase CHEMBL202 pKi
Adenosine A1 receptor CHEMBL318 pKi
Muscarinic acetylcholine receptor M2 CHEMBL309 pKi
Cholecystokinin B receptor CHEMBL298 pIC50
Dihydrofolate reductase CHEMBL2425 pIC50
Interleukin−8 receptor B CHEMBL2434 pKi
11−beta−hydroxysteroid dehydrogenase 2 CHEMBL3746 pIC50
Cathepsin S CHEMBL2954 pIC50
Cyclooxygenase−2 CHEMBL4321 pIC50
Serotonin 1a (5−HT1a) receptor CHEMBL214 pIC50
Muscarinic acetylcholine receptor M2 CHEMBL211 pKi
Metabotropic glutamate receptor 5 CHEMBL2564 pIC50
Butyrylcholinesterase CHEMBL3403 pIC50
Carbonic anhydrase XII CHEMBL3242 pKi
Leukocyte elastase CHEMBL248 pIC50
Serotonin transporter CHEMBL228 pKi
Peroxisome proliferator−activated receptor delta CHEMBL3979 pIC50
Cholecystokinin A receptor CHEMBL1901 pIC50

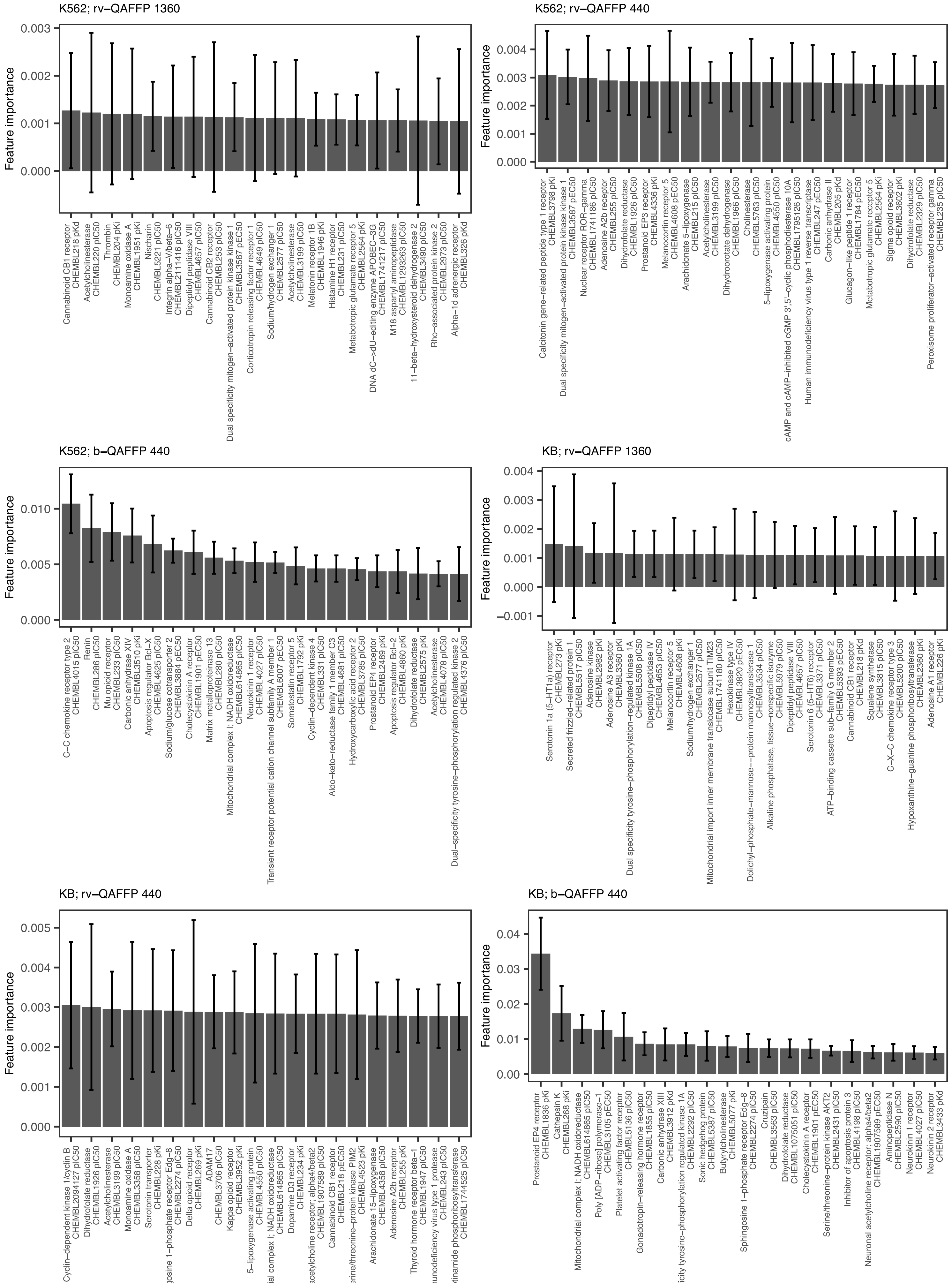

K562; rv−QAFFP 1360
Feature importance
Cannabinoid CB1 receptor CHEMBL218 pKd
Acetylcholinesterase CHEMBL220 pIC50
Thrombin CHEMBL204 pKi
Monoamine oxidase A CHEMBL1951 pKi
Nischarin CHEMBL5221 pIC50
Integrin alpha−V/beta−6 CHEMBL2111416 pIC50
Dipeptidyl peptidase VIII CHEMBL4657 pIC50
Cannabinoid CB2 receptor CHEMBL253 pIC50
Dual specificity mitogen−activated protein kinase kinase 1 CHEMBL3587 pEC50
Corticotropin releasing factor receptor 1 CHEMBL4649 pIC50
Sodium/hydrogen exchanger 1 CHEMBL2577 pIC50
Acetylcholinesterase CHEMBL3199 pIC50
Melatonin receptor 1B CHEMBL1946 pKi
Histamine H1 receptor CHEMBL231 pIC50
Metabotropic glutamate receptor 5 CHEMBL2564 pKi
DNA dC−>dU−editing enzyme APOBEC−3G CHEMBL1741217 pIC50
M18 aspartyl aminopeptidase CHEMBL1293263 pIC50
11−beta−hydroxysteroid dehydrogenase 2 CHEMBL3490 pIC50
Rho−associated protein kinase 2 CHEMBL2973 pIC50
Alpha−1d adrenergic receptor CHEMBL326 pKd
K562; rv−QAFFP 440
Feature importance
Calcitonin gene−related peptide type 1 receptor CHEMBL3798 pKi
Dual specificity mitogen−activated protein kinase kinase 1 CHEMBL3587 pEC50
Nuclear receptor ROR−gamma CHEMBL1741186 pIC50
Adenosine A2b receptor CHEMBL255 pIC50
Dihydrofolate reductase CHEMBL1926 pIC50
Prostanoid EP3 receptor CHEMBL4336 pKi
Melanocortin receptor 5 CHEMBL4608 pEC50
Arachidonate 5−lipoxygenase CHEMBL215 pIC50
Acetylcholinesterase CHEMBL3199 pIC50
Dihydroorotate dehydrogenase CHEMBL1966 pIC50
Cholinesterase CHEMBL5763 pIC50
5−lipoxygenase activating protein CHEMBL4550 pIC50
cAMP and cAMP−inhibited cGMP 3',5'−cyclic phosphodiesterase 10A CHEMBL1795126 pIC50
Human immunodeficiency virus type 1 reverse transcriptase CHEMBL247 pEC50
Carbonic anhydrase II CHEMBL205 pKd
Glucagon−like peptide 1 receptor CHEMBL1784 pEC50
Metabotropic glutamate receptor 5 CHEMBL2564 pKi
Sigma opioid receptor CHEMBL3602 pKi
Dihydrofolate reductase CHEMBL2329 pIC50
Peroxisome proliferator−activated receptor gamma CHEMBL235 pIC50
K562; b−QAFFP 440
Feature importance
C−C chemokine receptor type 2 CHEMBL4015 pIC50
Renin CHEMBL286 pIC50
Mu opioid receptor CHEMBL233 pIC50
Carbonic anhydrase XIV CHEMBL3510 pKi
Apoptosis regulator Bcl−X CHEMBL4625 pIC50
Sodium/glucose cotransporter 2 CHEMBL3884 pEC50
Cholecystokinin A receptor CHEMBL1901 pEC50
Matrix metalloproteinase 13 CHEMBL280 pIC50
Mitochondrial complex I; NADH oxidoreductase CHEMBL614865 pIC50
Neurokinin 1 receptor CHEMBL4027 pIC50
Transient receptor potential cation channel subfamily A member 1 CHEMBL6007 pIC50
Somatostatin receptor 5 CHEMBL1792 pIC50
Cyclin−dependent kinase 4 CHEMBL331 pIC50
Aldo−keto−reductase family 1 member C3 CHEMBL4681 pIC50
Hydroxycarboxylic acid receptor 2 CHEMBL3785 pIC50
Prostanoid EP4 receptor CHEMBL2489 pKi
Apoptosis regulator Bcl−2 CHEMBL4860 pKi
Dihydrofolate reductase CHEMBL2575 pKi
Acetylcholinesterase CHEMBL4078 pIC50
Dual−specificity tyrosine−phosphorylation regulated kinase 2 CHEMBL4376 pIC50
KB; rv−QAFFP 1360
Feature importance
Serotonin 1a (5−HT1a) receptor CHEMBL273 pKi
Secreted frizzled−related protein 1 CHEMBL5517 pIC50
Adenosine kinase CHEMBL2982 pKi
Adenosine A3 receptor CHEMBL3360 pKi
Dual specificity tyrosine−phosphorylation−regulated kinase 1A CHEMBL5508 pIC50
Dipeptidyl peptidase IV CHEMBL4653 pIC50
Melanocortin receptor 5 CHEMBL4608 pIC50
Sodium/hydrogen exchanger 1 CHEMBL2577 pIC50
Mitochondrial import inner membrane translocase subunit TIM23 CHEMBL1741180 pIC50
Hexokinase type IV CHEMBL3820 pEC50
Dolichyl−phosphate−mannose−−protein mannosyltransferase 1 CHEMBL3534 pIC50
Alkaline phosphatase, tissue−nonspecific isozyme CHEMBL5979 pIC50
Dipeptidyl peptidase VIII CHEMBL4657 pIC50
Serotonin 6 (5−HT6) receptor CHEMBL3371 pIC50
ATP−binding cassette sub−family G member 2 CHEMBL5393 pEC50
Cannabinoid CB1 receptor CHEMBL218 pKd
Squalene synthetase CHEMBL3815 pIC50
C−X−C chemokine receptor type 3 CHEMBL5200 pIC50
Hypoxanthine−guanine phosphoribosyltransferase CHEMBL2360 pKi
Adenosine A1 receptor CHEMBL226 pKi
KB; rv−QAFFP 440
Feature importance
Cyclin−dependent kinase 1/cyclin B CHEMBL2094127 pIC50
Dihydrofolate reductase CHEMBL1926 pIC50
Acetylcholinesterase CHEMBL3199 pIC50
Monoamine oxidase A CHEMBL3358 pIC50
Serotonin transporter CHEMBL228 pKi
Sphingosine 1−phosphate receptor Edg−8 CHEMBL2274 pIC50
Delta opioid receptor CHEMBL269 pIC50
ADAM17 CHEMBL3706 pIC50
Kappa opioid receptor CHEMBL3952 pKi
5−lipoxygenase activating protein CHEMBL4550 pIC50
Mitochondrial complex I; NADH oxidoreductase CHEMBL614865 pIC50
Dopamine D3 receptor CHEMBL234 pKi
Neuronal acetylcholine receptor; alpha4/beta2 CHEMBL1907589 pIC50
Cannabinoid CB1 receptor CHEMBL218 pIC50
Serine/threonine−protein kinase PIM2 CHEMBL4523 pKi
Arachidonate 15−lipoxygenase CHEMBL4358 pIC50
Adenosine A2b receptor CHEMBL255 pKi
Thyroid hormone receptor beta−1 CHEMBL1947 pIC50
Human immunodeficiency virus type 1 protease CHEMBL243 pIC50
Nicotinamide phosphoribosyltransferase CHEMBL1744525 pIC50
KB; b−QAFFP 440
Feature importance
Prostanoid EP4 receptor CHEMBL1836 pKi
Cathepsin K CHEMBL268 pKi
Mitochondrial complex I; NADH oxidoreductase CHEMBL614865 pIC50
Poly [ADP−ribose] polymerase−1 CHEMBL3105 pEC50
Platelet activating factor receptor CHEMBL5136 pIC50
Gonadotropin−releasing hormone receptor CHEMBL1855 pIC50
Carbonic anhydrase XIII CHEMBL3912 pKd
Dual−specificity tyrosine−phosphorylation regulated kinase 1A CHEMBL2292 pIC50
Sonic hedgehog protein CHEMBL5387 pIC50
Butyrylcholinesterase CHEMBL5077 pKi
Sphingosine 1−phosphate receptor Edg−8 CHEMBL2274 pIC50
Cruzipain CHEMBL3563 pIC50
Dihydrofolate reductase CHEMBL1075051 pIC50
Cholecystokinin A receptor CHEMBL1901 pEC50
Serine/threonine−protein kinase AKT2 CHEMBL2431 pIC50
Inhibitor of apoptosis protein 3 CHEMBL4198 pIC50
Neuronal acetylcholine receptor; alpha4/beta2 CHEMBL1907589 pIC50
Aminopeptidase N CHEMBL2590 pIC50
Neurokinin 1 receptor CHEMBL4027 pIC50
Neurokinin 2 receptor CHEMBL3433 pKd

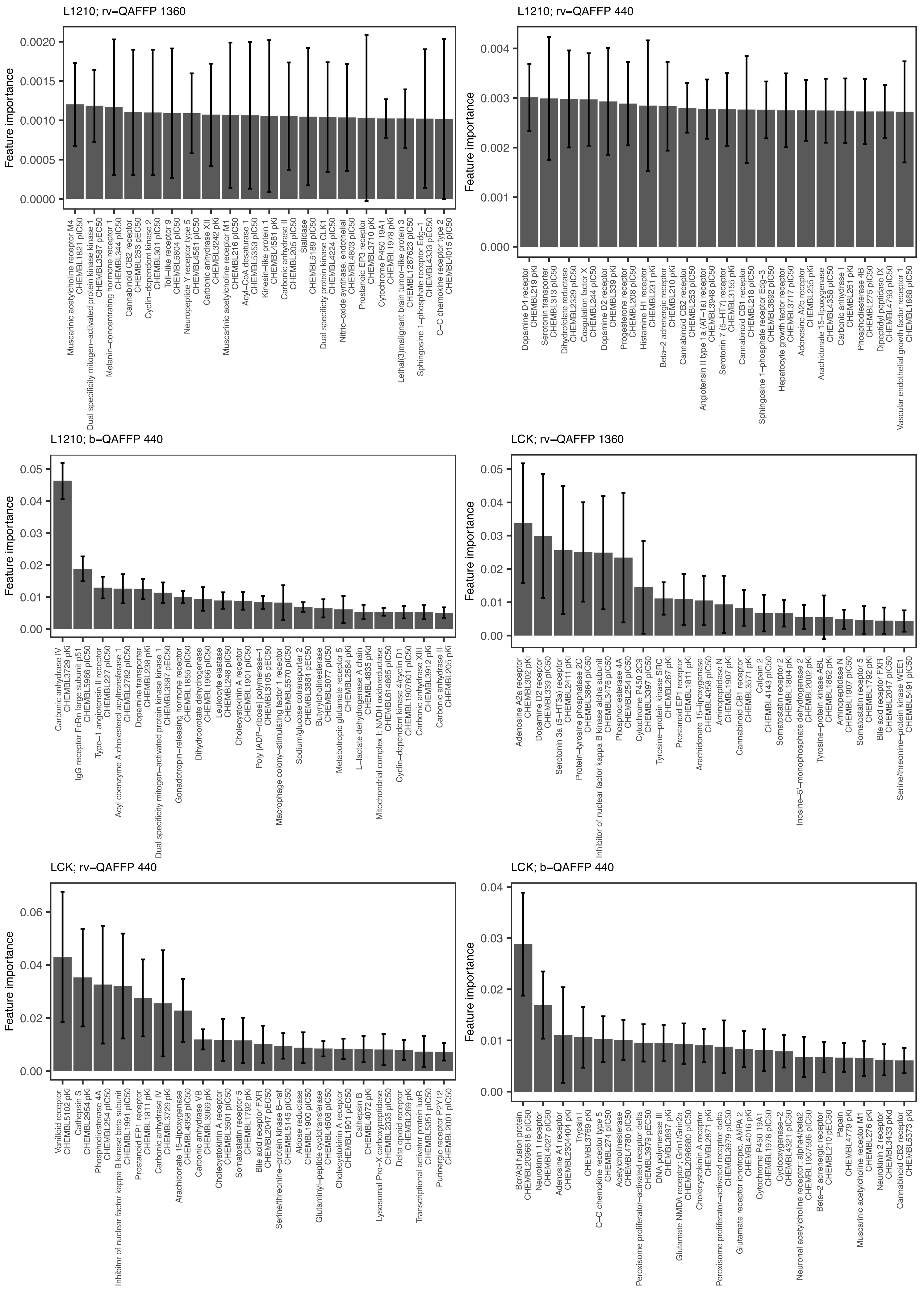
L1210; rv−QAFFP 1360
Feature importance
0.0000
0.0005
0.0010
0.0015
0.0020
Muscarinic acetylcholine receptor M4 CHEMBL1821 pIC50
Dual specificity mitogen−activated protein kinase kinase 1 CHEMBL3587 pEC50
Melanin−concentrating hormone receptor 1 CHEMBL344 pIC50
Cannabinoid CB2 receptor CHEMBL253 pEC50
Cyclin−dependent kinase 2 CHEMBL301 pIC50
Toll−like receptor 9 CHEMBL5804 pIC50
Neuropeptide Y receptor type 5 CHEMBL4561 pIC50
Carbonic anhydrase XII CHEMBL3242 pKi
Muscarinic acetylcholine receptor M1 CHEMBL216 pIC50
Acyl−CoA desaturase 1 CHEMBL5353 pIC50
Kinesin−like protein 1 CHEMBL4581 pKi
Carbonic anhydrase II CHEMBL205 pIC50
Sialidase CHEMBL5189 pIC50
Dual specificity protein kinase CLK1 CHEMBL4224 pIC50
Nitric−oxide synthase, endothelial CHEMBL4803 pIC50
Prostanoid EP3 receptor CHEMBL3710 pKi
Cytochrome P450 19A1 CHEMBL1978 pKi
Lethal(3)malignant brain tumor−like protein 3 CHEMBL1287623 pIC50
Sphingosine 1−phosphate receptor Edg−1 CHEMBL4333 pEC50
C−C chemokine receptor type 2 CHEMBL4015 pIC50
L1210; rv−QAFFP 440
Feature importance
0.000
0.001
0.002
0.003
0.004
Dopamine D4 receptor CHEMBL219 pKi
Serotonin transporter CHEMBL313 pIC50
Dihydrofolate reductase CHEMBL2329 pIC50
Coagulation factor X CHEMBL244 pIC50
Dopamine D2 receptor CHEMBL339 pKi
Progesterone receptor CHEMBL208 pIC50
Histamine H1 receptor CHEMBL231 pKi
Beta−2 adrenergic receptor CHEMBL210 pKi
Cannabinoid CB2 receptor CHEMBL253 pIC50
Angiotensin II type 1a (AT−1a) receptor CHEMBL3948 pIC50
Serotonin 7 (5−HT7) receptor CHEMBL3155 pKi
Cannabinoid CB1 receptor CHEMBL218 pIC50
Sphingosine 1−phosphate receptor Edg−3 CHEMBL3892 pIC50
Hepatocyte growth factor receptor CHEMBL3717 pIC50
Adenosine A2b receptor CHEMBL255 pKi
Arachidonate 15−lipoxygenase CHEMBL4358 pIC50
Carbonic anhydrase I CHEMBL261 pKi
Phosphodiesterase 4B CHEMBL275 pIC50
Dipeptidyl peptidase IX CHEMBL4793 pIC50
Vascular endothelial growth factor receptor 1 CHEMBL1868 pIC50
L1210; b−QAFFP 440
Feature importance
0.00
0.01
0.02
0.03
0.04
0.05
Carbonic anhydrase IV CHEMBL3729 pKi
IgG receptor FcRn large subunit p51 CHEMBL5966 pIC50
Type−1 angiotensin II receptor CHEMBL227 pIC50
Acyl coenzyme A:cholesterol acyltransferase 1 CHEMBL2782 pIC50
Dopamine transporter CHEMBL238 pKi
Dual specificity mitogen−activated protein kinase kinase 1 CHEMBL3587 pEC50
Gonadotropin−releasing hormone receptor CHEMBL1855 pIC50
Dihydroorotate dehydrogenase CHEMBL1966 pIC50
Leukocyte elastase CHEMBL248 pIC50
Cholecystokinin A receptor CHEMBL1901 pIC50
Poly [ADP−ribose] polymerase−1 CHEMBL3105 pEC50
Macrophage colony−stimulating factor 1 receptor CHEMBL5570 pIC50
Sodium/glucose cotransporter 2 CHEMBL3884 pEC50
Butyrylcholinesterase CHEMBL5077 pIC50
Metabotropic glutamate receptor 5 CHEMBL2564 pKi
L−lactate dehydrogenase A chain CHEMBL4835 pKd
Mitochondrial complex I; NADH oxidoreductase CHEMBL614865 pIC50
Cyclin−dependent kinase 4/cyclin D1 CHEMBL1907601 pIC50
Carbonic anhydrase XIII CHEMBL3912 pKi
Carbonic anhydrase II CHEMBL205 pKi
LCK; rv−QAFFP 1360
Feature importance
0.00
0.01
0.02
0.03
0.04
0.05
Adenosine A2a receptor CHEMBL302 pKi
Dopamine D2 receptor CHEMBL339 pIC50
Serotonin 3a (5−HT3a) receptor CHEMBL2411 pKi
Protein−tyrosine phosphatase 2C CHEMBL3864 pIC50
Inhibitor of nuclear factor kappa B kinase alpha subunit CHEMBL3476 pIC50
Phosphodiesterase 4A CHEMBL254 pIC50
Cytochrome P450 2C9 CHEMBL3397 pIC50
Tyrosine−protein kinase SRC CHEMBL267 pKi
Prostanoid EP1 receptor CHEMBL1811 pKi
Arachidonate 15−lipoxygenase CHEMBL4358 pIC50
Aminopeptidase N CHEMBL1907 pKi
Cannabinoid CB1 receptor CHEMBL3571 pKi
Calpain 2 CHEMBL4143 pIC50
Somatostatin receptor 2 CHEMBL1804 pKi
Inosine−5'−monophosphate dehydrogenase 2 CHEMBL2002 pKi
Tyrosine−protein kinase ABL CHEMBL1862 pKi
Aminopeptidase N CHEMBL1907 pIC50
Somatostatin receptor 5 CHEMBL1792 pKi
Bile acid receptor FXR CHEMBL2047 pIC50
Serine/threonine−protein kinase WEE1 CHEMBL5491 pIC50
LCK; rv−QAFFP 440
Feature importance
0.00
0.02
0.04
0.06
Vanilloid receptor CHEMBL5102 pKi
Cathepsin S CHEMBL2954 pKi
Phosphodiesterase 4A CHEMBL254 pIC50
Inhibitor of nuclear factor kappa B kinase beta subunit CHEMBL1991 pIC50
Prostanoid EP1 receptor CHEMBL1811 pKi
Carbonic anhydrase IV CHEMBL3729 pKi
Arachidonate 15−lipoxygenase CHEMBL4358 pIC50
Carbonic anhydrase VB CHEMBL3969 pKi
Cholecystokinin A receptor CHEMBL3501 pIC50
Somatostatin receptor 5 CHEMBL1792 pKi
Bile acid receptor FXR CHEMBL2047 pEC50
Serine/threonine−protein kinase B−raf CHEMBL5145 pIC50
Aldose reductase CHEMBL1900 pIC50
Glutaminyl−peptide cyclotransferase CHEMBL4508 pIC50
Cholecystokinin A receptor CHEMBL1901 pEC50
Cathepsin B CHEMBL4072 pKi
Lysosomal Pro−X carboxypeptidase CHEMBL2335 pIC50
Delta opioid receptor CHEMBL269 pKi
Transcriptional activator protein luxR CHEMBL5351 pIC50
Purinergic receptor P2Y12 CHEMBL2001 pIC50
LCK; b−QAFFP 440
Feature importance
0.00
0.01
0.02
0.03
0.04
Bcr/Abl fusion protein CHEMBL2096618 pIC50
Neurokinin 1 receptor CHEMBL4027 pIC50
Adenosine A1 receptor CHEMBL2304404 pKi
Trypsin I CHEMBL3769 pKi
C−C chemokine receptor type 5 CHEMBL274 pIC50
Acetylcholinesterase CHEMBL4780 pIC50
Peroxisome proliferator−activated receptor delta CHEMBL3979 pEC50
DNA polymerase III CHEMBL3897 pKi
Glutamate NMDA receptor; Grin1/Grin2a CHEMBL2096680 pIC50
Cholecystokinin A receptor CHEMBL2871 pKi
Peroxisome proliferator−activated receptor delta CHEMBL3979 pIC50
Glutamate receptor ionotropic, AMPA 2 CHEMBL4016 pKi
Cytochrome P450 19A1 CHEMBL1978 pIC50
Cyclooxygenase−2 CHEMBL4321 pIC50
Neuronal acetylcholine receptor; alpha4/beta2 CHEMBL1907596 pIC50
Beta−2 adrenergic receptor CHEMBL210 pEC50
Papain CHEMBL4779 pKi
Muscarinic acetylcholine receptor M1 CHEMBL276 pKi
Neurokinin 2 receptor CHEMBL3433 pKd
Cannabinoid CB2 receptor CHEMBL5373 pKi

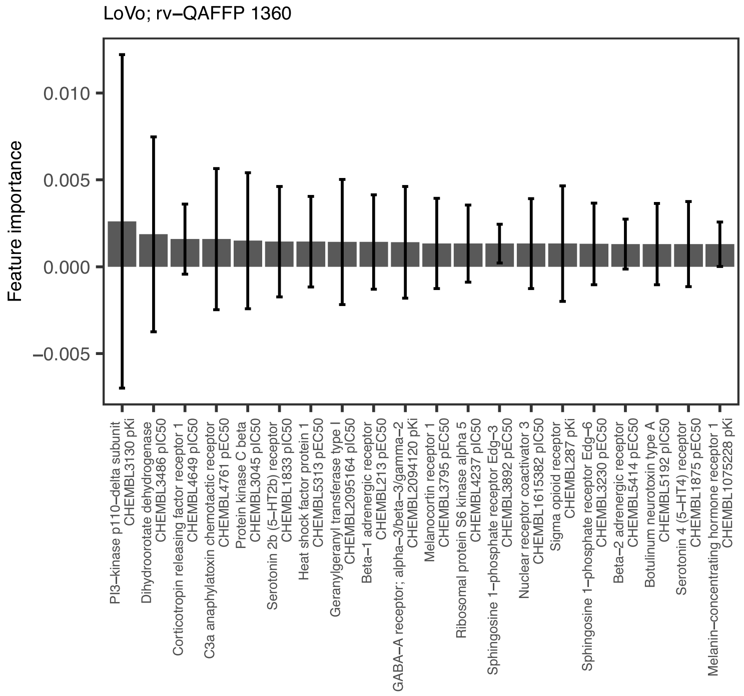
LoVo; rv–QAFFP 1360
Feature importance
0.010
0.005
0.000
–0.005
PI3–kinase p110–delta subunit CHEMBL3130 pKi
Dihydroorotate dehydrogenase CHEMBL3486 pIC50
Corticotropin releasing factor receptor 1 CHEMBL4649 pIC50
C3a anaphylatoxin chemotactic receptor CHEMBL4761 pEC50
Protein kinase C beta CHEMBL3045 pIC50
Serotonin 2b (5–HT2b) receptor CHEMBL1833 pIC50
Heat shock factor protein 1 CHEMBL5313 pEC50
Geranylgeranyl transferase type I CHEMBL2095164 pIC50
Beta–1 adrenergic receptor CHEMBL213 pEC50
GABA–A receptor; alpha–3/beta–3/gamma–2 CHEMBL2094120 pEC50
Melanocortin receptor 1 CHEMBL3795 pEC50
Ribosomal protein S6 kinase alpha 5 CHEMBL4237 pIC50
Sphingosine 1–phosphate receptor Edg–3 CHEMBL3892 pEC50
Nuclear receptor coactivator 3 CHEMBL1615382 pIC50
Sigma opioid receptor CHEMBL287 pKi
Sphingosine 1–phosphate receptor Edg–6 CHEMBL3230 pEC50
Beta–2 adrenergic receptor CHEMBL5414 pEC50
Botulinum neurotoxin type A CHEMBL5192 pIC50
Serotonin 4 (5–HT4) receptor CHEMBL1875 pEC50
Melanin–concentrating hormone receptor 1 CHEMBL1075228 pKi

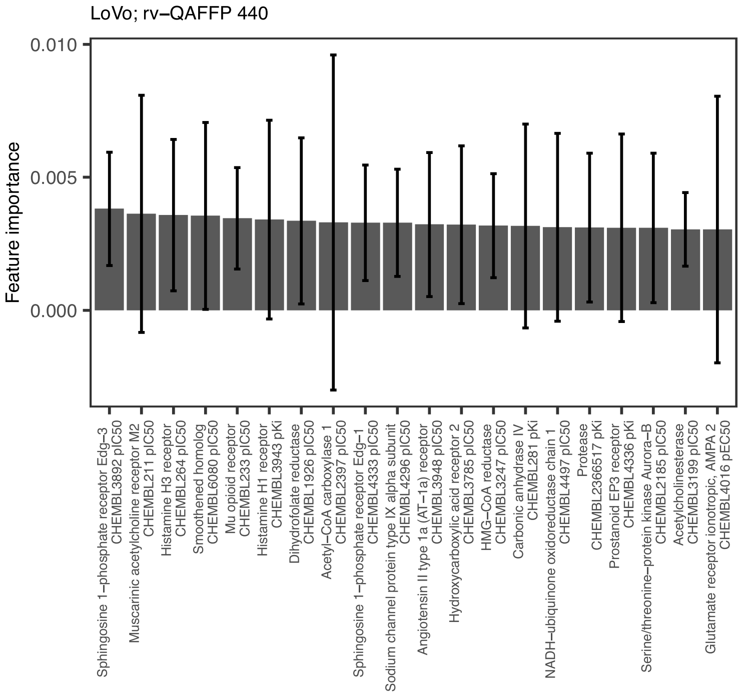
LoVo; rv–QAFFP 440
Feature importance
0.010
0.005
0.000
Sphingosine 1–phosphate receptor Edg–3 CHEMBL3892 pIC50
Muscarinic acetylcholine receptor M2 CHEMBL211 pIC50
Histamine H3 receptor CHEMBL264 pIC50
Smoothened homolog CHEMBL6080 pIC50
Mu opioid receptor CHEMBL233 pIC50
Histamine H1 receptor CHEMBL3943 pKi
Dihydrofolate reductase CHEMBL1926 pIC50
Acetyl–CoA carboxylase 1 CHEMBL2397 pIC50
Sphingosine 1–phosphate receptor Edg–1 CHEMBL4333 pIC50
Sodium channel protein type IX alpha subunit CHEMBL4296 pIC50
Angiotensin II type 1a (AT–1a) receptor CHEMBL3948 pIC50
Hydroxycarboxylic acid receptor 2 CHEMBL3785 pIC50
HMG–CoA reductase CHEMBL3247 pIC50
Carbonic anhydrase IV CHEMBL281 pKi
NADH–ubiquinone oxidoreductase chain 1 CHEMBL4497 pIC50
Protease CHEMBL2366517 pKi
Prostanoid EP3 receptor CHEMBL4336 pKi
Serine/threonine–protein kinase Aurora–B CHEMBL2185 pIC50
Acetylcholinesterase CHEMBL3199 pIC50
Glutamate receptor ionotropic, AMPA 2 CHEMBL4016 pEC50

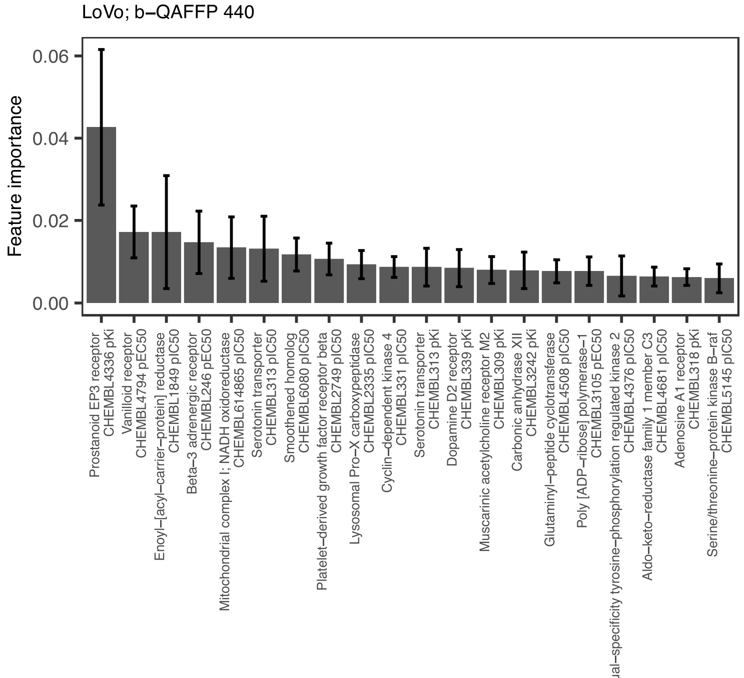
LoVo; b–QAFFP 440
Feature importance
0.06
0.04
0.02
0.00
Prostanoid EP3 receptor CHEMBL4336 pKi
Vanilloid receptor CHEMBL4794 pEC50
Enoyl–[acyl–carrier–protein] reductase CHEMBL1849 pIC50
Beta–3 adrenergic receptor CHEMBL246 pEC50
Mitochondrial complex I; NADH oxidoreductase CHEMBL614865 pIC50
Serotonin transporter CHEMBL313 pIC50
Smoothened homolog CHEMBL6080 pIC50
Platelet–derived growth factor receptor beta CHEMBL2749 pIC50
Lysosomal Pro–X carboxypeptidase CHEMBL2335 pIC50
Cyclin–dependent kinase 4 CHEMBL331 pIC50
Serotonin transporter CHEMBL313 pKi
Dopamine D2 receptor CHEMBL339 pKi
Muscarinic acetylcholine receptor M2 CHEMBL309 pKi
Carbonic anhydrase XII CHEMBL3242 pKi
Glutaminyl–peptide cyclotransferase CHEMBL4508 pIC50
Poly [ADP–ribose] polymerase–1 CHEMBL3105 pEC50
Dual–specificity tyrosine–phosphorylation regulated kinase 2 CHEMBL4376 pIC50
Aldo–keto–reductase family 1 member C3 CHEMBL4681 pIC50
Adenosine A1 receptor CHEMBL318 pKi
Serine/threonine–protein kinase B–raf CHEMBL5145 pIC50

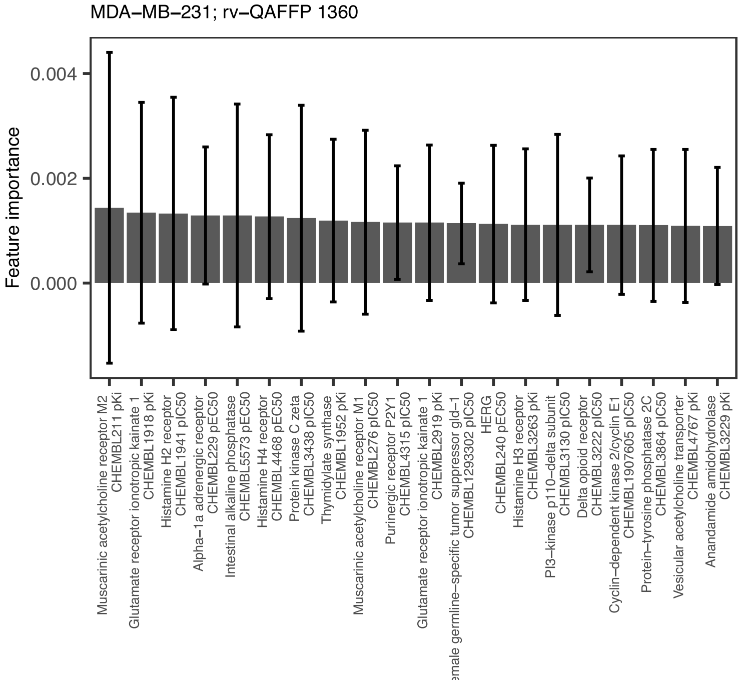
MDA–MB–231; rv–QAFFP 1360
Feature importance
0.004
0.002
0.000
Muscarinic acetylcholine receptor M2 CHEMBL211 pKi
Glutamate receptor ionotropic kainate 1 CHEMBL1918 pIC50
Histamine H2 receptor CHEMBL1941 pIC50
Alpha–1a adrenergic receptor CHEMBL229 pEC50
Intestinal alkaline phosphatase CHEMBL5573 pEC50
Histamine H4 receptor CHEMBL4468 pEC50
Protein kinase C zeta CHEMBL3438 pIC50
Thymidylate synthase CHEMBL1952 pIC50
Muscarinic acetylcholine receptor M1 CHEMBL276 pIC50
Purinergic receptor P2Y1 CHEMBL4315 pIC50
Glutamate receptor ionotropic kainate 1 CHEMBL2919 pIC50
Female germline–specific tumor suppressor gld–1 CHEMBL1293302 pIC50
HERG CHEMBL240 pEC50
Histamine H3 receptor CHEMBL3263 pKi
PI3–kinase p110–delta subunit CHEMBL3130 pIC50
Delta opioid receptor CHEMBL3222 pIC50
Cyclin–dependent kinase 2/cyclin E1 CHEMBL1907605 pIC50
Protein–tyrosine phosphatase 2C CHEMBL3864 pIC50
Vesicular acetylcholine transporter CHEMBL4767 pKi
Anandamide amidohydrolase CHEMBL3229 pKi

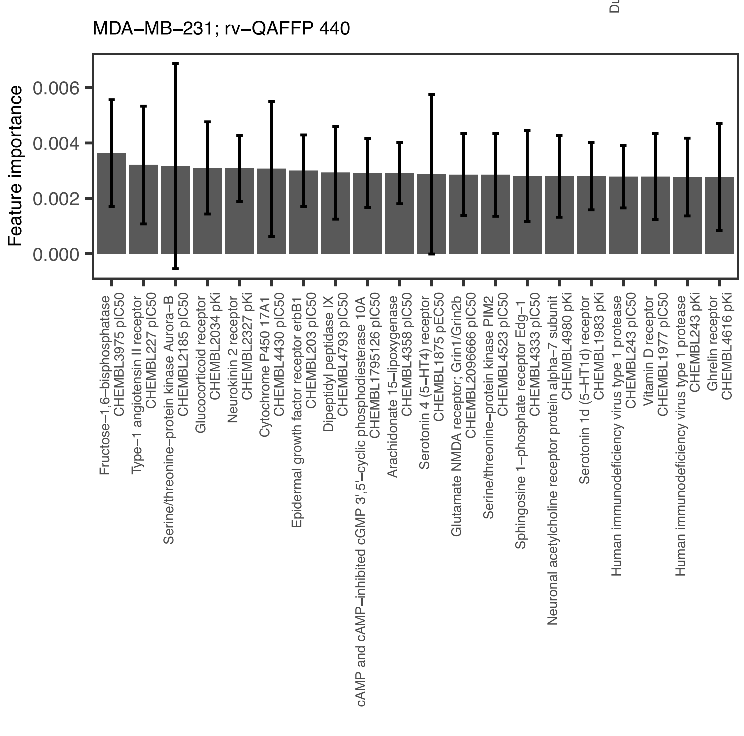
MDA–MB–231; rv–QAFFP 440
Feature importance
0.006
0.004
0.002
0.000
Fructose–1,6–bisphosphatase CHEMBL3975 pIC50
Type–1 angiotensin II receptor CHEMBL227 pIC50
Serine/threonine–protein kinase Aurora–B CHEMBL2185 pIC50
Glucocorticoid receptor CHEMBL2034 pKi
Neurokinin 2 receptor CHEMBL2327 pKi
Cytochrome P450 17A1 CHEMBL4430 pIC50
Epidermal growth factor receptor erbB1 CHEMBL203 pIC50
Dipeptidyl peptidase IX CHEMBL4793 pIC50
cAMP and cAMP–inhibited cGMP 3',5'–cyclic phosphodiesterase 10A CHEMBL1795126 pIC50
Arachidonate 15–lipoxygenase CHEMBL4358 pIC50
Serotonin 4 (5–HT4) receptor CHEMBL1875 pEC50
Glutamate NMDA receptor; Grin1/Grin2b CHEMBL2096666 pIC50
Serine/threonine–protein kinase PIM2 CHEMBL4523 pIC50
Sphingosine 1–phosphate receptor Edg–1 CHEMBL4333 pEC50
Neuronal acetylcholine receptor protein alpha–7 subunit CHEMBL4980 pKi
Serotonin 1d (5–HT1d) receptor CHEMBL1983 pIC50
Human immunodeficiency virus type 1 protease CHEMBL243 pIC50
Vitamin D receptor CHEMBL1977 pIC50
Human immunodeficiency virus type 1 protease CHEMBL243 pKi
Ghrelin receptor CHEMBL4616 pIC50

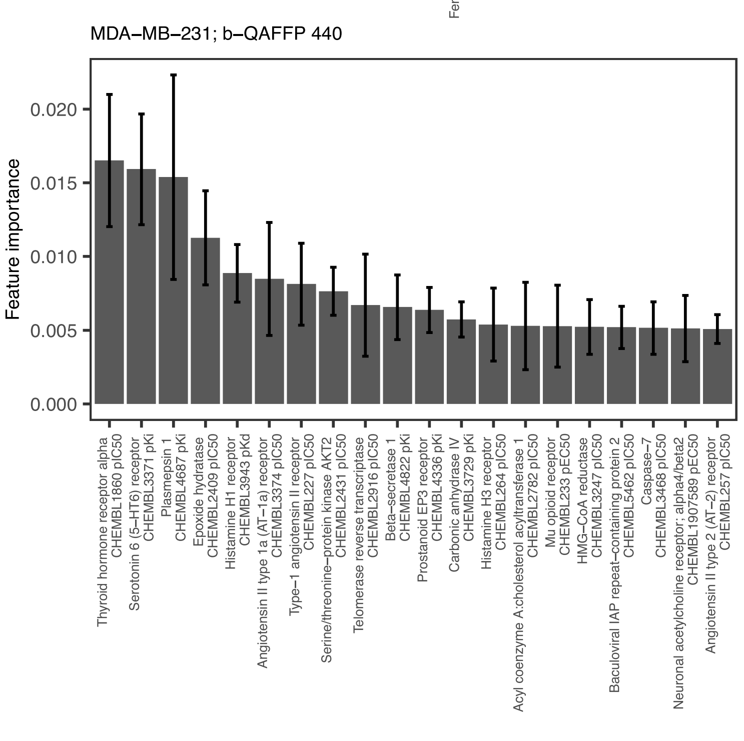
MDA–MB–231; b–QAFFP 440
Feature importance
0.020
0.015
0.010
0.005
0.000
Thyroid hormone receptor alpha CHEMBL1860 pIC50
Serotonin 6 (5–HT6) receptor CHEMBL3371 pKi
Plasmepsin 1 CHEMBL4687 pKi
Epoxide hydratase CHEMBL2409 pIC50
Histamine H1 receptor CHEMBL3943 pKd
Angiotensin II type 1a (AT–1a) receptor CHEMBL3374 pIC50
Type–1 angiotensin II receptor CHEMBL227 pIC50
Serine/threonine–protein kinase AKT2 CHEMBL2431 pIC50
Telomerase reverse transcriptase CHEMBL2916 pIC50
Beta–secretase 1 CHEMBL4822 pIC50
Prostanoid EP3 receptor CHEMBL4336 pIC50
Carbonic anhydrase IV CHEMBL3729 pKi
Histamine H3 receptor CHEMBL264 pIC50
Acyl coenzyme A:cholesterol acyltransferase 1 CHEMBL2782 pIC50
Mu opioid receptor CHEMBL233 pEC50
HMG–CoA reductase CHEMBL3247 pIC50
Baculoviral IAP repeat–containing protein 2 CHEMBL5462 pIC50
Caspase–7 CHEMBL3468 pIC50
Neuronal acetylcholine receptor; alpha4/beta2 CHEMBL1907589 pEC50
Angiotensin II type 2 (AT–2) receptor CHEMBL257 pIC50

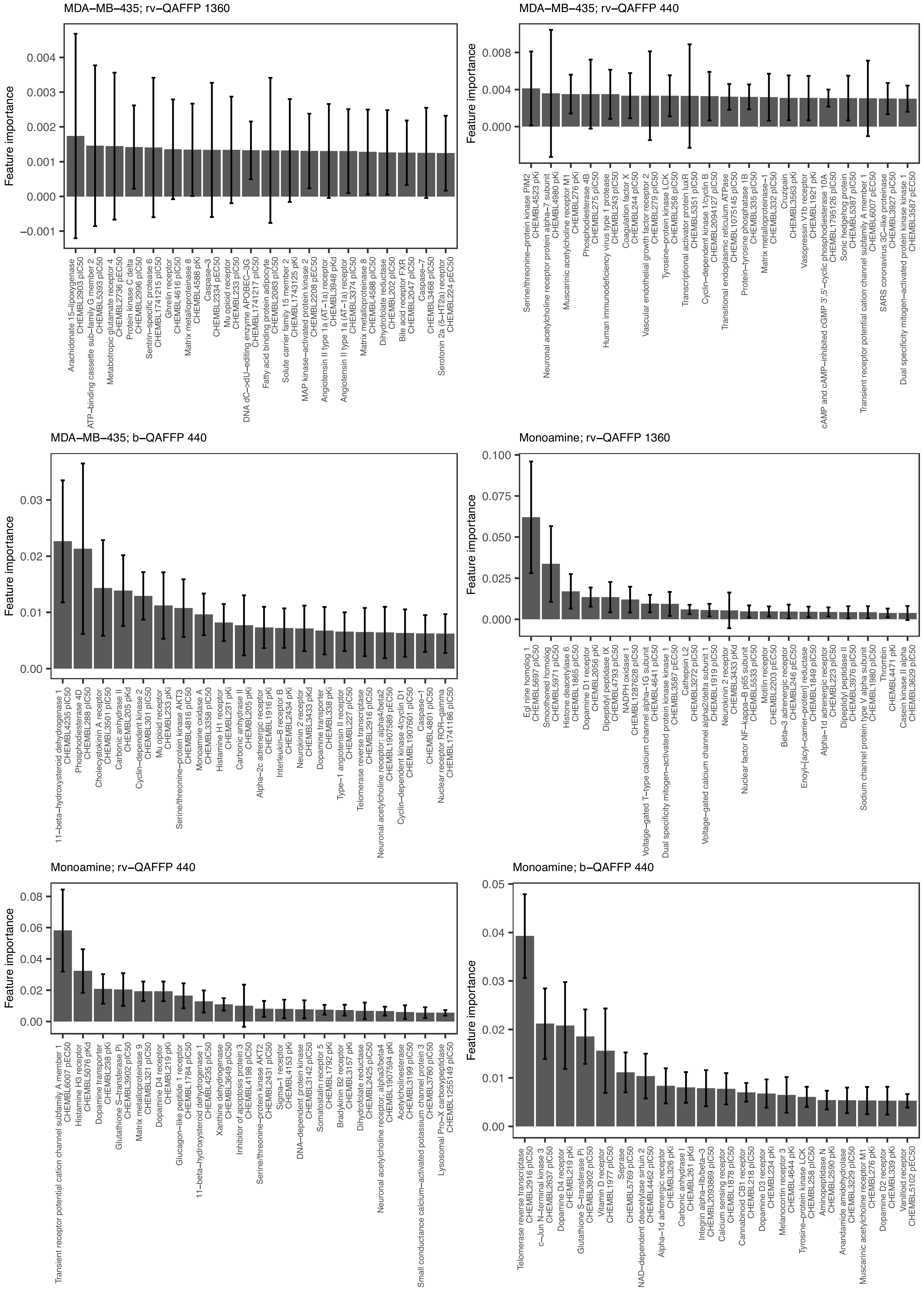
MDA−MB−435; rv−QAFFP 1360
Feature importance
Arachidonate 15−lipoxygenase CHEMBL2903 pIC50
ATP−binding cassette sub−family G member 2 CHEMBL5393 pIC50
Metabotropic glutamate receptor 4 CHEMBL2736 pEC50
Protein kinase C delta CHEMBL2996 pIC50
Sentrin−specific protease 6 CHEMBL1741215 pIC50
Ghrelin receptor CHEMBL4616 pIC50
Matrix metalloproteinase 8 CHEMBL4588 pKi
Caspase−3 CHEMBL2334 pEC50
Mu opioid receptor CHEMBL233 pIC50
DNA dC−>dU−editing enzyme APOBEC−3G CHEMBL1741217 pIC50
Fatty acid binding protein adipocyte CHEMBL2083 pIC50
Solute carrier family 15 member 2 CHEMBL1743125 pKi
MAP kinase−activated protein kinase 2 CHEMBL2208 pIC50
Angiotensin II type 1a (AT−1a) receptor CHEMBL3948 pKd
Angiotensin II type 1a (AT−1a) receptor CHEMBL3374 pIC50
Matrix metalloproteinase 8 CHEMBL4588 pIC50
Dihydrofolate reductase CHEMBL202 pIC50
Bile acid receptor FXR CHEMBL2047 pIC50
Caspase−7 CHEMBL3468 pIC50
Serotonin 2a (5−HT2a) receptor CHEMBL224 pEC50
MDA−MB−435; rv−QAFFP 440
Feature importance
Serine/threonine−protein kinase PIM2 CHEMBL4523 pKi
Neuronal acetylcholine receptor protein alpha−7 subunit CHEMBL4980 pKi
Muscarinic acetylcholine receptor M1 CHEMBL276 pKi
Phosphodiesterase 4B CHEMBL275 pIC50
Human immunodeficiency virus type 1 protease CHEMBL243 pIC50
Coagulation factor X CHEMBL244 pIC50
Vascular endothelial growth factor receptor 2 CHEMBL279 pIC50
Tyrosine−protein kinase LCK CHEMBL258 pIC50
Transcriptional activator protein luxR CHEMBL5351 pIC50
Cyclin−dependent kinase 1/cyclin B CHEMBL2094127 pIC50
Transitional endoplasmic reticulum ATPase CHEMBL1075145 pIC50
Protein−tyrosine phosphatase 1B CHEMBL335 pIC50
Matrix metalloproteinase−1 CHEMBL332 pIC50
Cruzipain CHEMBL3563 pKi
Vasopressin V1b receptor CHEMBL1921 pKi
cAMP and cAMP−inhibited cGMP 3',5'−cyclic phosphodiesterase 10A CHEMBL1795126 pIC50
Sonic hedgehog protein CHEMBL5387 pIC50
Transient receptor potential cation channel subfamily A member 1 CHEMBL6007 pEC50
SARS coronavirus 3C−like proteinase CHEMBL3927 pIC50
Dual specificity mitogen−activated protein kinase kinase 1 CHEMBL3587 pEC50
MDA−MB−435; b−QAFFP 440
Feature importance
11−beta−hydroxysteroid dehydrogenase 1 CHEMBL4235 pIC50
Phosphodiesterase 4D CHEMBL288 pIC50
Cholecystokinin A receptor CHEMBL3501 pIC50
Carbonic anhydrase II CHEMBL205 pKd
Cyclin−dependent kinase 2 CHEMBL301 pIC50
Mu opioid receptor CHEMBL233 pKi
Serine/threonine−protein kinase AKT3 CHEMBL4816 pIC50
Monoamine oxidase A CHEMBL3358 pIC50
Histamine H1 receptor CHEMBL231 pKi
Carbonic anhydrase II CHEMBL205 pKi
Alpha−2c adrenergic receptor CHEMBL1916 pKi
Interleukin−8 receptor B CHEMBL2434 pKi
Neurokinin 2 receptor CHEMBL3433 pKd
Dopamine transporter CHEMBL338 pKi
Type−1 angiotensin II receptor CHEMBL227 pIC50
Telomerase reverse transcriptase CHEMBL2916 pIC50
Neuronal acetylcholine receptor; alpha4/beta2 CHEMBL1907589 pEC50
Cyclin−dependent kinase 4/cyclin D1 CHEMBL1907601 pIC50
Caspase−1 CHEMBL4801 pIC50
Nuclear receptor ROR−gamma CHEMBL1741186 pIC50
Monoamine; rv−QAFFP 1360
Feature importance
Egl nine homolog 1 CHEMBL5697 pIC50
Smoothened homolog CHEMBL5971 pIC50
Histone deacetylase 6 CHEMBL1865 pIC50
Dopamine D1 receptor CHEMBL2056 pKi
Dipeptidyl peptidase IX CHEMBL4793 pIC50
NADPH oxidase 1 CHEMBL1287628 pIC50
Voltage−gated T−type calcium channel alpha−1G subunit CHEMBL4641 pIC50
Dual specificity mitogen−activated protein kinase kinase 1 CHEMBL3587 pIC50
Cathepsin L2 CHEMBL3272 pIC50
Voltage−gated calcium channel alpha2/delta subunit 1 CHEMBL1919 pIC50
Neurokinin 2 receptor CHEMBL3433 pKd
Nuclear factor NF−kappa−B p65 subunit CHEMBL5533 pIC50
Motilin receptor CHEMBL2203 pEC50
Beta−3 adrenergic receptor CHEMBL246 pEC50
Enoyl−[acyl−carrier−protein] reductase CHEMBL1849 pIC50
Alpha−1d adrenergic receptor CHEMBL223 pIC50
Dipeptidyl peptidase II CHEMBL3976 pIC50
Sodium channel protein type V alpha subunit CHEMBL1980 pIC50
Thrombin CHEMBL4471 pKi
Casein kinase II alpha CHEMBL3629 pIC50
Monoamine; rv−QAFFP 440
Feature importance
Transient receptor potential cation channel subfamily A member 1 CHEMBL6007 pEC50
Histamine H3 receptor CHEMBL5076 pKd
Dopamine transporter CHEMBL238 pKi
Glutathione S−transferase Pi CHEMBL3902 pIC50
Matrix metalloproteinase 9 CHEMBL321 pIC50
Dopamine D4 receptor CHEMBL219 pKi
Glucagon−like peptide 1 receptor CHEMBL1784 pIC50
11−beta−hydroxysteroid dehydrogenase 1 CHEMBL4235 pIC50
Xanthine dehydrogenase CHEMBL3649 pIC50
Inhibitor of apoptosis protein 3 CHEMBL4198 pIC50
Serine/threonine−protein kinase AKT2 CHEMBL2431 pIC50
Sigma−1 receptor CHEMBL4153 pKi
DNA−dependent protein kinase CHEMBL3142 pIC50
Somatostatin receptor 5 CHEMBL1792 pKi
Bradykinin B2 receptor CHEMBL3157 pKi
Dihydrofolate reductase CHEMBL2425 pIC50
Neuronal acetylcholine receptor; alpha3/beta4 CHEMBL1907594 pIC50
Acetylcholinesterase CHEMBL3199 pIC50
Small conductance calcium−activated potassium channel protein 3 CHEMBL3780 pIC50
Lysosomal Pro−X carboxypeptidase CHEMBL1255149 pIC50
Monoamine; b−QAFFP 440
Feature importance
Telomerase reverse transcriptase CHEMBL2916 pIC50
c−Jun N−terminal kinase 3 CHEMBL2637 pIC50
Dopamine D4 receptor CHEMBL219 pKi
Glutathione S−transferase Pi CHEMBL3902 pIC50
Vitamin D receptor CHEMBL1977 pIC50
Seprase CHEMBL5769 pIC50
NAD−dependent deacetylase sirtuin 2 CHEMBL4462 pIC50
Alpha−1d adrenergic receptor CHEMBL326 pKi
Carbonic anhydrase I CHEMBL261 pKd
Integrin alpha−IIb/beta−3 CHEMBL2093869 pIC50
Calcium sensing receptor CHEMBL1878 pIC50
Cannabinoid CB1 receptor CHEMBL218 pIC50
Dopamine D3 receptor CHEMBL234 pKi
Melanocortin receptor 3 CHEMBL4644 pKi
Tyrosine−protein kinase LCK CHEMBL258 pIC50
Aminopeptidase N CHEMBL2590 pKi
Anandamide amidohydrolase CHEMBL3229 pIC50
Muscarinic acetylcholine receptor M1 CHEMBL276 pKi
Dopamine D2 receptor CHEMBL339 pKi
Vanilloid receptor CHEMBL5102 pEC50

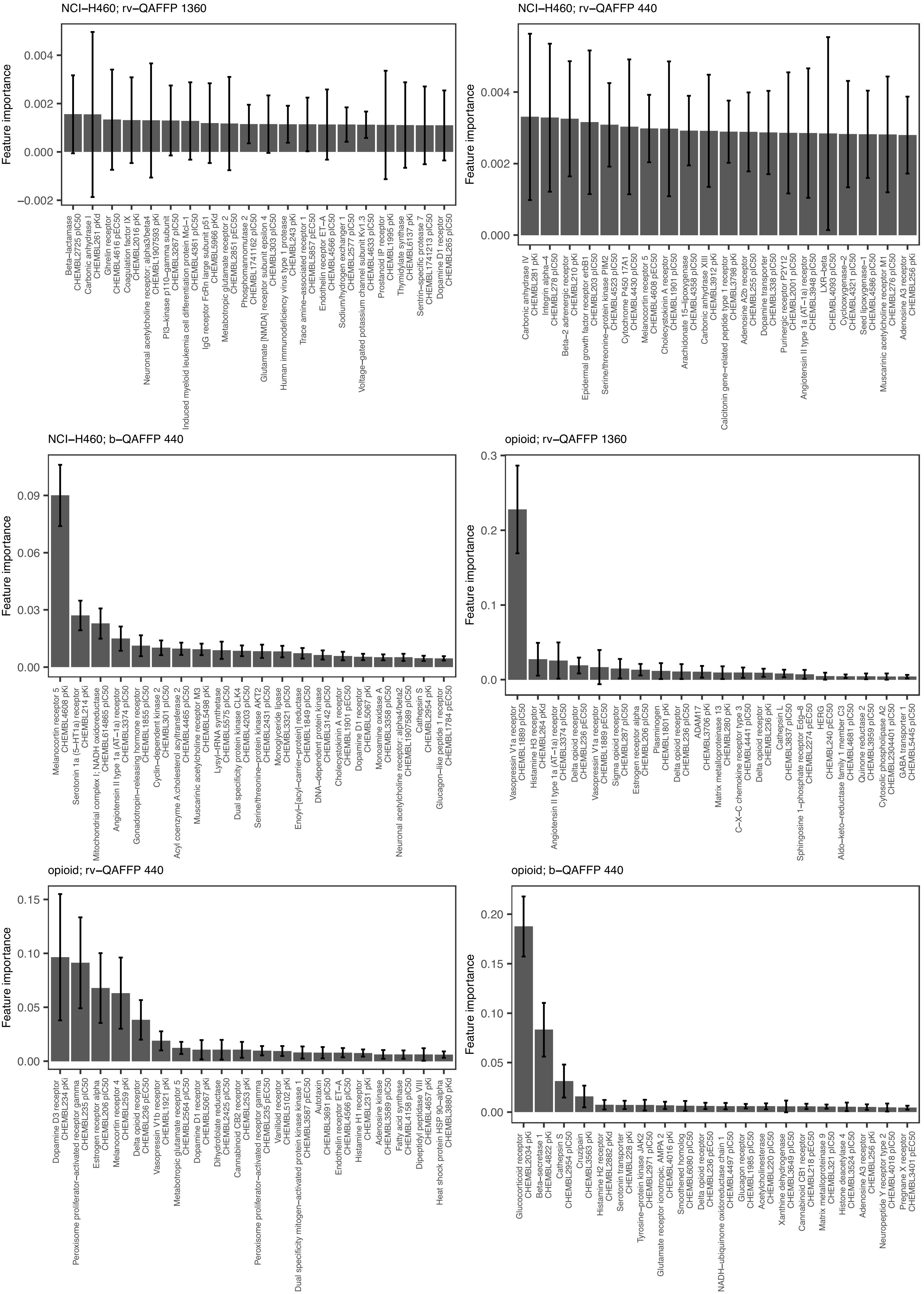
NCI–H460; rv–QAFFP 1360
Feature importance
0.004
0.002
0.000
–0.002
Beta–lactamase CHEMBL2725 pIC50
Carbonic anhydrase I CHEMBL261 pKd
Ghrelin receptor CHEMBL4616 pEC50
Coagulation factor IX CHEMBL2016 pKi
Neuronal acetylcholine receptor; alpha3/beta4 CHEMBL1907593 pKi
PI3–kinase p110–gamma subunit CHEMBL3267 pIC50
Induced myeloid leukemia cell differentiation protein Mcl–1 CHEMBL4361 pIC50
IgG receptor FcRn large subunit p51 CHEMBL5966 pKd
Metabotropic glutamate receptor 2 CHEMBL2851 pEC50
Phosphomannomutase 2 CHEMBL1741162 pIC50
Glutamate [NMDA] receptor subunit epsilon 4 CHEMBL303 pIC50
Human immunodeficiency virus type 1 protease CHEMBL243 pKi
Trace amine–associated receptor 1 CHEMBL5857 pEC50
Endothelin receptor ET–A CHEMBL4566 pIC50
Sodium/hydrogen exchanger 1 CHEMBL2577 pIC50
Voltage–gated potassium channel subunit Kv1.3 CHEMBL4633 pIC50
Prostanoid IP receptor CHEMBL1995 pKi
Thymidylate synthase CHEMBL6137 pKi
Sentrin–specific protease 7 CHEMBL1741213 pIC50
Dopamine D1 receptor CHEMBL265 pIC50
NCI–H460; rv–QAFFP 440
Feature importance
0.004
0.002
0.000
Carbonic anhydrase IV CHEMBL281 pKi
Integrin alpha–4 CHEMBL278 pIC50
Beta–2 adrenergic receptor CHEMBL210 pKi
Epidermal growth factor receptor erbB1 CHEMBL203 pIC50
Serine/threonine–protein kinase PIM2 CHEMBL4523 pIC50
Cytochrome P450 17A1 CHEMBL4430 pIC50
Melanocortin receptor 5 CHEMBL4608 pEC50
Cholecystokinin A receptor CHEMBL1901 pIC50
Arachidonate 15–lipoxygenase CHEMBL4358 pIC50
Carbonic anhydrase XIII CHEMBL3912 pKi
Calcitonin gene–related peptide type 1 receptor CHEMBL3798 pKi
Adenosine A2b receptor CHEMBL255 pIC50
Dopamine transporter CHEMBL338 pIC50
Purinergic receptor P2Y12 CHEMBL2001 pIC50
Angiotensin II type 1a (AT–1a) receptor CHEMBL3948 pIC50
LXR–beta CHEMBL4093 pIC50
Cyclooxygenase–2 CHEMBL4321 pIC50
Seed lipoxygenase–1 CHEMBL4586 pIC50
Muscarinic acetylcholine receptor M1 CHEMBL276 pIC50
Adenosine A3 receptor CHEMBL256 pKi
NCI–H460; b–QAFFP 440
Feature importance
0.09
0.06
0.03
0.00
Melanocortin receptor 5 CHEMBL4608 pKi
Serotonin 1a (5–HT1a) receptor CHEMBL214 pKi
Mitochondrial complex I; NADH oxidoreductase CHEMBL614865 pIC50
Angiotensin II type 1a (AT–1a) receptor CHEMBL3374 pIC50
Gonadotropin–releasing hormone receptor CHEMBL1855 pIC50
Cyclin–dependent kinase 2 CHEMBL301 pIC50
Acyl coenzyme A:cholesterol acyltransferase 2 CHEMBL4465 pIC50
Muscarinic acetylcholine receptor M3 CHEMBL5498 pKi
Lysyl–tRNA synthetase CHEMBL5575 pIC50
Dual specificity protein kinase CLK4 CHEMBL4203 pIC50
Serine/threonine–protein kinase AKT2 CHEMBL2431 pIC50
Monoglyceride lipase CHEMBL3321 pIC50
Enoyl–[acyl–carrier–protein] reductase CHEMBL1849 pIC50
DNA–dependent protein kinase CHEMBL3142 pIC50
Cholecystokinin A receptor CHEMBL1901 pEC50
Dopamine D1 receptor CHEMBL5067 pKi
Monoamine oxidase A CHEMBL3358 pIC50
Neuronal acetylcholine receptor; alpha4/beta2 CHEMBL1907589 pIC50
Cathepsin S CHEMBL2954 pKi
Glucagon–like peptide 1 receptor CHEMBL1784 pEC50
opioid; rv–QAFFP 1360
Feature importance
0.3
0.2
0.1
0.0
Vasopressin V1a receptor CHEMBL1889 pIC50
Histamine H3 receptor CHEMBL264 pKd
Angiotensin II type 1a (AT–1a) receptor CHEMBL3374 pIC50
Delta opioid receptor CHEMBL236 pEC50
Vasopressin V1a receptor CHEMBL1889 pEC50
Sigma opioid receptor CHEMBL287 pIC50
Estrogen receptor alpha CHEMBL206 pIC50
Plasminogen CHEMBL1801 pKi
Delta opioid receptor CHEMBL236 pIC50
ADAM17 CHEMBL3706 pKi
Matrix metalloproteinase 13 CHEMBL280 pKi
C–X–C chemokine receptor type 3 CHEMBL4441 pIC50
Delta opioid receptor CHEMBL236 pKi
Cathepsin L CHEMBL3837 pIC50
Sphingosine 1–phosphate receptor Edg–8 CHEMBL2274 pEC50
HERG CHEMBL240 pEC50
Aldo–keto–reductase family 1 member C3 CHEMBL4681 pIC50
Quinone reductase 2 CHEMBL3959 pIC50
Cytosolic phospholipase A2 CHEMBL2304401 pIC50
GABA transporter 1 CHEMBL5445 pIC50
opioid; rv–QAFFP 440
Feature importance
0.15
0.10
0.05
0.00
Dopamine D3 receptor CHEMBL234 pKi
Peroxisome proliferator–activated receptor gamma CHEMBL235 pIC50
Estrogen receptor alpha CHEMBL206 pIC50
Melanocortin receptor 4 CHEMBL259 pKi
Delta opioid receptor CHEMBL236 pEC50
Vasopressin V1b receptor CHEMBL1921 pKi
Metabotropic glutamate receptor 5 CHEMBL2564 pIC50
Dopamine D1 receptor CHEMBL5067 pKi
Dihydrofolate reductase CHEMBL2425 pIC50
Cannabinoid CB2 receptor CHEMBL253 pKi
Peroxisome proliferator–activated receptor gamma CHEMBL235 pEC50
Vanilloid receptor CHEMBL5102 pKi
Dual specificity mitogen–activated protein kinase kinase 1 CHEMBL3587 pEC50
Autotaxin CHEMBL3691 pIC50
Endothelin receptor ET–A CHEMBL4566 pIC50
Histamine H1 receptor CHEMBL231 pKi
Adenosine kinase CHEMBL3589 pIC50
Fatty acid synthase CHEMBL4158 pIC50
Dipeptidyl peptidase VIII CHEMBL4657 pKi
Heat shock protein HSP 90–alpha CHEMBL3880 pKd
opioid; b–QAFFP 440
Feature importance
0.20
0.15
0.10
0.05
0.00
Glucocorticoid receptor CHEMBL2034 pKi
Beta–secretase 1 CHEMBL4822 pKi
Cathepsin S CHEMBL2954 pIC50
Cruzipain CHEMBL3563 pKi
Histamine H2 receptor CHEMBL2882 pKd
Serotonin transporter CHEMBL228 pKi
Tyrosine–protein kinase JAK2 CHEMBL2971 pIC50
Glutamate receptor ionotropic, AMPA 2 CHEMBL4016 pKi
Smoothened homolog CHEMBL6080 pIC50
Delta opioid receptor CHEMBL236 pEC50
NADH–ubiquinone oxidoreductase chain 1 CHEMBL4497 pIC50
Glucagon receptor CHEMBL1985 pIC50
Acetylcholinesterase CHEMBL220 pIC50
Xanthine dehydrogenase CHEMBL3649 pIC50
Cannabinoid CB1 receptor CHEMBL218 pEC50
Matrix metalloproteinase 9 CHEMBL321 pIC50
Histone deacetylase 4 CHEMBL3524 pIC50
Adenosine A3 receptor CHEMBL256 pKi
Neuropeptide Y receptor type 2 CHEMBL4018 pIC50
Pregnane X receptor CHEMBL3401 pEC50

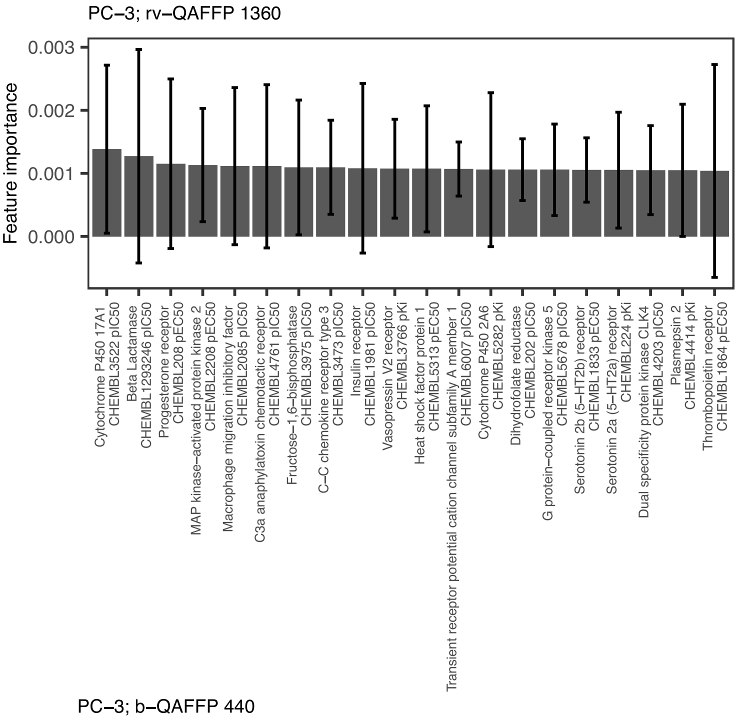
PC−3; rv−QAFFP 1360
Feature importance
0.003
0.002
0.001
0.000
Cytochrome P450 17A1 CHEMBL3522 pIC50
Beta Lactamase CHEMBL1293246 pIC50
Progesterone receptor CHEMBL208 pEC50
MAP kinase−activated protein kinase 2 CHEMBL2208 pEC50
Macrophage migration inhibitory factor CHEMBL2085 pIC50
C3a anaphylatoxin chemotactic receptor CHEMBL4761 pIC50
Fructose−1,6−bisphosphatase CHEMBL3975 pIC50
C−C chemokine receptor type 3 CHEMBL3473 pIC50
Insulin receptor CHEMBL1981 pIC50
Vasopressin V2 receptor CHEMBL3766 pKi
Heat shock factor protein 1 CHEMBL5313 pEC50
Transient receptor potential cation channel subfamily A member 1 CHEMBL6007 pIC50
Cytochrome P450 2A6 CHEMBL5282 pKi
Dihydrofolate reductase CHEMBL202 pIC50
G protein−coupled receptor kinase 5 CHEMBL5678 pIC50
Serotonin 2b (5−HT2b) receptor CHEMBL1833 pEC50
Serotonin 2a (5−HT2a) receptor CHEMBL224 pKi
Dual specificity protein kinase CLK4 CHEMBL4203 pIC50
Plasmepsin 2 CHEMBL4414 pKi
Thrombopoietin receptor CHEMBL1864 pEC50

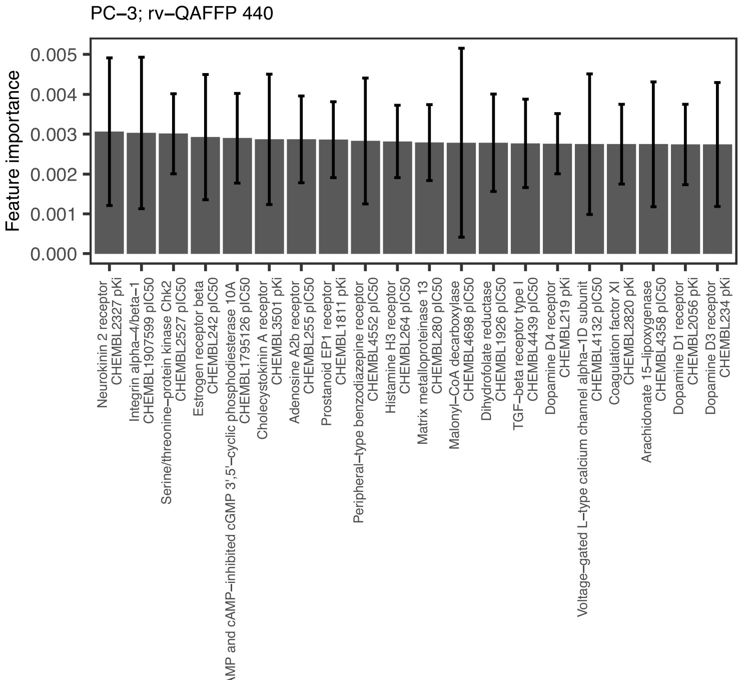
PC−3; rv−QAFFP 440
Feature importance
0.005
0.004
0.003
0.002
0.001
0.000
Neurokinin 2 receptor CHEMBL2327 pKi
Integrin alpha−4/beta−1 CHEMBL1907599 pIC50
Serine/threonine−protein kinase Chk2 CHEMBL2527 pIC50
Estrogen receptor beta CHEMBL242 pIC50
cAMP and cAMP−inhibited cGMP 3',5'−cyclic phosphodiesterase 10A CHEMBL1795126 pIC50
Cholecystokinin A receptor CHEMBL3501 pKi
Adenosine A2b receptor CHEMBL255 pIC50
Prostanoid EP1 receptor CHEMBL1811 pKi
Peripheral−type benzodiazepine receptor CHEMBL4552 pIC50
Histamine H3 receptor CHEMBL264 pIC50
Matrix metalloproteinase 13 CHEMBL280 pIC50
Malonyl−CoA decarboxylase CHEMBL4698 pIC50
Dihydrofolate reductase CHEMBL1926 pIC50
TGF−beta receptor type I CHEMBL4439 pIC50
Dopamine D4 receptor CHEMBL219 pKi
Voltage−gated L−type calcium channel alpha−1D subunit CHEMBL4132 pIC50
Coagulation factor XI CHEMBL2820 pIC50
Arachidonate 15−lipoxygenase CHEMBL4358 pIC50
Dopamine D1 receptor CHEMBL2056 pKi
Dopamine D3 receptor CHEMBL234 pKi

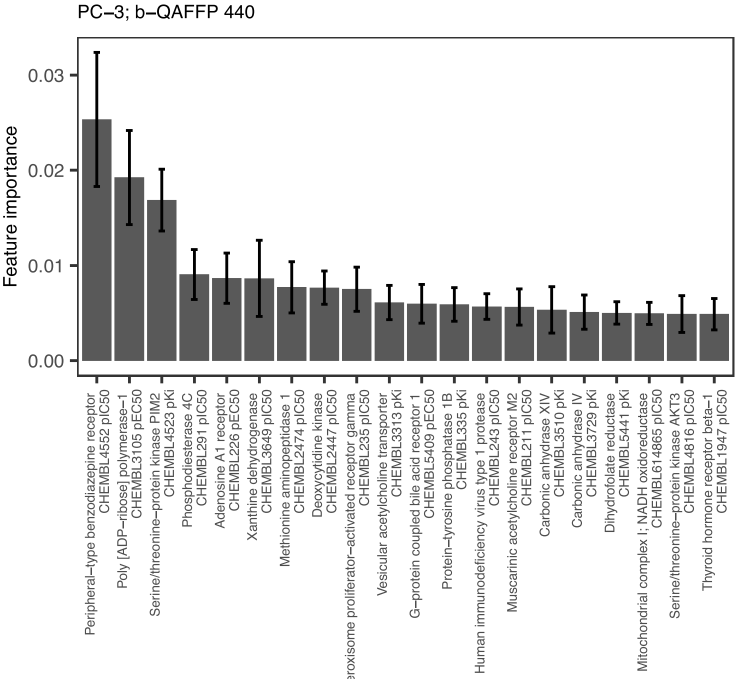
PC−3; b−QAFFP 440
Feature importance
0.03
0.02
0.01
0.00
Peripheral−type benzodiazepine receptor CHEMBL4552 pIC50
Poly [ADP−ribose] polymerase−1 CHEMBL3105 pEC50
Serine/threonine−protein kinase PIM2 CHEMBL4523 pKi
Phosphodiesterase 4C CHEMBL291 pIC50
Adenosine A1 receptor CHEMBL226 pEC50
Xanthine dehydrogenase CHEMBL3649 pIC50
Methionine aminopeptidase 1 CHEMBL2474 pIC50
Deoxycytidine kinase CHEMBL2447 pIC50
Peroxisome proliferator−activated receptor gamma CHEMBL235 pIC50
Vesicular acetylcholine transporter CHEMBL3313 pKi
G−protein coupled bile acid receptor 1 CHEMBL5409 pEC50
Protein−tyrosine phosphatase 1B CHEMBL335 pKi
Human immunodeficiency virus type 1 protease CHEMBL243 pIC50
Muscarinic acetylcholine receptor M2 CHEMBL211 pIC50
Carbonic anhydrase XIV CHEMBL3510 pKi
Carbonic anhydrase IV CHEMBL3729 pIC50
Dihydrofolate reductase CHEMBL5441 pKi
Mitochondrial complex I; NADH oxidoreductase CHEMBL614865 pIC50
Serine/threonine−protein kinase AKT3 CHEMBL4816 pIC50
Thyroid hormone receptor beta−1 CHEMBL1947 pIC50

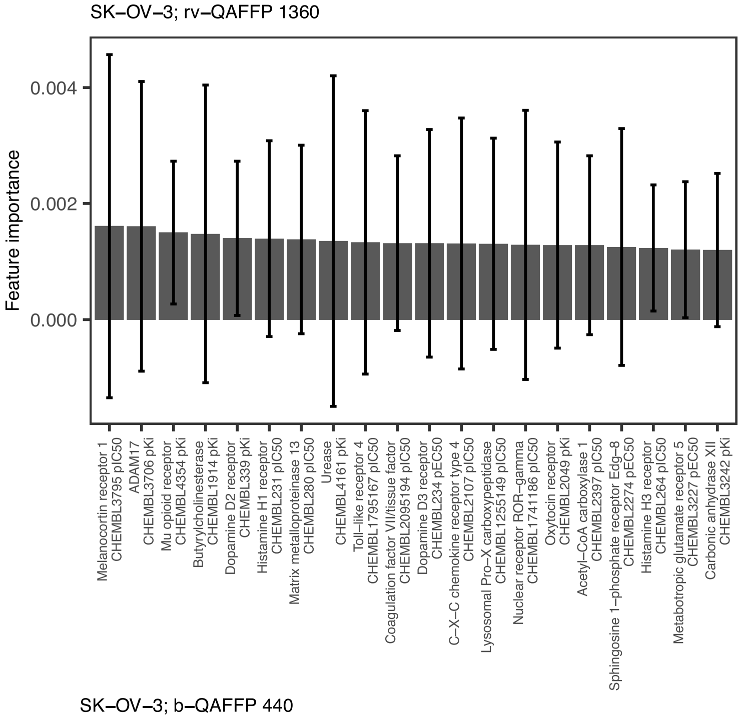
SK−OV−3; rv−QAFFP 1360
Feature importance
0.004
0.002
0.000
Melanocortin receptor 1 CHEMBL3795 pIC50
ADAM17 CHEMBL3706 pKi
Mu opioid receptor CHEMBL4354 pKi
Butyrylcholinesterase CHEMBL1914 pKi
Dopamine D2 receptor CHEMBL339 pKi
Histamine H1 receptor CHEMBL231 pIC50
Matrix metalloproteinase 13 CHEMBL280 pIC50
Urease CHEMBL4161 pKi
Toll−like receptor 4 CHEMBL1795167 pIC50
Coagulation factor VII/tissue factor CHEMBL2095194 pIC50
Dopamine D3 receptor CHEMBL234 pEC50
C−X−C chemokine receptor type 4 CHEMBL2107 pIC50
Lysosomal Pro−X carboxypeptidase CHEMBL1255149 pIC50
Nuclear receptor ROR−gamma CHEMBL1741186 pIC50
Oxytocin receptor CHEMBL2049 pKi
Acetyl−CoA carboxylase 1 CHEMBL2397 pIC50
Sphingosine 1−phosphate receptor Edg−8 CHEMBL2274 pEC50
Histamine H3 receptor CHEMBL264 pIC50
Metabotropic glutamate receptor 5 CHEMBL3227 pEC50
Carbonic anhydrase XII CHEMBL3242 pIC50

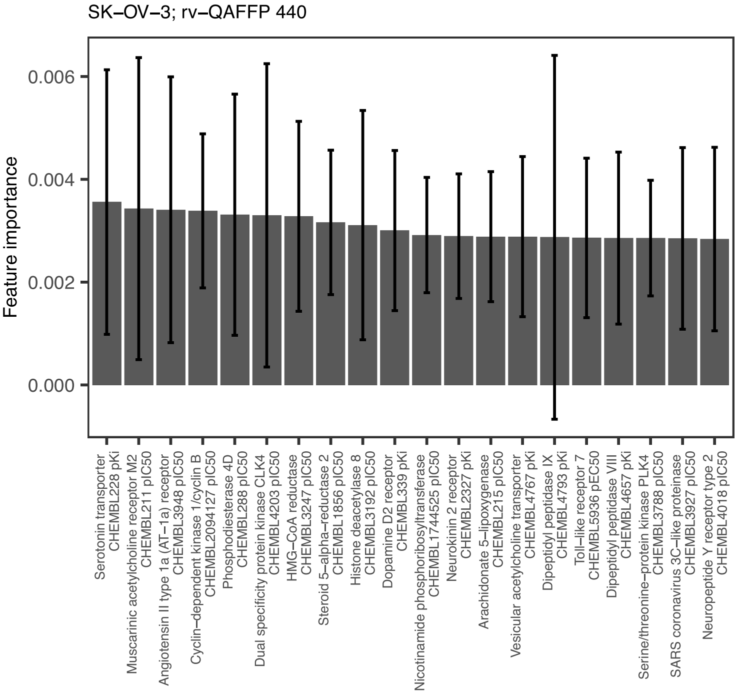
SK−OV−3; rv−QAFFP 440
Feature importance
0.006
0.004
0.002
0.000
Serotonin transporter CHEMBL228 pKi
Muscarinic acetylcholine receptor M2 CHEMBL211 pIC50
Angiotensin II type 1a (AT−1a) receptor CHEMBL3948 pIC50
Cyclin−dependent kinase 1/cyclin B CHEMBL2094127 pIC50
Phosphodiesterase 4D CHEMBL288 pIC50
Dual specificity protein kinase CLK4 CHEMBL4203 pIC50
HMG−CoA reductase CHEMBL3247 pIC50
Steroid 5−alpha−reductase 2 CHEMBL1856 pIC50
Histone deacetylase 8 CHEMBL3192 pIC50
Dopamine D2 receptor CHEMBL339 pKi
Nicotinamide phosphoribosyltransferase CHEMBL1744525 pIC50
Neurokinin 2 receptor CHEMBL2327 pKi
Arachidonate 5−lipoxygenase CHEMBL215 pIC50
Vesicular acetylcholine transporter CHEMBL4767 pKi
Dipeptidyl peptidase IX CHEMBL4793 pKi
Toll−like receptor 7 CHEMBL5936 pEC50
Dipeptidyl peptidase VIII CHEMBL4657 pKi
Serine/threonine−protein kinase PLK4 CHEMBL3788 pIC50
SARS coronavirus 3C−like proteinase CHEMBL3927 pIC50
Neuropeptide Y receptor type 2 CHEMBL4018 pIC50

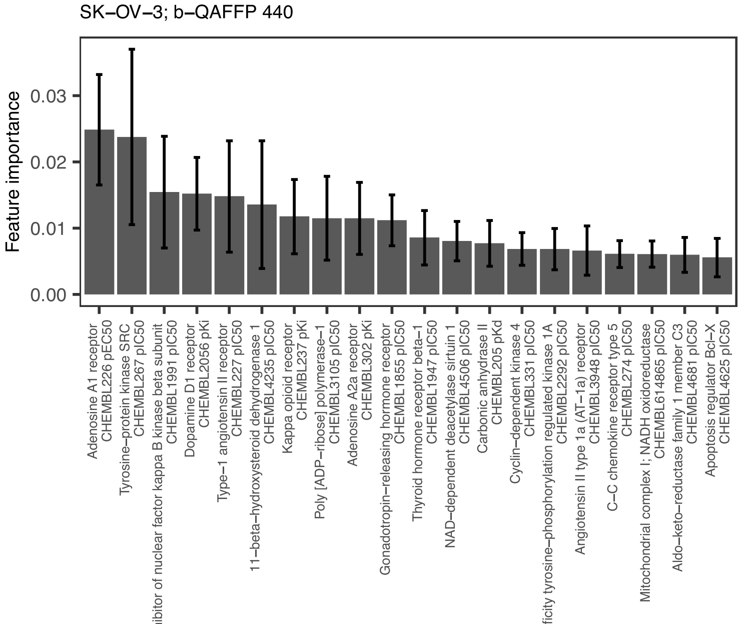
SK−OV−3; b−QAFFP 440
Feature importance
0.03
0.02
0.01
0.00
Adenosine A1 receptor CHEMBL226 pEC50
Tyrosine−protein kinase SRC CHEMBL267 pIC50
Inhibitor of nuclear factor kappa B kinase beta subunit CHEMBL1991 pIC50
Dopamine D1 receptor CHEMBL2056 pKi
Type−1 angiotensin II receptor CHEMBL227 pIC50
11−beta−hydroxysteroid dehydrogenase 1 CHEMBL4235 pIC50
Kappa opioid receptor CHEMBL237 pKi
Poly [ADP−ribose] polymerase−1 CHEMBL3105 pIC50
Adenosine A2a receptor CHEMBL302 pKi
Gonadotropin−releasing hormone receptor CHEMBL1855 pIC50
Thyroid hormone receptor beta−1 CHEMBL1947 pIC50
NAD−dependent deacetylase sirtuin 1 CHEMBL4506 pIC50
Carbonic anhydrase II CHEMBL205 pKd
Cyclin−dependent kinase 4 CHEMBL331 pIC50
Dual−specificity tyrosine−phosphorylation regulated kinase 1A CHEMBL2292 pIC50
Angiotensin II type 1a (AT−1a) receptor CHEMBL3948 pIC50
C−C chemokine receptor type 5 CHEMBL274 pIC50
Mitochondrial complex I; NADH oxidoreductase CHEMBL614865 pIC50
Aldo−keto−reductase family 1 member C3 CHEMBL4681 pIC50
Apoptosis regulator Bcl−X CHEMBL4625 pIC50

Supplementary Figure 7

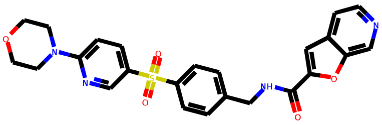

CHEMBL2420625

A2780

Morgan: μ=1.93, σ=0.29 pIC_{50} units

rv-QAFFP : μ=0.69, σ=0.16 pIC_{50} units

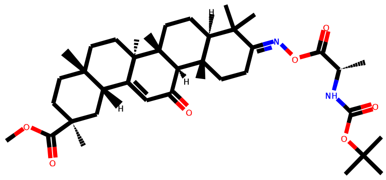

CHEMBL2151613

A2780

Morgan: μ=2.54, σ=0.29 pIC_{50} units

rv-QAFFP : μ=0.85, σ=0.13 pIC_{50} units

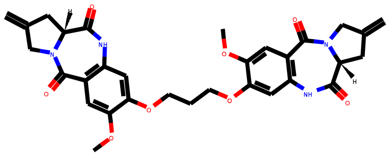

CHEMBL164587

A2780

Morgan: μ=1.93, σ=0.59 pIC_{50} units

rv-QAFFP : μ=1.19, σ=0.43 pIC_{50} units

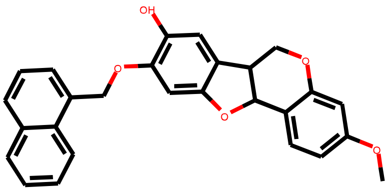

CHEMBL280404

CCRF-CEM

Morgan: μ=1.96, σ=0.26 pIC_{50} units

rv-QAFFP : μ=0.95, σ=0.14 pIC_{50} units

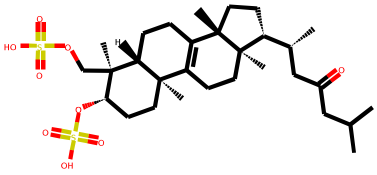

CHEMBL449476

HCT-116

Morgan: μ=1.87, σ=0.32 pIC_{50} units

rv-QAFFP : μ=0.97, σ=0.12 pIC_{50} units

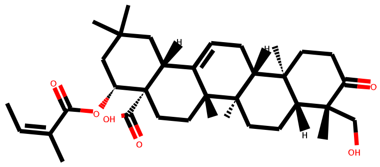

CHEMBL554122

HCT-116

Morgan: μ=1.14, σ=1.10 pIC_{50} units

rv-QAFFP : μ=0.41, σ=0.30 pIC_{50} units

Supplementary Figure 8

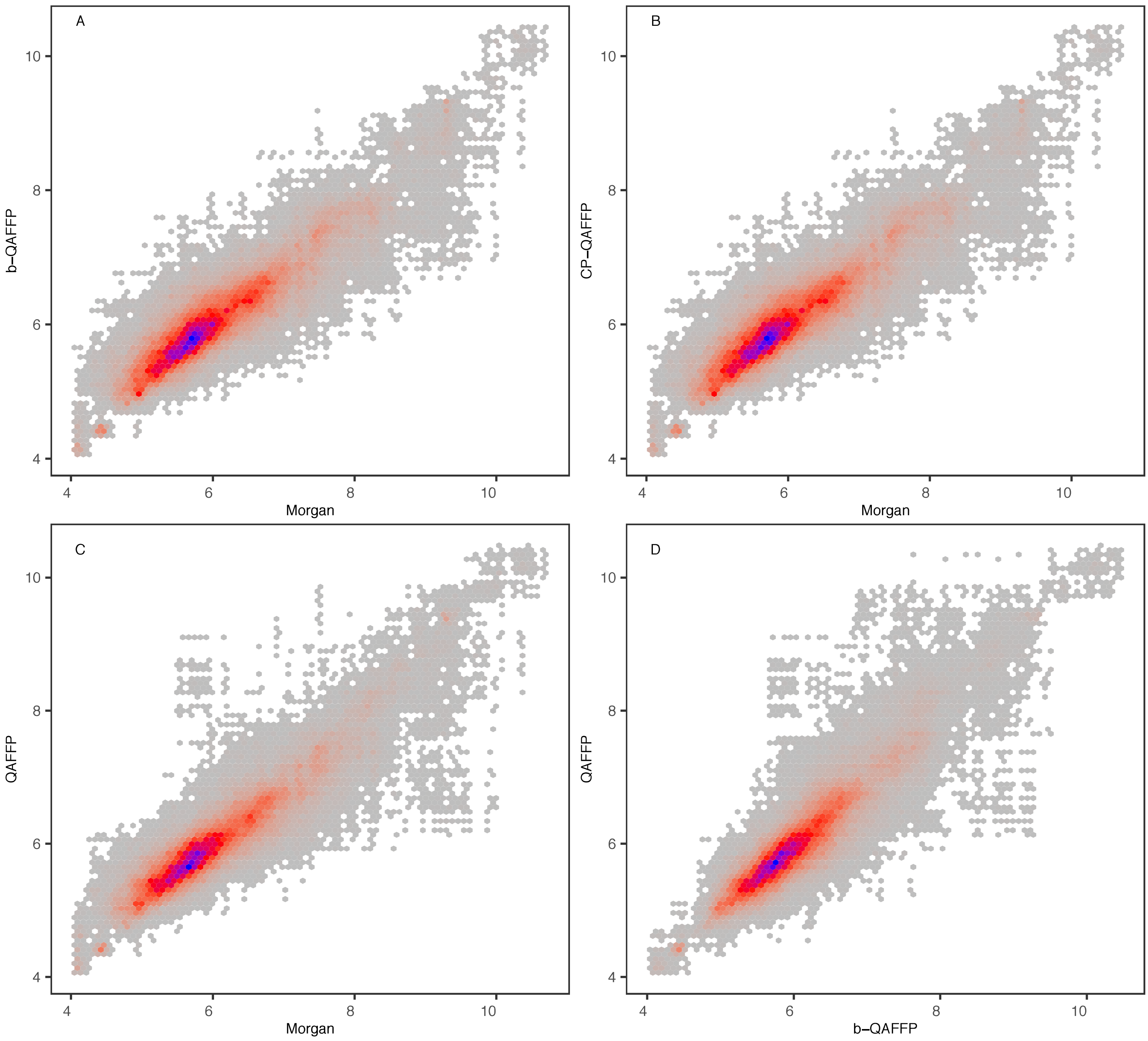

Supplement: Supplementary file 1 — Additional file 1: Figure S1. Distribution of pIC50 values for all data sets modelled in this study. Figure S2. Model performance on the test set as a function of the number of trees in the Random Forest models for a random selection of 18 data sets. Figure S3. R2test values calculated with models trained on each of the 11 descriptor types considered across the 43 data sets modelled in this study (related to Fig. 4). We trained 50 models for each combination of descriptor type and data set, each time holding a different subset of the data as test set. Figure S4. Y-scrambling experiments. R2test values calculated for models trained after shuffling the response variable are shown. Figure S5. RMSEtest values as a function of the fraction of the training data used as test set for all data sets. Figure S6. Mean variable importance +/− standard deviation averaged across 50 replicates. Only the top 20 descriptors are shown for each data set. Figure S7. Examples of compounds that were predicted with higher error by models trained on Morgan2 fingerprints than by models trained on rv-QAFFP 440. The predictions were calculated on the test set across 50 replicates. The mean and the standard deviation across these 50 replicates are shown. The data set is indicated below the compounds ChEMBL IDs. Figure S8. Predicted pIC50 values using models trained on the fingerprint type indicated in x-axis, against the predicted pIC50 values calculated using models trained using the fingerprint type indicated in the y-axis. The plot shows the predictions for 50 replicates for data set A2780. Similar results were obtained for the other data sets. Overall, it can be seen that the predictions generated by models trained using Morgan2 fingerprints and the rv-QAFFP 440 versions considered are highly correlated across the entire bioactivity range modelled. [file 13321_2020_444_MOESM1_ESM.pdf]
